# Wake-activated neuronal populations that regulate sleep drive

**DOI:** 10.1038/s41586-026-10928-3

**Published:** 2026-08-19

**Authors:** William Joo, Clare Diester, Vassilis Bitsikas, Myrto Panopoulou, Amelia Hidalgo, Konstantinos Ntemos, Rodrigo C. G. Pena, Fabia Imhof, Iris Odstrcil, Flavio Donato, Geoffrey Fucile, Daniel Kroeger, Thomas E. Scammell, Alexander F. Schier

**Affiliations:** 1 Biozentrum, University of Basel, Basel, Switzerland; 2 Department of Molecular and Cellular Biology, Harvard University, Cambridge, MA USA; 3 Center for Data Analytics, University of Basel, Basel, Switzerland; 4 Friedrich Miescher Institute for Biomedical Research (FMI), Basel, Switzerland; 5 Department of Anatomy, Physiology and Pharmacology, Auburn University, Auburn, AL USA; 6 Department of Neurology, Beth Israel Deaconess Medical Center, Boston, MA USA; 7 Department of Neurology, Boston Children’s Hospital, Boston, MA USA

**Keywords:** Sleep, Sleep deprivation, Neural circuits

## Abstract

Prolonged wakefulness increases sleep drive and is normally compensated for by increased sleep^1–3^. This homeostatic regulation of sleep shapes our lives profoundly, but the underlying neural circuit mechanisms remain poorly understood. Here, we identify wake-activated neurons that regulate sleep drive in mice, using whole-brain activity mapping, targeted neuronal manipulations and electrophysiology. By comparing whole-brain responses to sleep deprivation, recovery sleep and circadian behaviour, we identify the anterior medial preoptic area and the median raphe as candidate regions that encode sleep deficit. Activating sleep-deprivation-responsive cells in these regions induces increases in sleep duration and intensity that resemble recovery sleep. Conversely, inhibiting deprivation-responsive cells reduces sleep and abolishes the increased sleep propensity usually observed during deprivation. Neurons in the median raphe that are responsive to sleep deprivation project to subcortical sleep-associated regions and act through the preoptic hypothalamus. These deprivation-sensitive cells include serotonergic neurons and a distinct population of GABAergic neurons, whose intrinsic excitability increases during sleep deprivation. Co-activation of GABAergic and serotonergic neurons synergistically promotes sleep, whereas co-inhibition chronically decreases sleep by nearly 70%. Remarkably, most mice survive despite this marked reduction in sleep, without the compensatory increases in sleep drive or the behavioural deficits typically associated with severe sleep deprivation. Together, these results define neuronal populations that are activated during wakefulness and are crucial for sleep drive.

## Abstract (web summary)

Whole-brain activity mapping, targeted cell manipulations and electrophysiology in mice identify wake-activated neuronal populations that are crucial for sleep drive and can persistently reduce daily sleep amount.

## Main

Sleep drive increases with time spent awake, and eventually becomes irresistible. Sleep deprivation leads to increased attempts to sleep, and the non-rapid eye movement (NREM) sleep that follows deprivation is longer and deeper, as measured by electroencephalogram (EEG) oscillatory activity in the delta range^1–3^ (around 0.5–4 Hz). Despite the identification of broadly distributed neural circuits that regulate sleep and wakefulness^4–7^, the mechanisms that generate the powerful urge to sleep remain mysterious. Human and animal studies have identified genetic loci that regulate sleep amount or EEG features of sleep^8^, and mitochondrial redox state, phosphorylation and secreted factors are thought to track sleep deficit^9–12^, but the relevant neural circuit mechanisms are poorly defined. Here, we use comprehensive activation mapping and genetically targeted manipulations to identify specific neuronal populations that regulate sleep drive.

## Brain responses to sleep deprivation and recovery

**Fig. 1:**
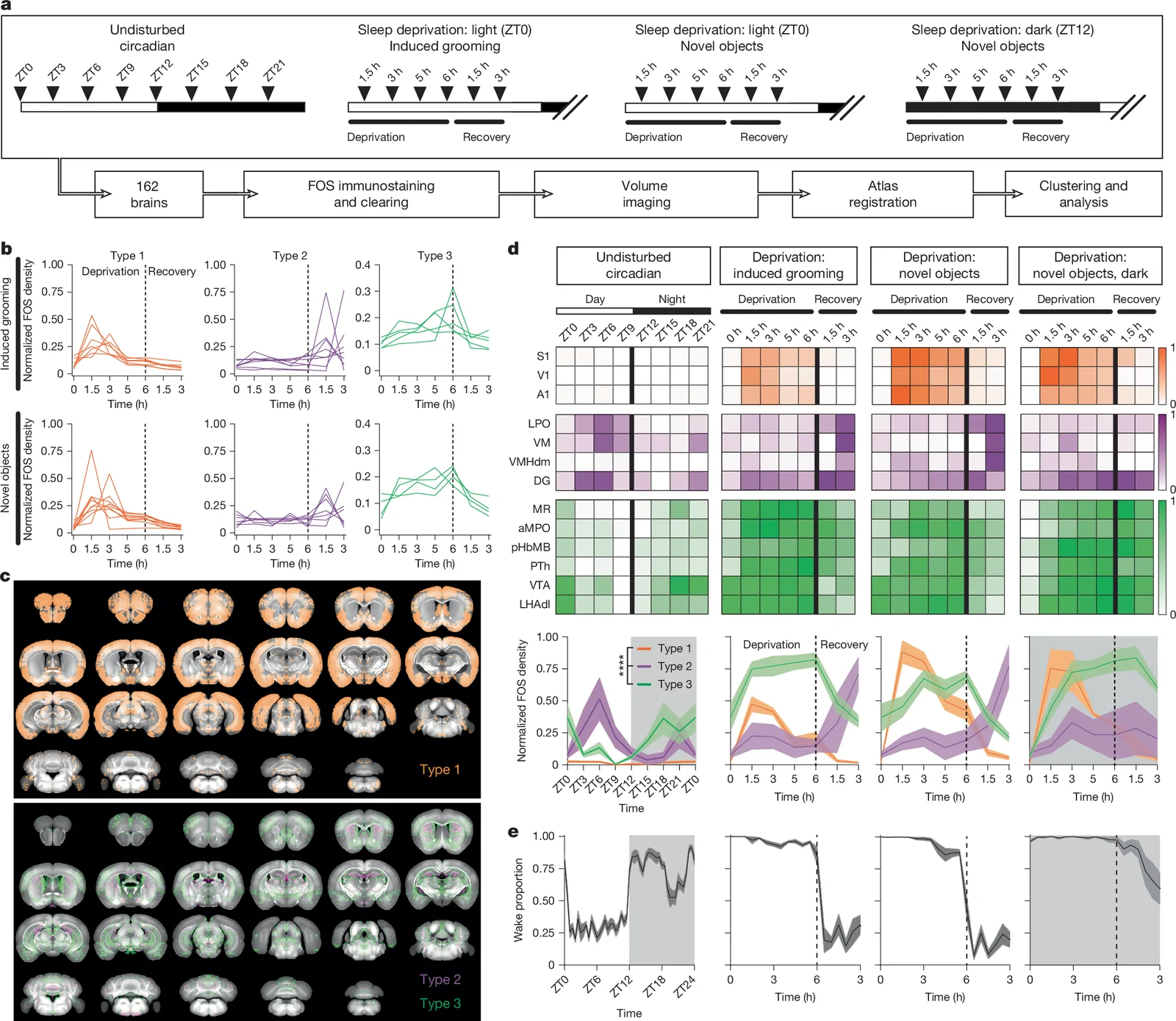
Mapping of whole-brain activity reveals correlates of sleep deprivation and recovery. **a**, Schematics of circadian and sleep-deprivation experiments. For unperturbed circadian comparisons, brains were collected every 3 h (black arrowheads) for FOS immunostaining, tissue clearing and volumetric imaging. ZT, zeitgeber time. Deprivation experiments included four time points during deprivation and two during recovery. *n* = 6 or 7 brains per time point for circadian and light-phase deprivation; *n* = 4 brains per time point for dark-phase deprivation. **b**, Mean response for each of 20 clusters per deprivation experiment, classified as type 1 (early peak; orange), type 2 (recovery peak; magenta) or type 3 (deprivation-responsive; green). Dotted line, 6-h deprivation time point. Top row, response types for induced grooming-based sleep deprivation. Bottom row, response types for novel-object-based sleep deprivation. **c**, Anatomical overview of type 1–3 responses common to grooming-based and novel-object-based sleep deprivation. **d**, Mean FOS responses for example subregions within type 1–3 clusters, rescaled for each region across all experiments and coloured as in **b**,**c**. Bottom row, responses across all regions, segregated by response type. Grey shading indicates dark phases (lights off). Mean ± s.e.m. ****P < 0.0001, response type versus time interaction for all pairwise comparisons in each experiment, two-way analysis of variance (ANOVA). A1, auditory cortex; DG, dentate gyrus; LHAdl, lateral hypothalamic area dorsolateral portion; pHbMB, peri-habenular midbrain; PTh, posterior thalamus; S1, somatosensory cortex; V1, visual cortex; VMHdm, ventromedial hypothalamus, dorsomedial portion; VTA, ventral tegmental area. See Extended Data Fig. 3. **e**, Proportion of time spent awake for undisturbed and sleep-deprived conditions, in the same order as in **d**. Mean ± s.e.m. Data for eight representative mice per condition. Source data

**Extended Data Fig. 1:**
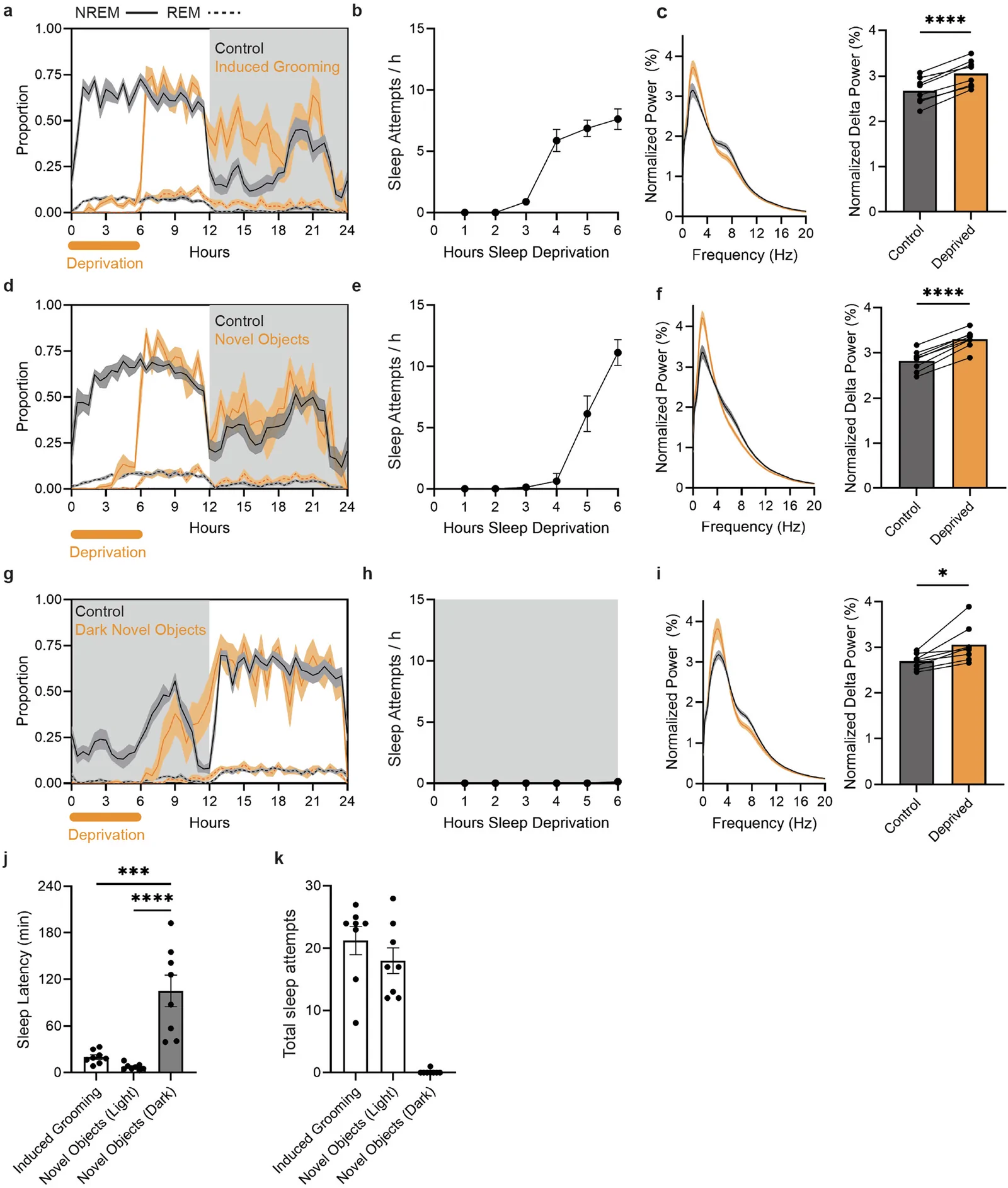
Comparison of sleep-deprivation methods. **a**, Proportion of time in NREM sleep (solid lines) or REM sleep (dotted lines) during undisturbed control conditions (grey) and during 6 h sleep deprivation using induced grooming (orange). Time 0 h is beginning of the light phase. A fine water mist was applied every 15 min. to evoke grooming behaviour. Sleep/wake states were quantified using EEG recordings here and throughout. **b**, Hourly sleep attempt number during the grooming-based deprivation period. Also see Extended Data Fig. 6. **c**, Induced grooming sleep deprivation increases slow-wave activity in the delta range (0.25–4 Hz). NREM power spectra (left) and delta power (right) during the 3 h following deprivation. **a**–**c**, n = 8 mice, ****p < 0.0001, paired two-tailed *t*-test. **d**, NREM and REM sleep during undisturbed control conditions (grey) and 6 h light phase sleep deprivation using novel objects (orange). Time 0 h is beginning of the light phase. Novel objects were switched every 15 min. to keep mice engaged. **e**, Hourly sleep attempt number during the novel objects-based deprivation period. **f**, Novel objects sleep deprivation increases delta power. NREM power spectra (left) and delta power (right) in the 3 h following deprivation. **d**–**f**, n = 8 mice, ***p < 0.0001, paired two-tailed *t*-test. **g**, NREM and REM sleep during unperturbed control conditions (grey) and during 6 h dark phase sleep deprivation using novel objects (orange). Time 0 h is beginning of the dark phase. **h**, Hourly sleep attempt number during the dark phase deprivation period. **i**, Novel objects sleep deprivation during the dark phase increases delta power. NREM power spectra (left) and delta power (right). Owing to longer sleep latency (see **j**), a 3 h window was quantified 2 h post-deprivation. **g**–**i**, n = 8 mice, *p = 0.0102, paired two-tailed *t*-test. **j**, Post-deprivation latency to the first NREM sleep bout. n = 8 mice per group, ***p = 0.0001, ****p < 0.0001, one-way ANOVA with Tukey’s multiple comparisons test. **k**, Total number of sleep attempts during the deprivation period. n = 8 mice per group. All data are mean ± s.e.m.

**Extended Data Fig. 2:**
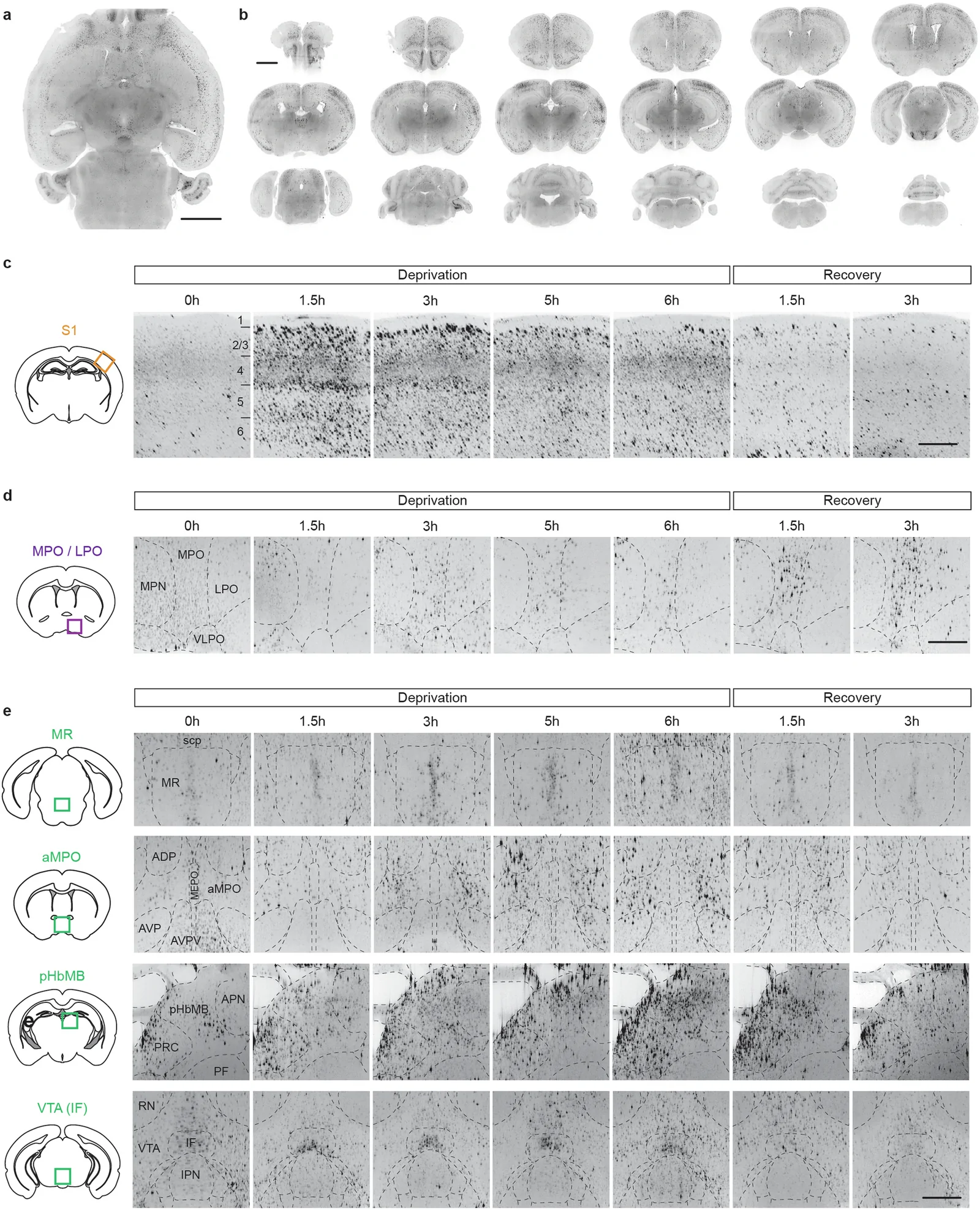
Representative light-sheet imaging data. **a**, Horizontal optical section of a representative mouse brain isolated 1.5 h into novel objects sleep deprivation, stained for FOS (greyscale), and cleared before imaging. Scale bar, 2 mm. **b**, Coronal views along the anterior-posterior axis of the brain. Scale bar, 2 mm. **c**, Coronal views of somatosensory cortex (S1) whisker barrel field, an example region with type 1 responses. Schematic of displayed region is shown on left. Representative examples of equivalent planes are shown for each time point, from left to right. **d**, Coronal views of the lateral preoptic area (LPO), an example region with sleep-correlated, type 2 responses. **e**, Coronal views of example regions with wake-correlated type 3 responses, schematized at left. MR, median raphe; aMPO, anterior medial preoptic area; pHbMB, peri-habenular midbrain; VTA, ventral tegmental area. Scale bars in **c**–**e**, 250 µm. Representative images in a-e were selected from n = 6–7 brains/time point for circadian and light phase deprivation, and from n = 4 brains per time point for dark phase deprivation. The brain schematics in **c**–**e** were adapted from ref. ^76^, Springer Nature Limited, under a Creative Commons licence CC BY 4.0.

To determine how the brain responds to prolonged wakefulness, we deprived mice of sleep for 6 h at the beginning of the light phase (the rest phase for mice) using either induced grooming behaviour or novel object exposure (Extended Data Fig. 1 and Methods). The immediate-early gene *Fos *labels activated neurons that control homeostatic behaviours such as feeding or drinking, and has previously revealed sleep-promoting or circadian-associated brain areas^13–23^. Accordingly, mice were euthanized for whole-brain FOS immunostaining, tissue clearing and light-sheet imaging at four time points during sleep deprivation and two time points during a 3-h recovery period, when mice exhibit post-deprivation rebound sleep (Fig. 1a and Extended Data Figs. 1 and 2). To control for stimulus-specific effects, we compared these regimes with sleep deprivation during the dark phase. In addition, we analysed 3-h circadian-time intervals to compare sleep deprivation with normal increases in sleep drive during unperturbed behaviour (Fig. 1a). After automated detection and registration of FOS^+^ nuclei to a common brain atlas^24,25^, voxel-wise clustering of FOS density across experimental time points revealed three main voxel response types during sleep deprivation and recovery: an early-peaking type that probably reflects the onset of sleep-deprivation stimuli (type 1); a sleep-correlated type (type 2); and a wake-correlated type that scales with wake duration (type 3). To support exploration of this dataset, which spans 162 brains over 26 conditions, we created a web resource, which is available at https://sleep-wake-atlas.scicore.unibas.ch/.

**Extended Data Fig. 3:**
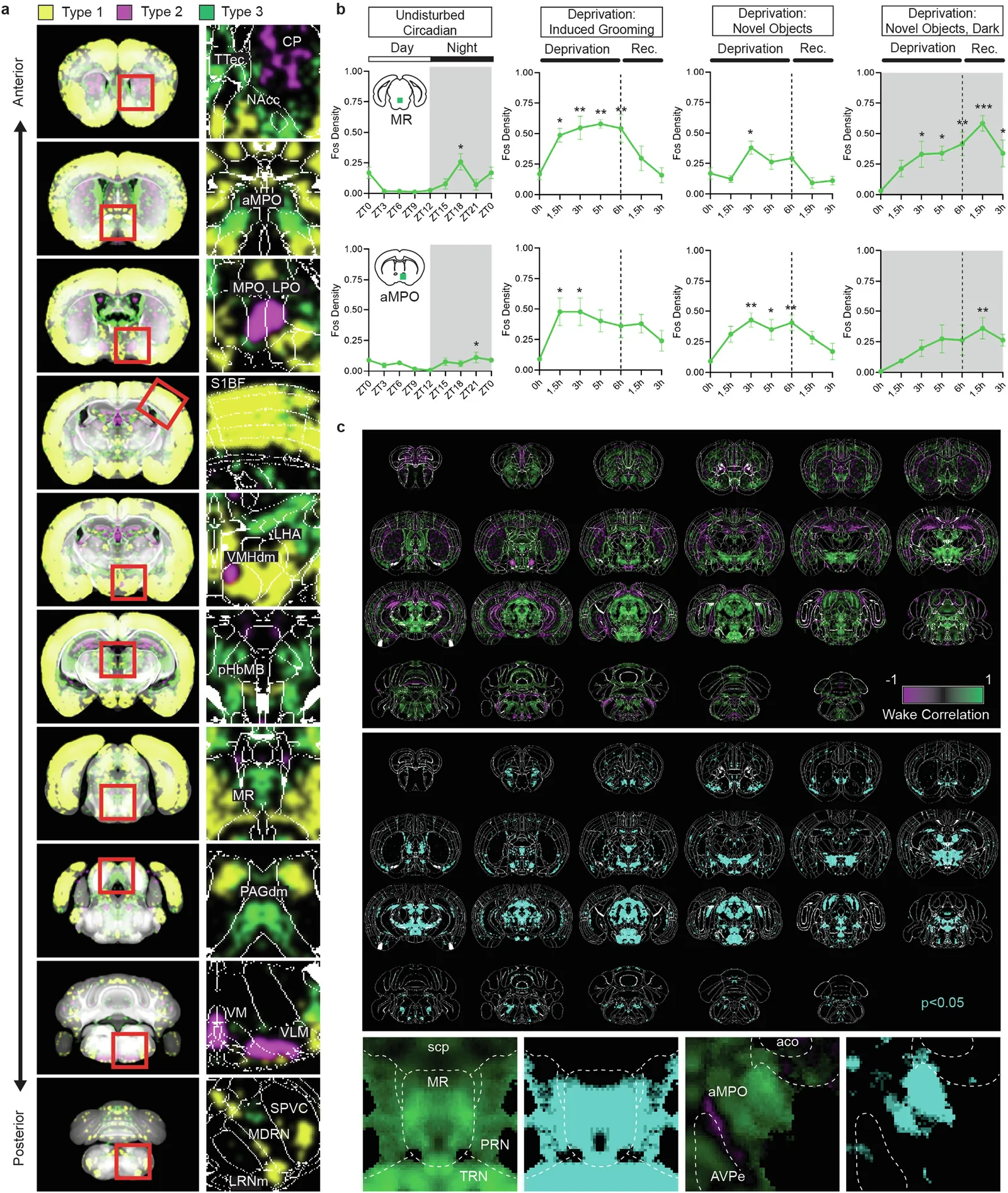
Cluster anatomy and MR and aMPO region responses. **a**, Anatomical distribution of type 1–3 responses common to both induced grooming and novel objects-based sleep deprivation. Example planes are shown in anterior to posterior order. Yellow, type 1; magenta, type 2; green, type 3. **b**, MR (top row) and aMPO (bottom row) FOS responses during circadian and sleep deprivation experiments. Mean ± s.e.m. For MR circadian, *p = 0.0184; for MR induced grooming-based deprivation, *p = 0.0322 (1.5 h), **p = 0.0082 (3 h); **p = 0.0036 (5 h), **p = 0.0090 (6 h); for MR novel objects-based deprivation, *p = 0.0184; for MR dark phase deprivation, *p = 0.0462 (3 h), *p = 0.0390 (5 h), **p = 0.0072 (6 h), ***p = 0.0002 (Recovery 1.5 h), *p = 0.0396 (Recovery 3 h); for aMPO circadian, *p = 0.0245; for aMPO grooming-based deprivation, *p = 0.0249 (1.5–3 h); for aMPO novel objects-based deprivation, **p = 0.0023 (3 h), *p = 0.0288 (5 h), **p = 0.0048 (6 h); for aMPO dark phase deprivation, **p = 0.0082. One-way ANOVA for all comparisons. In circadian experiments, all time points were compared to ZT12. In deprivation experiments, all time points were compared to 0 h. n = 6–7 brains per time point for circadian and light phase deprivation, n = 4 brains per time point for dark phase deprivation. **c**, Top, summary of wake-correlated regions across the brain. Pearson correlations with wake duration were calculated per voxel for a 2 h time window preceding each time point in the undisturbed circadian experiment. Magenta–green colour scale indicates Pearson correlation coefficients between −1 and 1. Middle, voxels with significant correlations. Bottom, higher-magnification views for the MR and aMPO. TTec, taenia taecta; CP, caudoputamen; NAcc, nucleus accumbens; aMPO, anterior medial preoptic area; LPO, lateral preoptic area; MPO, medial preoptic area; S1BF, somatosensory cortex barrel fields; LHA, lateral hypothalamic area; VMHdm, ventromedial hypothalamus, dorsomedial portion; pHbMB, peri-habenular midbrain; MR, median raphe; PAGdm, periaqueductal grey, dorsomedial; VM, ventral medulla; VLM, ventrolateral medulla; NTS, nucleus of the solitary tract. The brain schematics in **b** were adapted from ref. ^76^, Springer Nature Limited, under a Creative Commons licence CC BY 4.0.

Type 1 responses peaked 1.5–3 h into sleep deprivation and decayed thereafter, whereas type 2 responses peaked during the recovery phase. By contrast, type 3 responses exhibited accumulating or sustained patterns during 6-h deprivation and decayed during the ensuing recovery phase (Fig. 1b). We observed the same three response types for both grooming- and novel-object-based deprivation, suggesting similar brain-wide FOS dynamics despite different deprivation stimuli. In anatomical space, type 1 responses accounted for the greatest proportion of the brain and were enriched in cortical areas, whereas type 2 and 3 responses were highly restricted and predominantly subcortical (Fig. 1c,d and Extended Data Fig. 3a).

We hypothesized that brain areas that reflect sleep deprivation and regulate normal sleep behaviour should be (1) shared between both sleep-deprivation methods and (2) more activated during spontaneous or sleep-deprivation-induced wakefulness than during sleep. Indeed, regions within type 3 clusters, such as the median raphe (MR) and the most anterior portion of the medial preoptic area (aMPO), were common to both deprivation methods and exhibited wake-correlated patterns during unperturbed behaviour—their activation increased during the night, when mice are mostly awake, and decayed after light onset, when mice are mainly sleeping (Fig. 1d,e and Extended Data Figs. 1 and 3b,c). The consistent wake-correlated responses during both sleep deprivation and undisturbed sleep–wake behaviour suggest that type 3 regions are not selective for experimentally induced sleep deprivation, but might also reflect natural changes in sleep drive in the absence of experimental stimuli. Type 3 activation patterns also scaled with the duration of wakefulness, reaching approximately threefold higher levels during sleep-deprivation experiments relative to maximal night-time levels in unperturbed mice. Whereas these responses decayed immediately during the recovery period after daytime deprivation, they remained elevated after night-time deprivation, when mice exhibit an additional 2 h latency to sleep^2^ (Fig. 1e and Extended Data Fig. 1j).

In contrast to type 3 activation patterns, regions enriched in type 2 responses, such as the lateral preoptic area (LPO) and ventral medulla (VM), exhibited a large peak in the middle of the light period, when unperturbed mice have been sleeping, and a smaller peak overlapping with the night-time siesta^26^, consistent with the known sleep-active, sleep-promoting neuronal populations in the LPO^18,19,27–31^ and VM^32,33^. Both regions reached peak activation 3 h into recovery after light-period sleep deprivation but not dark-period deprivation, further supporting the idea that type 2 responses include sleep-active brain regions (Fig. 1d and Extended Data Fig. 3a–c). Unlike type 2 or type 3 regions, type 1 regions such as somatosensory, visual and auditory cortices exhibited minimal responses during unperturbed behaviour, instead peaking and decaying early during all deprivation experiments (although remaining elevated relative to baseline; Fig. 1d). Thus, type 1 FOS responses correlate with sensory stimulation, novelty or cognitive engagement rather than wake duration.

In summary, type 3 response patterns are consistent with a system that reflects sleep deficit, and conceptually resemble increasing FOS expression in thirst- or hunger-associated brain regions during water or food deprivation^14,34,35^. Specific type 3 responses were shared across multiple sleep-deprivation methods as well as spontaneous sleep–wake behaviour, and thus highlight candidate areas associated with sleep propensity.

## Deprivation-TRAP cells promote sleep

Because type 3 response profiles could indicate either sleep drive or wake maintenance, we directly tested the functions of candidate regions using genetically targeted manipulations. From regions enriched in type 3 responses, we selected the MR and aMPO for further analysis on the basis of their particularly strong activation during sleep deprivation and their significant correlations with wake duration during unperturbed behaviour (Extended Data Fig. 3b,c). Although previous studies have implicated the MR in sleep regulation, the mechanisms remain unclear. For example, destruction of the raphe system can produce insomnia in cats, whereas electrical stimulation of the MR increases slow-wave activity in rodents, but the cell types that mediate these effects are unknown^36,37^. Broad ablation of raphe serotonergic cells produces contrasting results depending on the targeted populations, decreasing either NREM sleep and delta rebound or rapid eye movement (REM) sleep^38,39^. However, the specific contributions of the MR were not tested. Meanwhile, the preoptic area contains known sleep-active, sleep-promoting cells such as galanin^+^ neurons in the LPO^18,28–31^, but the wake-correlated, deprivation-responsive profile of the aMPO suggests novel cell types with distinct functions (also see Extended Data Fig. 14).

**Fig. 2:**
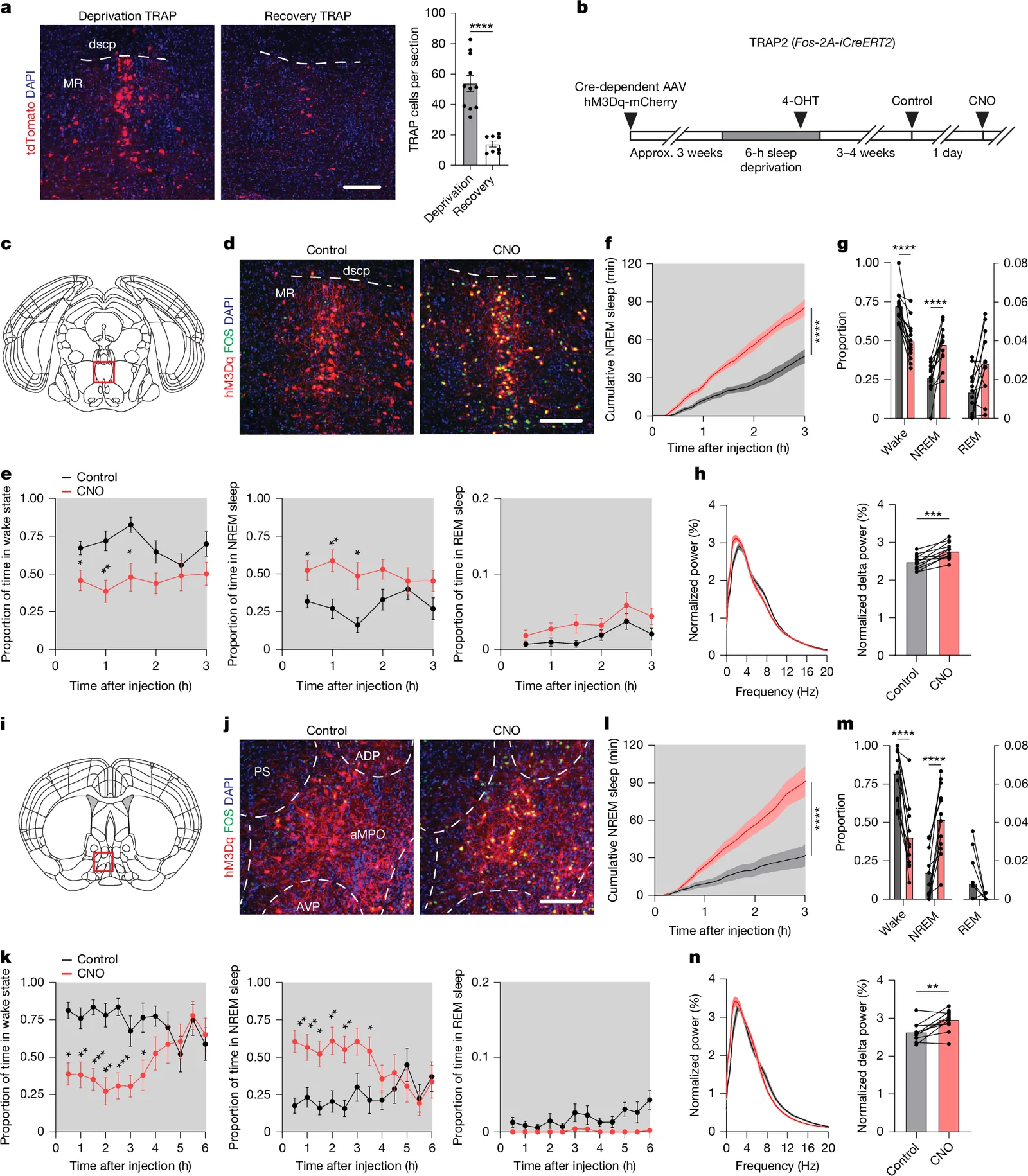
Deprivation-TRAP cells promote NREM sleep and slow-wave activity. **a**, Deprivation-TRAP labels MR cells more efficiently than recovery-TRAP does. Red, tdTomato reporter; blue, DAPI. Right, quantification of MR TRAP cells per section. Deprivation-TRAP, *n* = 7; recovery-TRAP, *n* = 3. ****P < 0.0001, unpaired two-tailed *t*-test. dscp, decussation of the superior cerebellar peduncle. **b**, Strategy to chemogenetically activate deprivation-TRAP cells in either the MR or the aMPO. 4-OHT, 4-hydroxytamoxifen; Approx., approximately; CNO, clozapine *N*-oxide. **c**, Schematic of the MR region shown in **d**. **d**, Expression of the chemogenetic activator hM3Dq-mCherry (red) in MR deprivation-TRAP cells. FOS (green) indicates robust activation after administration of CNO. **e**, Activation of MR deprivation-TRAP cells increases NREM sleep. Proportion of time in wake (left), NREM sleep (middle) and REM sleep (right) during a 3-h window after injection of control (black) or CNO (red) at the beginning of the dark phase (grey shading here and in **f**,**k**,**l**). *P = 0.0321 (0.5 h), **P = 0.0075 (1 h), *P = 0.0401 (1.5 h) for wake; *P = 0.0321 (0.5 h), **P = 0.0083 (1 h), *P = 0.0391 (1.5 h) for NREM sleep; two-way ANOVA. **f**, Cumulative NREM sleep during the 3 h after injection. *n* = 13 mice. ****P < 0.0001, paired two-tailed *t*-test. **g**, Proportion in each state within the same 3-h window. Each data point represents one mouse. *n* = 13. ****P < 0.0001, two-way repeated-measures ANOVA. **h**, Activation of MR deprivation-TRAP cells increases slow-wave activity in the delta range (0.25–4 Hz). NREM power spectra are shown as a percentage of total power (left) and mean delta power (right). ***P = 0.0002, paired two-tailed *t*-test. **i**–**n**, As in **c**–**h**, respectively, but for aMPO deprivation-TRAP cell activation. *n* = 12 mice. For **k**, *P = 0.0117 (0.5 h), **P = 0.0044 (1 h), ***P = 0.0007 (1.5 h), **P < 0.0018 (2 h), ***P = 0.0009 (2.5 h), *P = 0.0458 (3.5 h) for wake; **P = 0.0084 (0.5 h), **P = 0.0089 (1 h), *P = 0.0191 (1.5 h), **P = 0.0073 (2 h), **P = 0.0014 (2.5 h), *P = 0.0491 (3.5 h) for NREM sleep; two-way ANOVA. For **l**, ****P < 0.0001, paired two-tailed *t*-test. For **m**, ****P < 0.0001; two-way repeated-measures ANOVA. For **n** (*n* = 10 mice, because 2 mice did not exhibit NREM sleep in control conditions), **P = 0.0067, paired two-tailed *t*-test. All data are mean ± s.e.m. Scale bars, 200 µm. The brain schematics in **c**,**i** were adapted from ref. ^76^, Springer Nature Limited, under a Creative Commons licence CC BY 4.0. Source data

**Extended Data Fig. 4:**
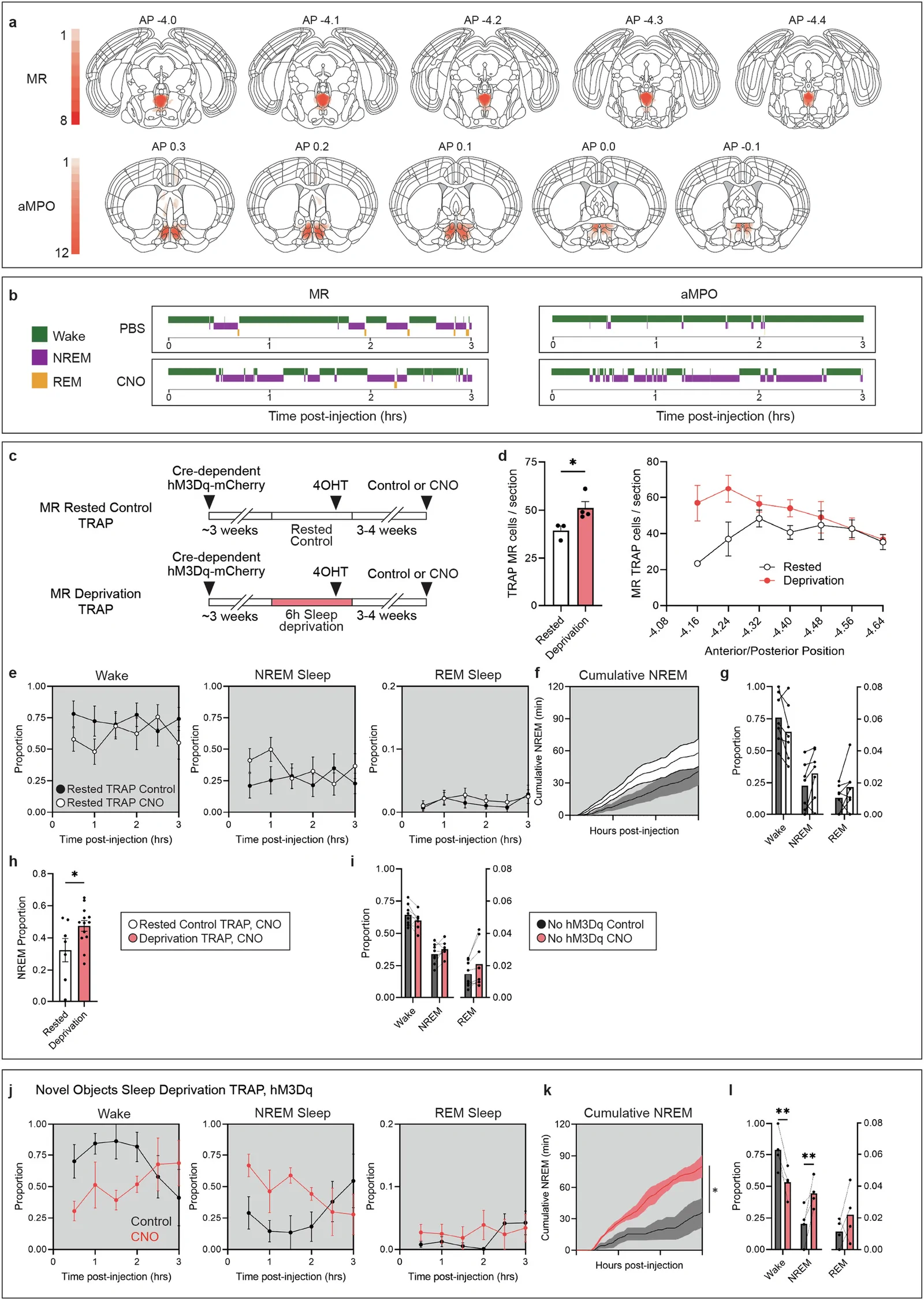
Deprivation-TRAP and control-TRAP anatomical and behavioural analyses. **a**, Heat maps of targeted viral expression in MR or aMPO TRAP experiments, mapped onto coronal brain atlas images. Numbers above each coronal section indicate anteroposterior (AP) distance from bregma. Scale bars indicate number of mice. **b**, Representative within-mouse hypnograms after ZT12 administration of PBS or CNO, for data shown in Fig. 2e,k. Green, wake. Purple, NREM. Yellow, REM. **c**, Schematic of rested control-TRAP and deprivation-TRAP experiments. **d**, Number of control or deprivation-TRAP cells in the median raphe (left), and approximate position of labelled cells relative to bregma according to the Paxinos mouse brain atlas. Control-TRAP n = 3 mice, deprivation-TRAP n = 4 mice. *p = 0.0456, unpaired two-tailed *t*-test. For both groups, TRAP cells were labelled with Cre-dependent EYFP. **e**, Chemogenetic activation of MR control-TRAP cells does not significantly affect sleep. Wake (left), NREM (middle), and REM (right) proportions during a 3 h window after control (black) or CNO (white) injection. **f**, Cumulative NREM sleep during the 3 h after injection. **g**, Proportion in each state within the same 3 h window. **h**, CNO administration increases NREM sleep in deprivation-TRAP (red, n = 13) relative to Rested TRAP (white, n = 7) mice expressing Cre-dependent hM3Dq-mCherry. *p = 0.0435, unpaired two-tailed *t*-test. **i**, Control mice without hM3Dq expression do not exhibit significant changes in sleep/wake duration after 1 mg/kg CNO administration. Grey, control; red, CNO. n = 8 mice. **j**, Chemogenetic activation of novel objects-based sleep deprivation-TRAP cells promotes sleep, similar to grooming-based sleep deprivation-TRAP. For comparison, see Fig. 2e–g. **k**, Cumulative NREM sleep during the 3 h after injection. n = 4 mice. *p = 0.0412, paired two-tailed *t*-test. **l**, Proportion in each state within the same 3 h window. **p = 0.0067 (Wake), **p = 0.0088 (NREM), two-way repeated-measures ANOVA. The brain schematics in **a** were adapted from ref. ^76^, Springer Nature Limited, under a Creative Commons licence CC BY 4.0.

To selectively target MR or aMPO cells that become activated during sleep deprivation, we used a mouse line with inducible Cre recombinase knocked in at the *Fos* locus (TRAP2, *Fos-2A-iCreERT2*)^40^. Cre induction towards the end of a 6-h deprivation period (deprivation-TRAP) labelled threefold more cells relative to Cre induction during recovery, consistent with type 3 FOS patterns (Fig. 2a). We thus delivered Cre-dependent virus encoding the chemogenetic activator hM3Dq specifically to the MR or aMPO of TRAP2 mice, induced Cre recombination during late deprivation and activated cells 3–4 weeks later at the beginning of the dark period, when mice are well-rested (Fig. 2b and Extended Data Fig. 4a). Activation of deprivation-TRAP cells in either the MR or the aMPO increased sleep duration two- to threefold (Fig. 2c–g,i–m and Extended Data Fig. 4b), as classified using EEG recordings. Notably, deprivation-TRAP cells in both regions also increased NREM slow-wave activity in the delta (0.25–4 Hz) range, indicating higher sleep intensity (Fig. 2h,n). Activating deprivation-TRAP cells in the MR or aMPO during the dark phase is thus sufficient to induce NREM sleep that is more characteristic of the light phase, when mice have accumulated greater sleep need. By comparison, rested control-TRAP labelled fewer cells and their activation did not significantly promote sleep (Extended Data Fig. 4c–l), consistent with the idea that sleep-promoting cells in the MR are less active in rested mice but become more activated during prolonged wakefulness.

**Extended Data Fig. 5:**
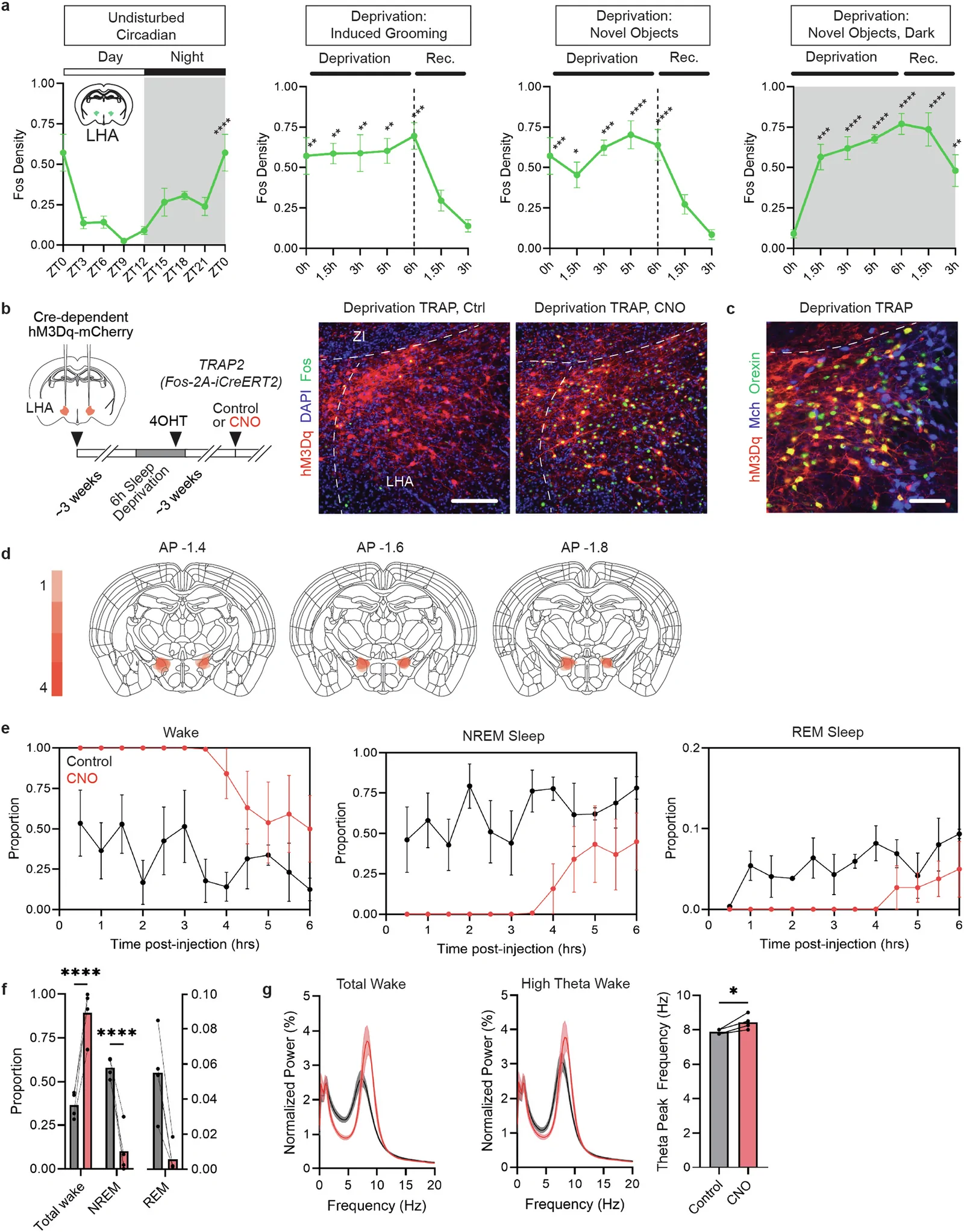
LHA deprivation-TRAP cells promote wakefulness. **a**, Lateral hypothalamic area (LHA) FOS responses during circadian and sleep deprivation experiments. Grey shading indicates dark periods. Mean ± s.e.m. For circadian, ****p < 0.0001; for induced grooming-based deprivation, **p = 0.0048 (0 h), **p = 0.0033 (1.5 h), **p = 0.0031 (3 h), **p = 0.0022 (5 h), ***p = 0.0002 (6 h); for novel objects-based deprivation, ***p = 0.0006 (0 h), *p = 0.0120 (1.5 h), ***p = 0.0001 (3 h), ****p < 0.0001 (5–6 h); for dark phase deprivation, ***p = 0.0002 (1.5 h), ****p < 0.0001 (3h-Recovery 1.5 h), **p = 0.0017 (Recovery 3 h). One-way ANOVA for all comparisons. In circadian experiments, all time points were compared to ZT12. In light phase deprivation experiments, all time points were compared to 3 h recovery, while dark phase deprivation time points were compared to 0 h. n = 6–7 brains per time point for circadian and light phase deprivation, n = 4 brains per time point for dark phase deprivation. **b**, Chemogenetic activator hM3Dq-mCherry expression (red) in LHA deprivation-TRAP cells. FOS (green) indicates robust activation after CNO administration. Scale bar, 200 µm. **c**, LHA deprivation-TRAP cells express orexin/hypocretin (green) but not melanin-concentrating hormone (MCH, blue). Scale bar, 100 µm. **b**,**c**, Representative images selected from n = 4 replicates. **d**, Heat maps of viral expression mapped onto the mouse brain atlas. AP = anteroposterior position relative to bregma in mm. **e**, Wake, NREM and REM proportions during a 6 h window following control (black) or CNO (red) injection at the beginning of the light phase. n = 4 mice. **f**, State proportions during the same 6 h window. n = 4 mice. ****p < 0.0001, Two-way repeated-measures ANOVA. **g**, Power spectra for total wake and high-theta wake. Right panel, high-theta wake peak frequency. n = 4 mice, *p = 0.0374, paired two-tailed *t*-test. **a**,**e**,**g**, Data presented as mean ± s.e.m. The brain schematics in **a**,**b**,**d** were adapted from ref. ^76^, Springer Nature Limited, under a Creative Commons licence CC BY 4.0.

Deprivation-TRAP sleep-promoting effects did not extend to all tested type 3 regions. For example, the lateral hypothalamic area (LHA) also exhibited sustained FOS responses during deprivation, but activating deprivation-TRAP cells in the LHA strongly promoted wakefulness rather than sleep (Extended Data Fig. 5a–f). Indeed, LHA deprivation-TRAP cells expressed orexin rather than melanin-concentrating hormone (MCH; Extended Data Fig. 5c), consistent with the known wake-inducing and stabilizing functions of orexin^+^ neurons^4,7^. Thus, deprivation-responsive type 3 activation signatures encompass cell types with opposing functions, promoting sleep or wakefulness in a region-specific manner.

## Deprivation-TRAP cells in normal sleep behaviour

**Extended Data Fig. 6:**
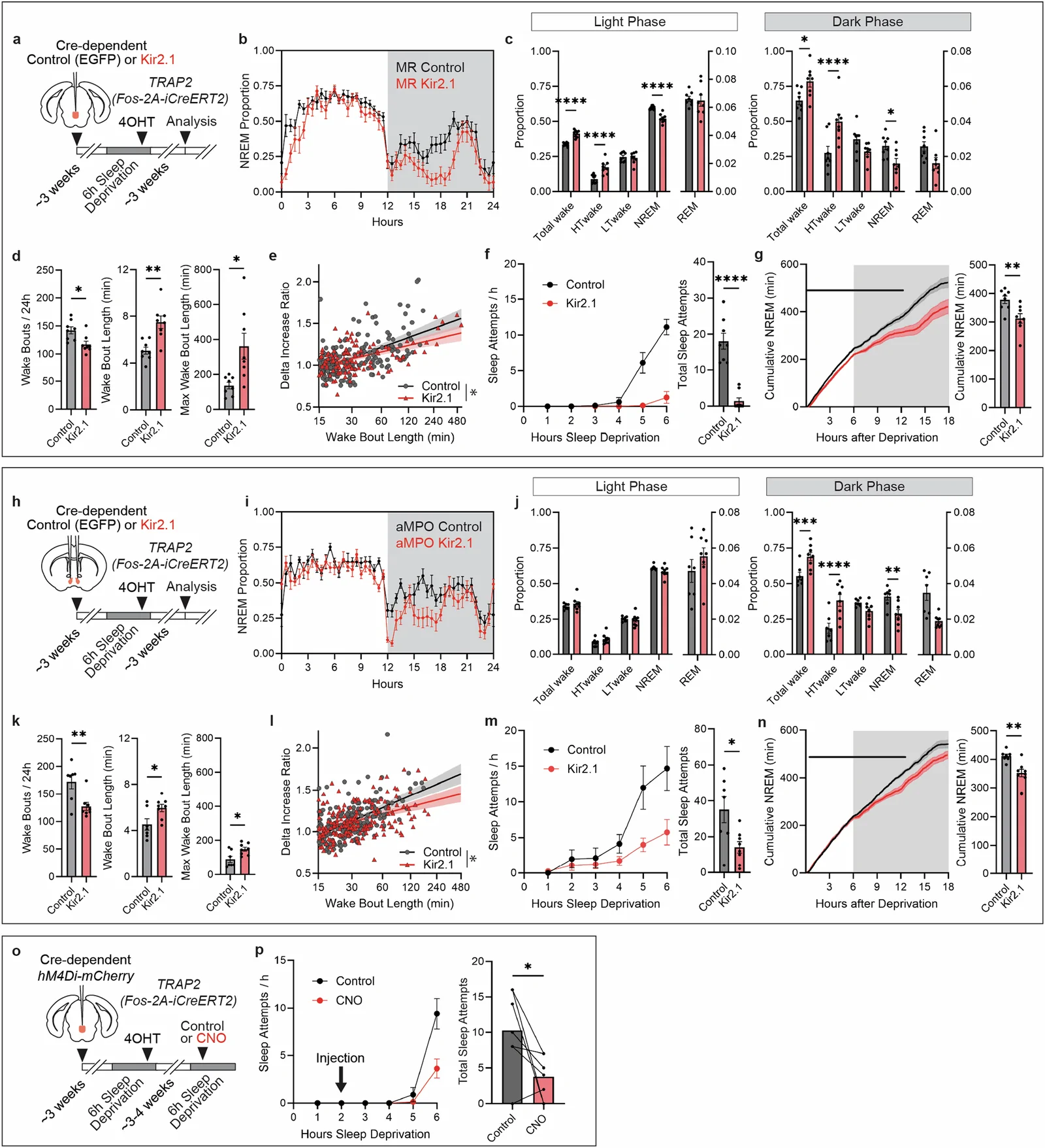
Deprivation-TRAP cell activity is required for normal sleep and deprivation response. **a**, Strategy to inhibit MR deprivation-TRAP cells. **b**, MR deprivation-TRAP cell inhibition decreases NREM sleep. Twenty-four-hour time course of NREM proportion during unperturbed conditions, averaged across 5 days, n = 8 mice per group. Black, Control EGFP. Red, Kir2.1. **c**, State proportions for each mouse, separated by light and dark phases. Grey, Control EGFP. Red, Kir2.1. n = 8 mice per group. Mean ± s.e.m. For light phase, ****p < 0.0001; for dark phase, *p = 0244 (total wake), *p = 0.0452 (NREM), ****p < 0.0001 (HTwake). All comparisons with two-way repeated-measures ANOVA. **d**, Kir2.1 mice exhibit longer wake bouts. Wake-bout number, mean bout length, and maximum bout length for each mouse. *p = 0.0102, **p = 0.0014, *p = 0.0103 respectively, unpaired two-tailed *t*-test. **e**, Kir2.1 mice exhibit decreased delta power accumulation during wake bouts. Y-axis indicates relative delta power (0.25–4 Hz) increases after vs before wake episodes. Error bars indicate 95% confidence intervals. *p = 0.016, β = −0.049, group × wake-bout duration interaction, linear mixed model. **f**, MR deprivation-TRAP Kir2.1 expression nearly abolishes sleep attempts during 6 h sleep deprivation. Left, hourly sleep attempt number during the 6 h deprivation period. Right, total number of sleep attempts. ****p < 0.0001, unpaired two-tailed *t*-test. **g**, Cumulative NREM sleep after the deprivation period. Right panel, minutes NREM sleep during the 12 h interval following deprivation as indicated by the black bar in the left panel. **p = 0.0084, unpaired two-tailed *t*-test. **h**, Strategy to inhibit aMPO deprivation-TRAP cells. **i**–**k**, Twenty-four-hour time course of NREM sleep, state proportions, and wake-bout length for aMPO deprivation-TRAP cell inhibition, presented as in **b**–**d**. Control EGFP n = 7, Kir2.1 n = 8. Mean ± s.e.m, **p = 0.0020, ***p = 0.0005, ****p < 0.0001, two-way repeated-measures ANOVA. For wake-bout analyses, **p = 0.0073, *p = 0.0257, *p = 0.0163 respectively, unpaired two-tailed *t*-test. **l**, aMPO deprivation-TRAP cell inhibition reduces delta power accumulation. *p = 0.001, β = −0.080 group × wake-bout duration interaction, linear mixed model. **m**,**n**, aMPO deprivation-TRAP cell inhibition decreases sleep attempts (*p = 0.0159) (**m**), and inhibited mice sleep less than controls during the recovery period, (**p = 0.0024) (**n**), unpaired two-tailed *t*-test. **o**, Strategy to acutely inhibit deprivation-TRAP cells during sleep deprivation. CNO was injected 2 h into the deprivation period. **p**, Chemogenetic inhibition of MR deprivation-TRAP cells during deprivation decreases sleep attempts. n = 8 mice. *p = 0.0204, unpaired two-tailed *t*-test. Unless stated otherwise, all data are presented as mean ± s.e.m. The brain schematics in **a**,**h**,**o** were adapted from ref. ^76^, Springer Nature Limited, under a Creative Commons licence CC BY 4.0.

To further test how the MR and aMPO regulate sleep, we chronically decreased the excitability of deprivation-TRAP cells in either region by virally targeting the inward-rectifying potassium channel Kir2.1 (ref. ^41^). Kir2.1-expressing mice spent less time in NREM sleep across the 24-h circadian cycle than did control mice, particularly during the dark phase and the beginning of the light phase. They also exhibited more consolidated wake architecture, with fewer and longer wake bouts (Extended Data Fig. 6a–d,h–k). Indeed, Kir2.1 mice exhibited hours-long wake episodes that were rarely observed in controls. We thus hypothesized that inhibiting deprivation-TRAP cells blunts sleep pressure accumulation. To test this idea, we compared relative increases in NREM delta power before and after wake bouts^42^. We found that delta power increased in proportion to the duration of wake episodes in control mice; however, this increase was smaller in Kir2.1 mice than it was in controls (Extended Data Fig. 6e,l), indicating a slower build-up of sleep drive.

**Extended Data Fig. 7:**
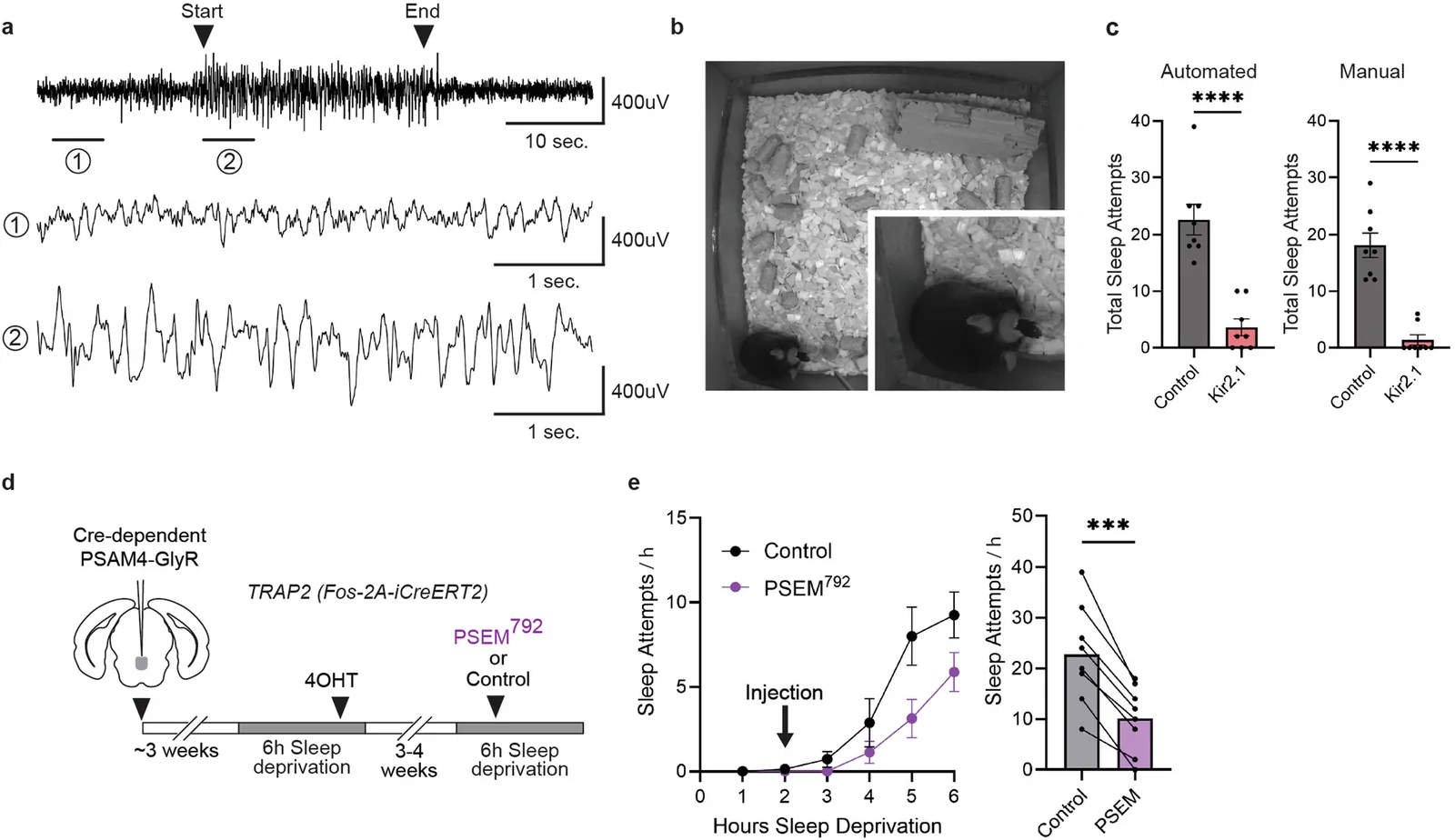
Quantification and chemogenetic inhibition of sleep attempts. **a**, Raw EEG trace with a representative sleep attempt during sleep deprivation. Arrowheads mark approximate beginning and end of sleep attempt. Numbered segments of the trace are shown in the middle and bottom panels. **b**, Mouse in a sleep-like posture during the sleep attempt interval shown above. Inset shows higher magnification. **c**, Total number of sleep attempts during novel objects-based sleep deprivation. Manual and post-hoc automated detection of sleep attempts yields similar results. Mean ± s.e.m. ****p < 0.0001, unpaired two-tailed *t*-test. **d**, MR deprivation-TRAP cells expressing PSAM4-GlyR were inhibited by administering the effector compound PSEM^792^ 2 h into sleep deprivation. **e**, Acutely inhibiting MR deprivation-TRAP cells reduces sleep attempts during sleep deprivation. n = 8 mice. Mean ± s.e.m.***p = 0.0001, paired two-tailed *t*-test. The brain schematic in **d** was adapted from ref. ^76^, Springer Nature Limited, under a Creative Commons licence CC BY 4.0.

On the basis of these observations, we hypothesized that Kir2.1 mice would show lower sleep propensity during sleep deprivation. To test this idea, we quantified the number of sleep attempts per mouse during 6 h of sleep deprivation with novel objects. Sleep attempts were defined as the adoption of an immobile sleep-like posture concurrent with the emergence of NREM slow-wave activity in EEG recordings, and were confirmed post hoc by automated sleep-state classification (Extended Data Fig. 7a–c). Each sleep attempt was terminated by opening the cage door or manually displacing the novel object. Inhibiting deprivation-TRAP cells in the MR or the aMPO strongly reduced the number of sleep attempts in Kir2.1 mice relative to controls, with MR inhibition abolishing sleep attempts completely in some cases (Extended Data Fig. 6f,m). Furthermore, Kir2.1 mice continued to sleep less than control mice during the post-deprivation recovery period (Extended Data Fig. 6g,n). In a complementary approach, we expressed the chemogenetic inhibitors hM4Di or PSAM4-GlyR in MR deprivation-TRAP cells. Acute inhibition 2 h into the sleep-deprivation period reduced sleep attempts in both chemogenetic approaches (Extended Data Figs. 6o,p and 7d,e), suggesting that deprivation-TRAP cell activity is required during wakefulness. Cumulatively, our functional manipulations indicate that MR and aMPO deprivation-TRAP cells are important regulators of sleep drive—activation increases sleep duration and intensity, whereas inhibition decreases baseline sleep levels, allows maintenance of consolidated wake bouts and reduces sleep propensity during deprivation.

## MR deprivation-TRAP cell projections

**Fig. 3:**
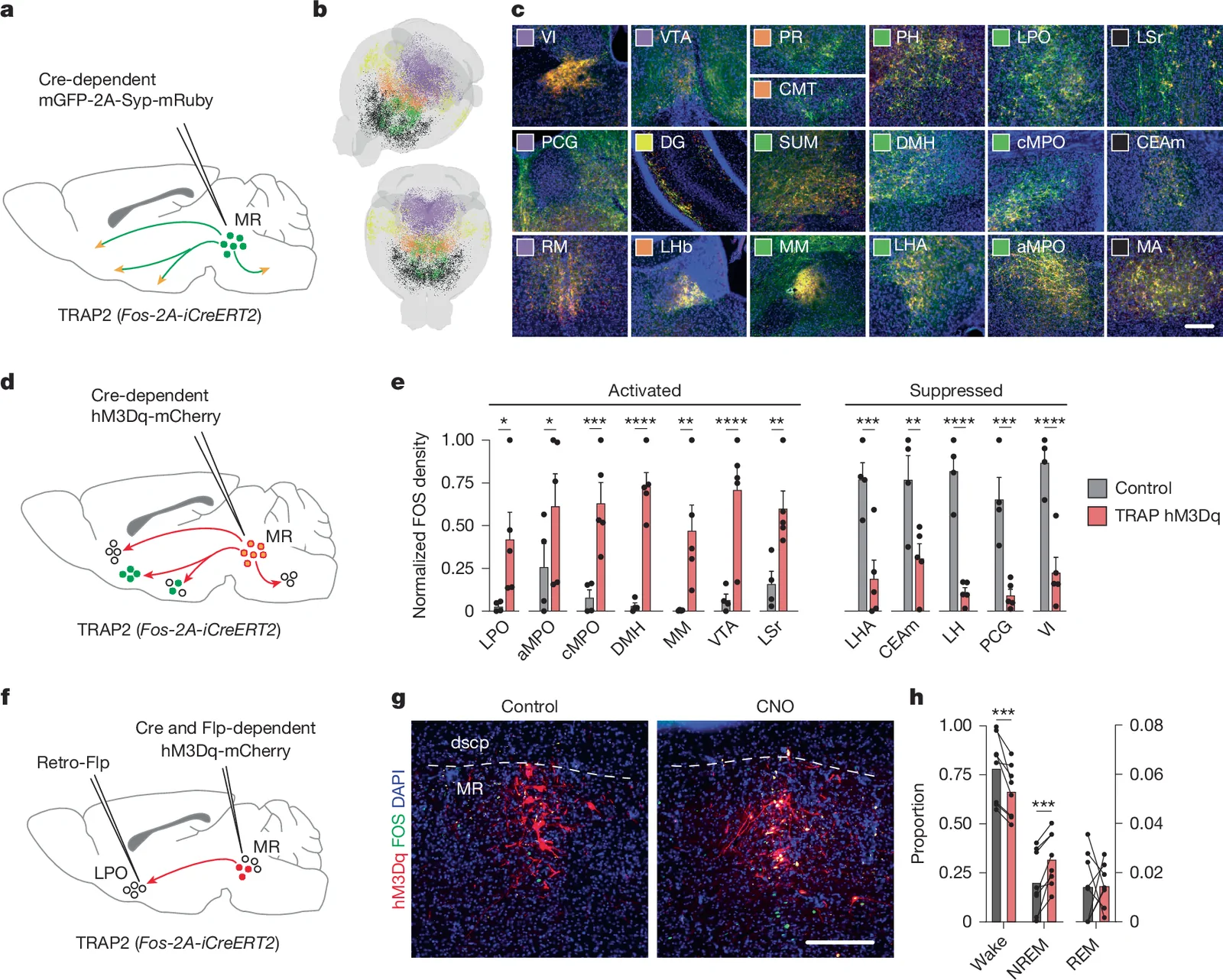
Targets and projection-specific functions of MR deprivation-TRAP cells. **a**, Strategy to label MR deprivation-TRAP cell projections using Cre-dependent mGFP-2A-Synaptophysin-mRuby. **b**, Brain atlas volume with registered Syp-mRuby puncta from a representative mouse, colour-coded by brain area. Magenta, mid and hindbrain; yellow, hippocampus; orange, thalamus; green, hypothalamus; black, striatum and pallidum. **c**, Representative images of target areas receiving mGFP^+^ (green) and mRuby^+^ (red) projections. Blue, DAPI. CEAm, central amygdala, medial; cMPO, commissural medial preoptic area; CMT, centromedial thalamus; DMH, dorsomedial hypothalamus; LHb, lateral habenula; LSr, lateral septum, rostral; MA, magnocellular nucleus; MM, medial mammillary nucleus; PCG, pontine central grey; PH, posterior hypothalamus; PR, peri-reunensis nucleus; RM, raphe magnus; SUM, supramammillary nucleus; VI, abducens nucleus. Scale bar, 200 µm. **d**, Strategy to map FOS responses during chemogenetic activation of MR deprivation-TRAP cells. **e**, FOS responses for regions that receive MR deprivation-TRAP projections and exhibit statistically significant responses. Red, TRAP activation, *n* = 5 mice. Grey, circadian-time-matched no-TRAP control, *n* = 4 mice. *P = 0.0116 (LPO), *P = 0.0220 (aMPO), ***P = 0.0005 (cMPO), **P = 0.0029 (MM), **P = 0.0048 (LSr), ***P = 0003 (LHA), **P = 0.0034 (CEAm), ***P = 0.0004 (PCG), ****P < 0.0001 (all other regions); two-way ANOVA. Data are mean ± s.e.m. **f**, Strategy to chemogenetically activate MR deprivation-TRAP cells that project to the LPO. **g**, Cre and Flp intersectional hM3Dq expression (red) in MR deprivation-TRAP cells, for control (left) and CNO-treated (right) mice. Green, FOS; blue, DAPI. Scale bar, 200 µm. **h**, State proportions during a 3-h window after control (grey) or CNO (red) injection. Activating LPO-projecting cells promotes NREM sleep. *n* = 8 mice. ***P = 0.0002 (wake and NREM); two-way repeated-measures ANOVA. The brain schematics in **a**,**d**,**f** were adapted from the Allen Reference Atlas (https://atlas.brain-map.org/). Source data

Inhibiting deprivation-TRAP cells in the MR nearly abolished sleep attempts during deprivation, suggesting that these neurons have particularly important functions. We thus mapped their projection patterns using virally targeted Cre-dependent mGFP-2A-Synaptophysin-mRuby to simultaneously label axons and putative presynaptic terminals. On the basis of concurrent axon and synaptophysin distribution, MR deprivation-TRAP cells directly targeted subcortical regions broadly distributed across the hindbrain, midbrain, hippocampus, thalamus and hypothalamus, including the aMPO, LPO and LHA (Fig. 3a–c). These targets represent a subset of known outputs from MR serotonergic and non-serotonergic neurons^43,44^. Notably, we did not observe deprivation-TRAP cell projections to the suprachiasmatic nucleus, a master regulator of circadian rhythm^45,46^.

Because deprivation-TRAP cells are likely to include several cell types with potentially heterogeneous effects, we determined their influence on target areas using chemogenetic activation and FOS mapping. Deprivation-TRAP cells exerted bidirectional and region-specific effects (Fig. 3d,e). For example, activating deprivation-TRAP cells in the MR strongly induced FOS in the aMPO and LPO, but suppressed it in the LHA, central amygdala and lateral habenula.

The LPO is known to be crucial for the proper induction and maintenance of sleep^4^, and might mediate the sleep-promoting effects of the MR. To test this hypothesis, we used a retrograde Flp and Cre intersectional strategy to selectively express hM3Dq in MR deprivation-TRAP cells that project to the LPO (Fig. 3f,g). Chemogenetically activating these cells increased NREM sleep, supporting the idea that the MR promotes sleep through LPO projections (Fig. 3h). However, delta power did not increase, indicating that other targets contribute to the full effects of MR activation. Together, these results suggest a circuit mechanism in which MR deprivation-responsive cells increase sleep propensity by simultaneously activating sleep effectors such as the LPO and inhibiting wake effectors such as the LHA.

## GABAergic and serotonergic MR cells promote sleep

**Fig. 4:**
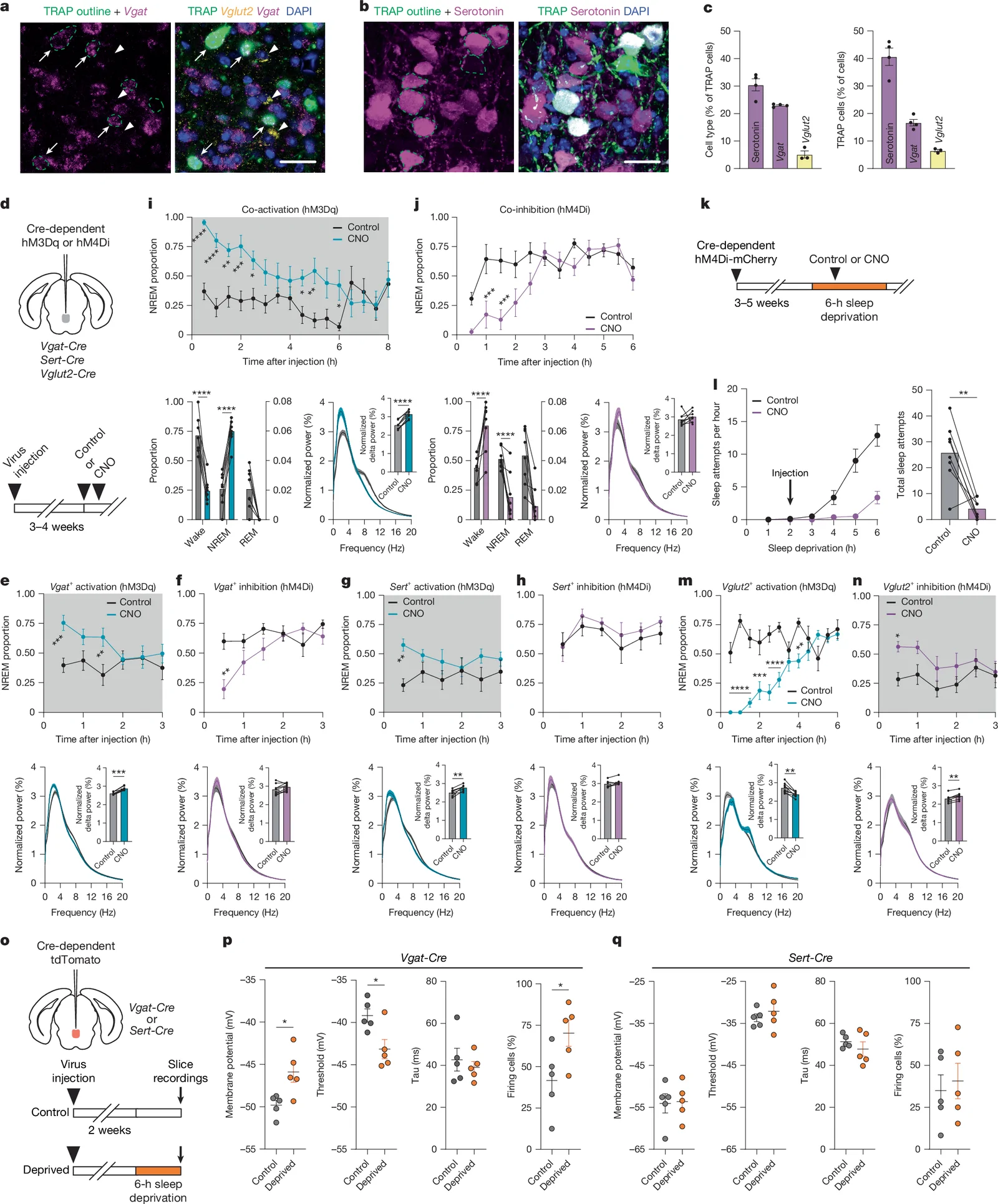
MR GABAergic cells synergistically promote sleep with serotonergic cells, and exhibit increased excitability after sleep deprivation. **a**, YFP + MR deprivation-TRAP cells (green) with fluorescence in situ hybridization for *Vgat* (magenta) and *Vglut2* (yellow). Blue, DAPI. Left image shows dotted outlines of TRAP cells. Arrows mark *Vgat*^+^ TRAP cells. Arrowheads mark *Vglut2*^+^ cells. **b**, Co-localization of TRAP (green) with serotonin antibody staining (magenta). Scale bars, 20 µm (**a**,**b**). **c**, Left, percentage of TRAP cells that express serotonin, *Vgat* or *Vglut2*. Right, percentage of MR serotonin^+^, *Vgat*^+^ and *Vglut2*^+^ cells that are labelled by TRAP. *n* = 3 mice. **d**, Strategy to chemogenetically activate or inhibit specific MR cell types. Cre-dependent hM3Dq-mCherry (activation) or hM4Di-mCherry (inhibition) was virally expressed in the MR in cell-type-specific Cre lines. **e**, *Vgat*^+^ cell activation increases NREM sleep (top) and delta power (bottom). Grey background indicates manipulation at beginning of dark phase here and in **g**,**i**,**n**. *n* = 8 mice. ***P = 0.0007 (0.5 h), **P = 0.0027 (1.5 h); two-way ANOVA. For delta power inset, ***P = 0.0001; paired two-tailed *t*-test. **f**, *Vgat*^+^ cell inhibition decreases NREM sleep. *n* = 9 mice. **P = 0.0015; two-way ANOVA. **g**, *Sert*^+^ cell activation increases NREM sleep (top) and delta power (bottom) at the beginning of the dark phase. *n* = 8 mice. **P = 0.0083; two-way ANOVA. For delta power inset, **P = 0.0015; paired two-tailed *t*-test. **h**, *Sert*^+^ cell activity is not required for NREM sleep during the light phase. *n* = 6 mice. **i**, Chemogenetic co-activation of *Sert*^+^ cells and *Vgat*^+^ cells potently increases NREM sleep (top and bottom left) and NREM delta power (bottom right). *n* = 8 mice. Top, ****P < 0.0001 (0.5–1 h), **P = 0.0038 (1.5 h), ***P = 0.0004 (2 h), *P = 0.0316 (2.5 h), *P = 0.0448 (4.5 h), **P = 0.0018 (5 h), *P = 0.0146 (6 h); two-way ANOVA. Bottom left, ****P < 0.0001; two-way repeated-measures ANOVA. For delta power inset, ****P < 0.0001, paired two-tailed *t*-test. **j**, Chemogenetic co-inhibition of *Sert*^+^ cells and *Vgat*^+^ cells decreases NREM sleep at the beginning of the light phase. *n* = 8 mice. Top ***P = 0.0008 (1 h), ***P = 0.0003 (1.5 h); two-way ANOVA. Bottom left, ****P < 0.0001; two-way repeated-measures ANOVA. **k**, Strategy to inhibit *Sert*^+^ cells and *Vgat*^+^ cells during sleep deprivation. CNO was injected 2 h into the 6-h deprivation period. **l**, Inhibiting *Sert*^+^ and *Vgat*+ cells reduces sleep attempts during deprivation. **P = 0.0017; paired two-tailed *t*-test. **m**, *Vglut2*^+^ cell activation strongly reduces NREM sleep and delta power during the light phase. *n* = 11 mice. ****P < 0.0001 (0.5–1.5 h and 2.5–3 h), ***P = 0.0003 (2 h), **P < 0.0076 (4 h); two-way ANOVA. For delta power inset, *n* = 8 mice, because 3 mice did not exhibit NREM sleep in the CNO condition. **P = 0.0025; paired two-tailed *t*-test. **n**, *Vglut2*^+^ cell inhibition increases NREM sleep in the dark phase. *n* = 8 mice. *P = 0.0178, two-way ANOVA. For delta power inset, *n* = 7 mice, because 1 mouse did not exhibit NREM sleep in the control condition. **P = 0.0020; paired two-tailed *t*-test. **o**, Strategy to examine neuronal intrinsic properties in rested versus sleep-deprived mice. *Vgat*^+^ or *Sert*^+^ cells were virally labelled with Cre-dependent tdTomato, and acute slices were prepared from rested or 6-h sleep-deprived mice for whole-cell recordings. **p**, Sleep deprivation depolarizes the resting membrane potential and reduces the firing threshold in *Vgat*^+^ cells. The membrane time constant (Tau) is unaffected. Far right, in sleep-deprived mice, a greater proportion of *Vgat*^+^ cells spontaneously fire action potentials. Plots show mouse averages (see also Extended Data Fig. 12). Control, *n* = 5 mice, 48 cells; deprived *n* = 5 mice, 46 cells. From left to right, *P = 0.0184, *P = 0.0211, *P = 0.0447; unpaired two-tailed *t*-test. **q**, *Sert*^+^ cell membrane potential, firing threshold, time constant and fraction of spontaneously firing cells. Control, *n* = 5 mice, 60 cells; deprived, *n* = 5 mice, 48 cells. All data are mean ± s.e.m. The brain schematics in **d**,**o** were adapted from ref. ^76^, Springer Nature Limited, under a Creative Commons licence CC BY 4.0. Source data

We next sought to identify deprivation-TRAP cell types in the MR. The MR includes three non-overlapping cell types with distinct neurotransmitter identities. GABAergic *Vgat*-expressing (*Vgat*^+^) cells represent 60% of MR neurons, whereas glutamatergic *Vglut2*^+^ cells and serotonergic cells comprise approximately 25% and 10%, respectively (*Vgat* and *Vglut2* are also known as *Slc32a1* and *Slc17a6*, respectively)^47,48^. Co-localization experiments revealed that MR deprivation-TRAP cells were predominantly *Vgat*^+^ or serotonin^+^, with TRAP cells representing around 40% of all serotonergic cells and around 20% of all *Vgat*^+^ cells (Fig. 4a–c).

**Extended Data Fig. 8:**
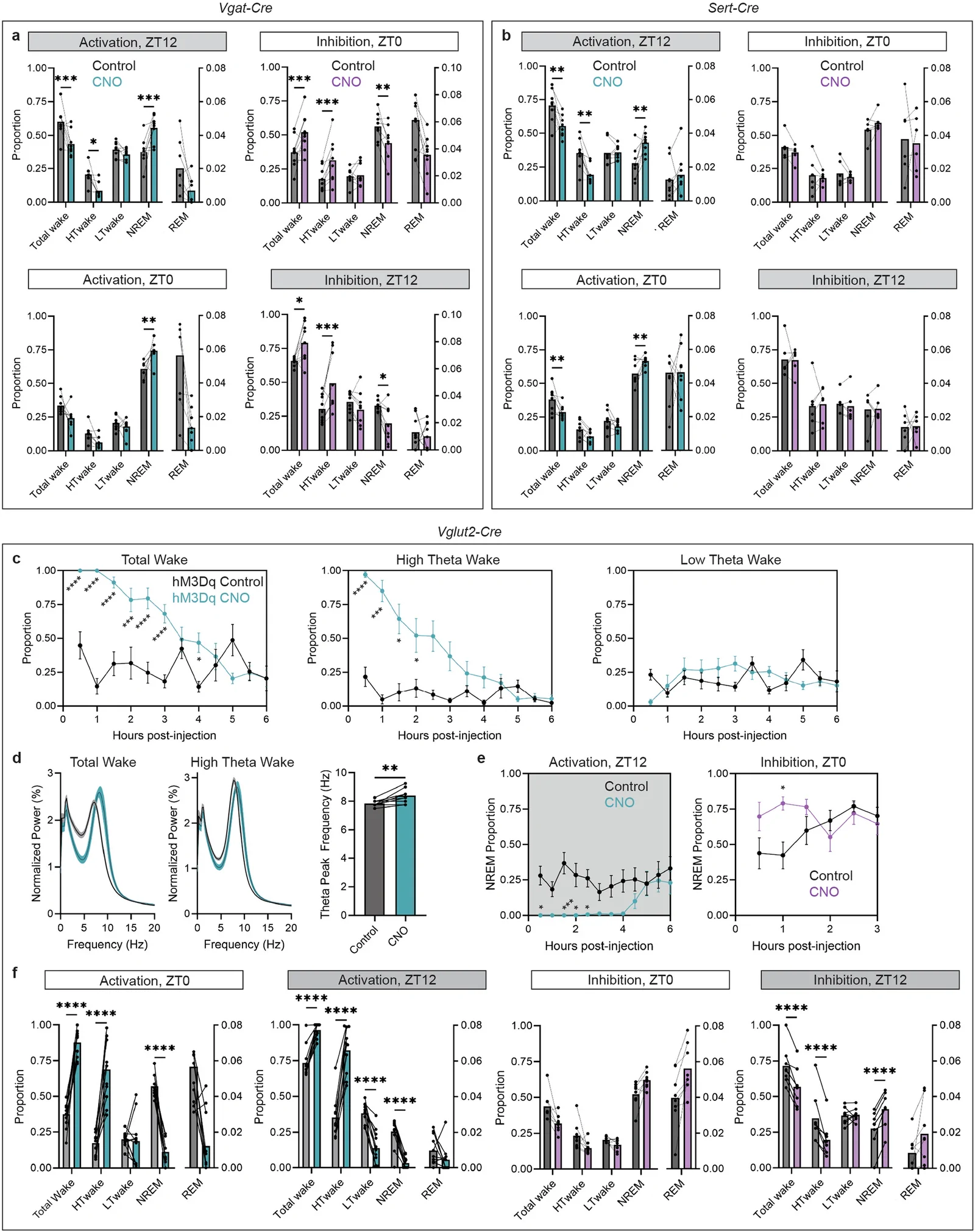
MR cell-type-specific chemogenetic manipulations. Cre-dependent hM3Dq-mCherry (activation) or hM4Di-mCherry (inhibition) was virally expressed in the MR in cell-type-specific Cre lines. Chemogenetic manipulations commenced at the beginning of the light phase (ZT0) or dark phase (ZT12). Grey, Control. Blue, Activation. Magenta, Inhibition, throughout figure. **a**, State proportions for a 3 h time window following *Vgat*+ cell chemogenetic activation or inhibition. Activation n = 8 mice, Inhibition n = 9 mice. For ZT12 activation, ***p = 0.0006 (total wake), *p = 0.0158 (HTwake), ***p = 0.0002 (NREM); for ZT0 activation, **p = 0.0022; for ZT0 inhibition, ***p = 0.0004 (total wake), ***p = 0.0009 (HTwake), **p = 0.0034 (NREM); for ZT12 inhibition, *p = 0.0128 (total wake), ***p = 0.0002 (HTwake), *p = 0.0145 (NREM). Two-way repeated-measures ANOVA for all comparisons. **b**, State proportions in a 3 h time window following *Sert*+ cell chemogenetic activation or inhibition. Activation n = 8 mice, Inhibition n = 6 mice. For ZT12 activation, **p = 0.0022 (total wake), **p = 0.014 (HTwake), **p = 0.0027 (NREM); for ZT0 activation, **p = 0.0017 (total wake), **p = 0.0018 (NREM). Two-way repeated-measures ANOVA for all comparisons. **c**, Total wake, high-theta wake and low-theta wake proportion in a 6 h time window following *Vglut2*+ cell activation. 0 = beginning of light phase. For total wake, *p = 0.0342 (4 h), ***p = 0.0003 (2 h), ****p < 0.0001 (other time points); for high-theta wake, ****p < 0.0001 (0.5 h), ***p = 0.0002 (1 h), *p = 0.0175 (1.5 h), *p = 0.0162 (2 h). Two-way repeated-measures ANOVA for all comparisons. **d**, Total wake and high-theta wake power spectra. *Vglut2*+ cell activation increases high-theta wake peak frequency (right). n = 8 mice. **p = 0.0011, Paired two-tailed *t*-test. **e**, Proportion of time in NREM sleep for *Vglut2*+ cell chemogenic manipulations. Compare to manipulations at reciprocal zeitgeber times in Fig. 4m,n. For ZT12 activation, *p = 0.0120 (0.5 h), ***p = 0.0003 (1.5 h). *p = 0.0102 (2 h), *p = 0.0321 (2.5 h); for ZT0 inhibition, *p = 0.0190. Two-way repeated-measures ANOVA for all comparisons. **c**–**e**, All data are presented as mean ± s.e.m. **f**, State proportions for a 3 h time window after *Vglut2*+ cell chemogenetic activation or inhibition. Activation n = 11 mice, inhibition n = 8 mice. ****p < 0.0001, two-way repeated-measures ANOVA.

To test the behavioural contributions of these cell types, we used corresponding *Vgat* and *Sert* (serotonin transporter) Cre lines for virally targeted chemogenetic activation and inhibition experiments. Activation of either *Vgat*^+^ or *Sert*^+^ cells at the beginning of the night phase increased NREM sleep and delta power above control levels. Conversely, inhibition of *Vgat*^+^ (but not *Sert*^+^) cells decreased NREM sleep at the beginning of the light phase (Fig. 4d–h and Extended Data Fig. 8a,b). Notably, co-activation of both populations markedly increased NREM sleep, for a longer duration than that seen for activation of either cell type alone, and increased delta power more robustly (Fig. 4i). Co-activation thus induced intense NREM sleep that spectrally resembles post-deprivation recovery sleep (Extended Data Fig. 1). Conversely, co-inhibition at the beginning of the light phase decreased NREM sleep (Fig. 4j), and co-inhibition during sleep deprivation nearly eliminated sleep attempts (Fig. 4k,l). These results show that MR GABAergic and serotonergic neurons can modulate sleep drive powerfully during both unperturbed conditions and sleep deprivation.

To determine whether sleep-promoting functions extend to other MR cell types, we targeted *Vglut2*^+^ cells, which represented only a small fraction of deprivation-TRAP cells (Fig. 4c). In contrast to *Sert*^+^ or *Vgat*^+^ cells, activation of *Vglut2*^+^ cells potently promoted wakefulness at the expense of NREM sleep, whereas inhibition produced the opposite effect (Fig. 4m,n and Extended Data Fig. 8c). In line with their wake-promoting function, *Vglut2*^+^ cells increased peak theta frequencies during wakefulness and suppressed delta power during NREM sleep (Fig. 4m and Extended Data Fig. 8c–f). The MR thus promotes or suppresses sleep in a cell-type-specific manner, with a functional dichotomy between GABAergic and serotonergic cells versus glutamatergic cells.

**Extended Data Fig. 9:**
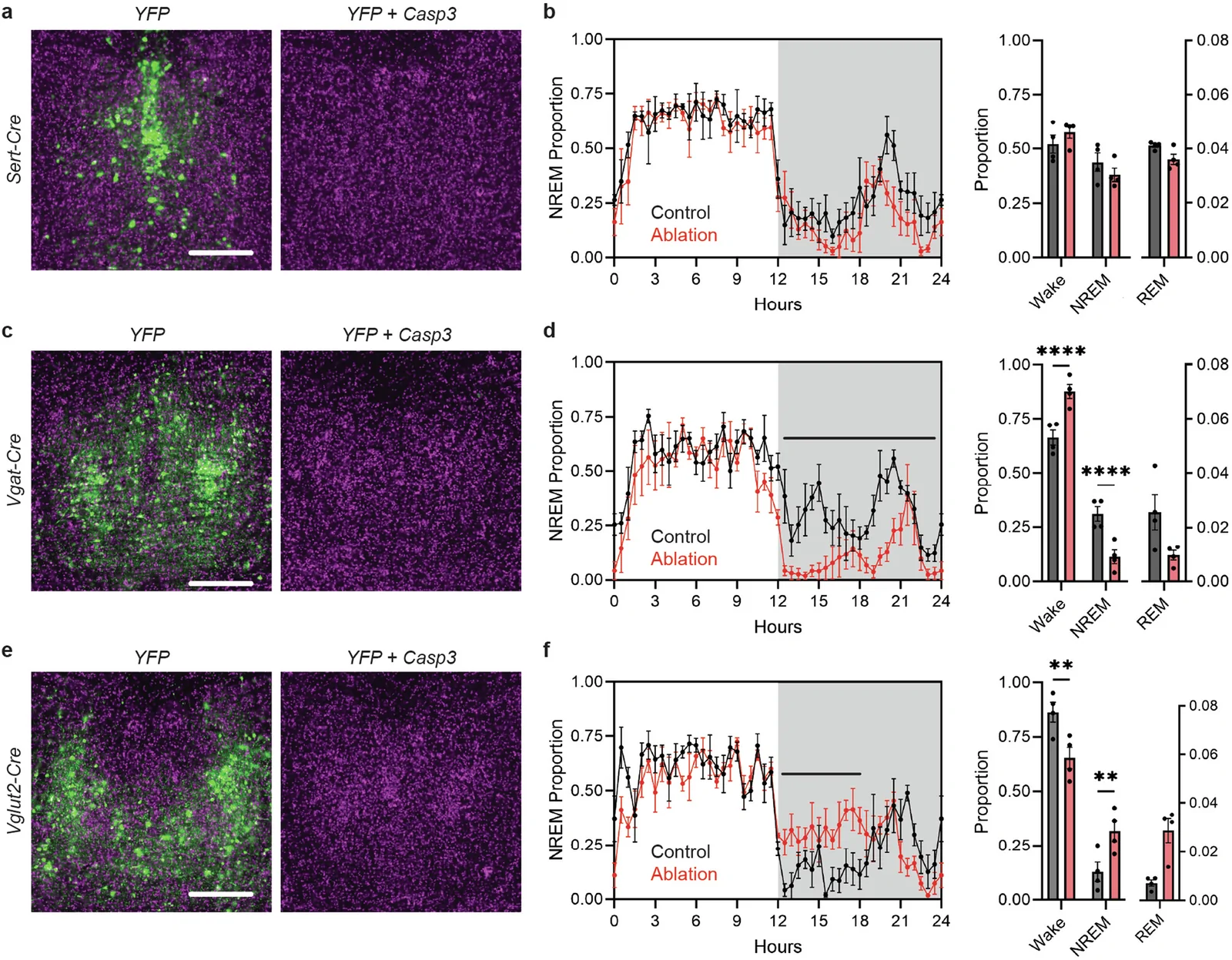
MR cell-type-specific ablation. **a**, Caspase3 (Casp3) expression efficiently ablates MR *Sert*+ cells. Green, YFP. Magenta, DAPI, throughout figure. Scale bar, 250 µm. **b**, Twenty-four-hour NREM proportion, averaged across 5 days. Right, 24-h state proportions. Control n = 4, Ablation n = 4. **c**, Casp3 expression efficiently ablates MR *Vgat*+ cells. **d**, Twenty-four-hour NREM proportion, averaged across 5 days. Right, state proportions for night phase (time interval indicated by black bar in left panel). Control n = 4, Ablation n = 4. ****p < 0.0001, two-way repeated-measures ANOVA. **e**, Casp3 expression efficiently ablates MR *Vglut2*+ cells. **f**, Twenty-four-hour NREM proportion, averaged across 5 days. Right, state proportions for first half of night phase (interval indicated by black bar in left panel). Control n = 4, Ablation n = 4. **p = 0.0010 (wake), **p = 0.0025 (NREM), two-way repeated-measures ANOVA. All data presented as mean ± s.e.m.

Cell-type-specific ablation experiments corroborated our chemogenetic manipulations. Ablation of *Vgat*^+^ cells or *Vglut2*^+^ cells respectively reduced or increased NREM sleep, particularly during the night phase (Extended Data Fig. 9a–f). Together with our TRAP experiments, our histological and functional analyses indicate that *Vgat*^+^ cells and *Sert*^+^ cells become more activated during wakefulness and ultimately promote sleep, whereas *Vglut2*^+^ cells drive wakefulness.

## Lack of sleep boosts MR GABAergic cell excitability

**Fig. 5:**
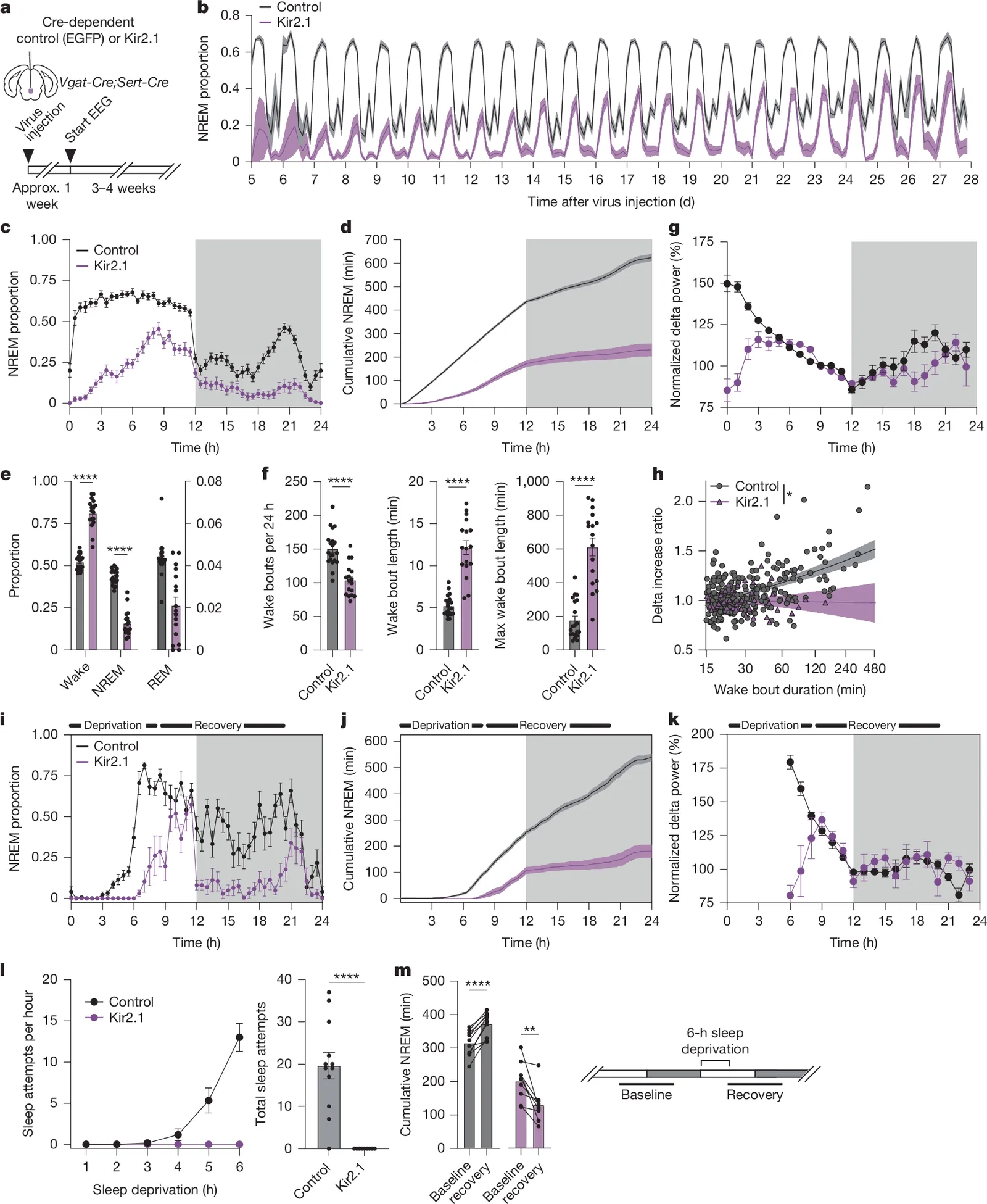
Inhibition of MR GABAergic and and serotonergic cells stably reduces sleep drive. **a**, Strategy to chronically inhibit MR *Sert*^+^ cells and *Vgat*^+^ cells. **b**, NREM sleep proportion during an EEG recording period 5–28 days after virus injection. Control EGFP, grey, *n* = 20; Kir2.1, magenta, *n* = 17. **c**,**d**, Twenty-four-hour time course of NREM sleep proportion (**c**) and cumulative NREM sleep time (**d**), averaged over a 5-day period starting 3–4 weeks after virus injection. **e**, State proportions for each mouse. ****P < 0.0001; two-way ANOVA. **f**, Number of wake bouts, mean length and maximum length. ****P < 0.0001; unpaired two-tailed *t*-test. **g**, Twenty-four-hour time course of delta power (0.25–4 Hz) as a percentage of baseline. **h**, Chronic co-inhibition decreases the rate of delta accumulation. The *y* axis indicates the relative increase in delta power after versus before wake bouts. Error bars, 95% confidence intervals. *P = 0.0130, *β* = −0.120, group × wake-bout duration interaction; linear mixed model. **i**–**k**, NREM sleep proportion (**i**), cumulative NREM (**j**) and NREM delta power (**k**) during 6 h of sleep deprivation and the following recovery period. **l**, Chronic co-inhibition abolishes sleep attempts during the deprivation period. Control EGFP, grey, *n* = 11; Kir2.1, magenta, *n* = 9. ****P < 0.0001; unpaired two-tailed *t*-test. **m**, Co-inhibition abolishes the relative increase in NREM sleep during the 12 h after sleep deprivation versus the same 12 h in undisturbed conditions. Control, ****P < 0.0001; Kir2.1, **P = 0.0059; paired two-tailed *t*-test. Data are mean ± s.e.m. unless otherwise stated. The brain schematic in **a** was adapted from ref. ^76^, Springer Nature Limited, under a Creative Commons licence CC BY 4.0. Source data

**Extended Data Fig. 10:**
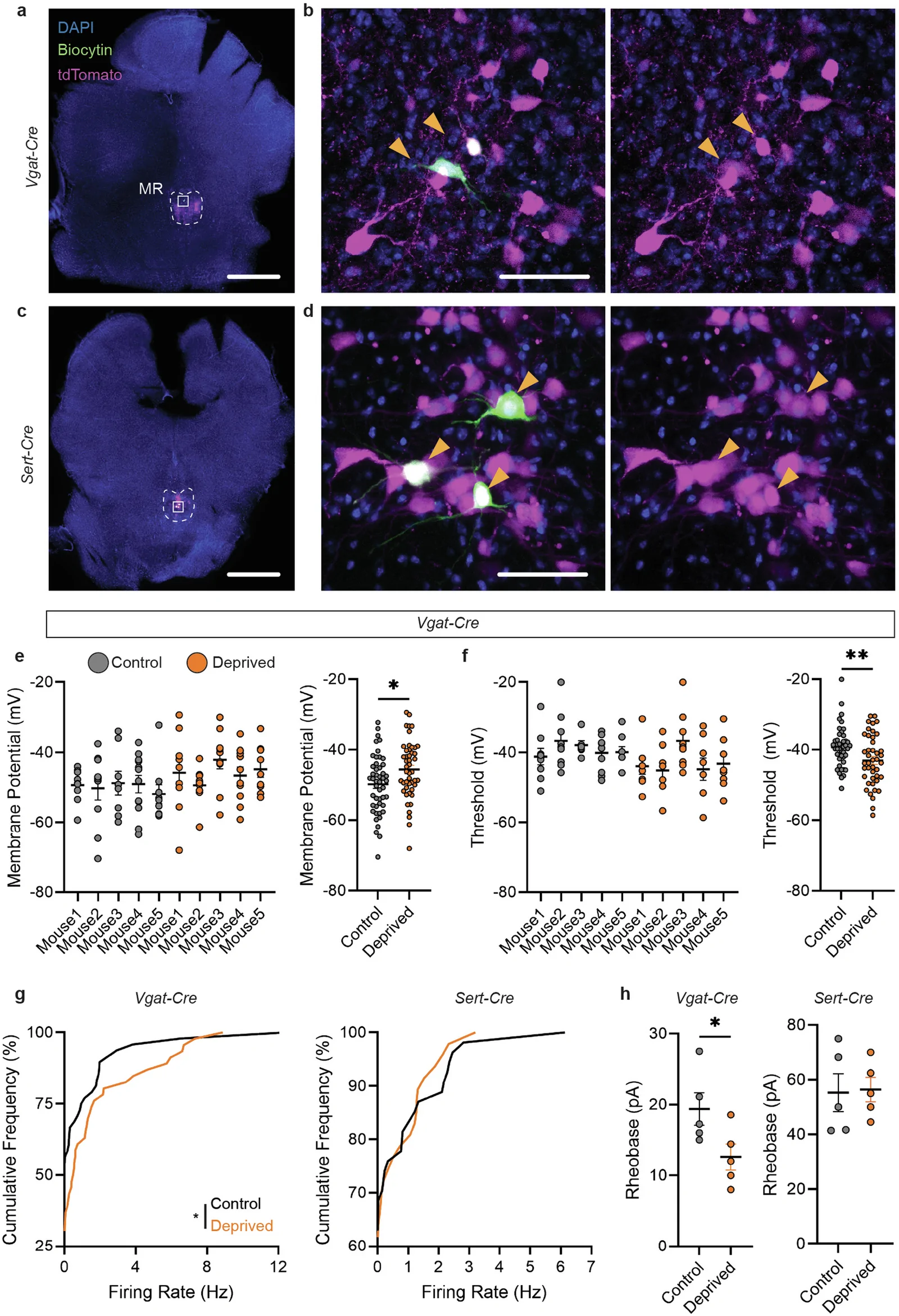
MR slice physiology. **a**, Representative *Vgat-Cre* brain section. Dotted lines indicate the median raphe (MR). Cells were targeted for recordings using Cre-dependent tdTomato expression (magenta), and recorded cells were filled with biocytin (green) for post-hoc confirmation. Scale bar, 1 mm. **b**, Higher-magnification images of the boxed region in a. Arrowheads indicate recorded tdTomato and biocytin double-positive cells. Scale bar, 50 μm. **c**,**d**, Representative brain section (**c**) and recorded *Sert*+ cells (**d**). **a**–**d**, Representative images selected from n = 10 mice each for *Vgat-Cre* and *Sert-Cre*. **e**,**f**, Membrane potential (**e**) and firing threshold (**f**) for all *Vgat*+ cells, segregated by mouse. Right, membrane potential and action-potential firing threshold for all cells. Control n = 5 mice, 48 cells (grey). Deprived n = 5 mice, 46 cells (orange). Mean ± s.e.m. *p = 0.0159, **p = 0.0080, unpaired two-tailed *t*-test. **g**, Cumulative distribution of spontaneous action-potential firing rates for *Vgat*+ and *Sert*+ cells. Sleep deprivation increases *Vgat*+ cell firing rates. *p = 0.0357, Mann Whitney U test. **h**, Sleep deprivation lowers rheobase in *Vgat*+ cells. Control n = 5 mice, deprived n = 5 mice, mean ± s.e.m. *p = 0.0494, unpaired two-tailed *t*-test.

To examine how sleep deprivation leads to the activation of MR GABAergic and serotonergic cells, we compared the intrinsic properties of both cell types in rested and sleep-deprived mice using whole-cell patch clamp recordings (Fig. 4o and Extended Data Fig. 10a–d). Notably, sleep deprivation depolarized the baseline membrane potential of GABAergic cells and reduced their firing thresholds, whereas membrane time constants remained unaltered (Fig. 5p and Extended Data Fig. 10e,f). Consistent with these changes, sleep-deprived mice had a greater proportion of GABAergic cells that spontaneously fired action potentials, and action-potential firing rates were higher than in rested control mice (Fig. 4p and Extended Data Fig. 10g). Furthermore, rheobase was also lower in sleep-deprived mice (Extended Data Fig. 10h). By contrast, serotonergic neurons showed no significant changes in resting membrane potential or firing threshold (Fig. 4q). Sleep deprivation thus specifically shifts the intrinsic properties of MR GABA neurons towards a more excitable state.

## Co-inhibition persistently changes sleep drive

**Extended Data Fig. 11:**
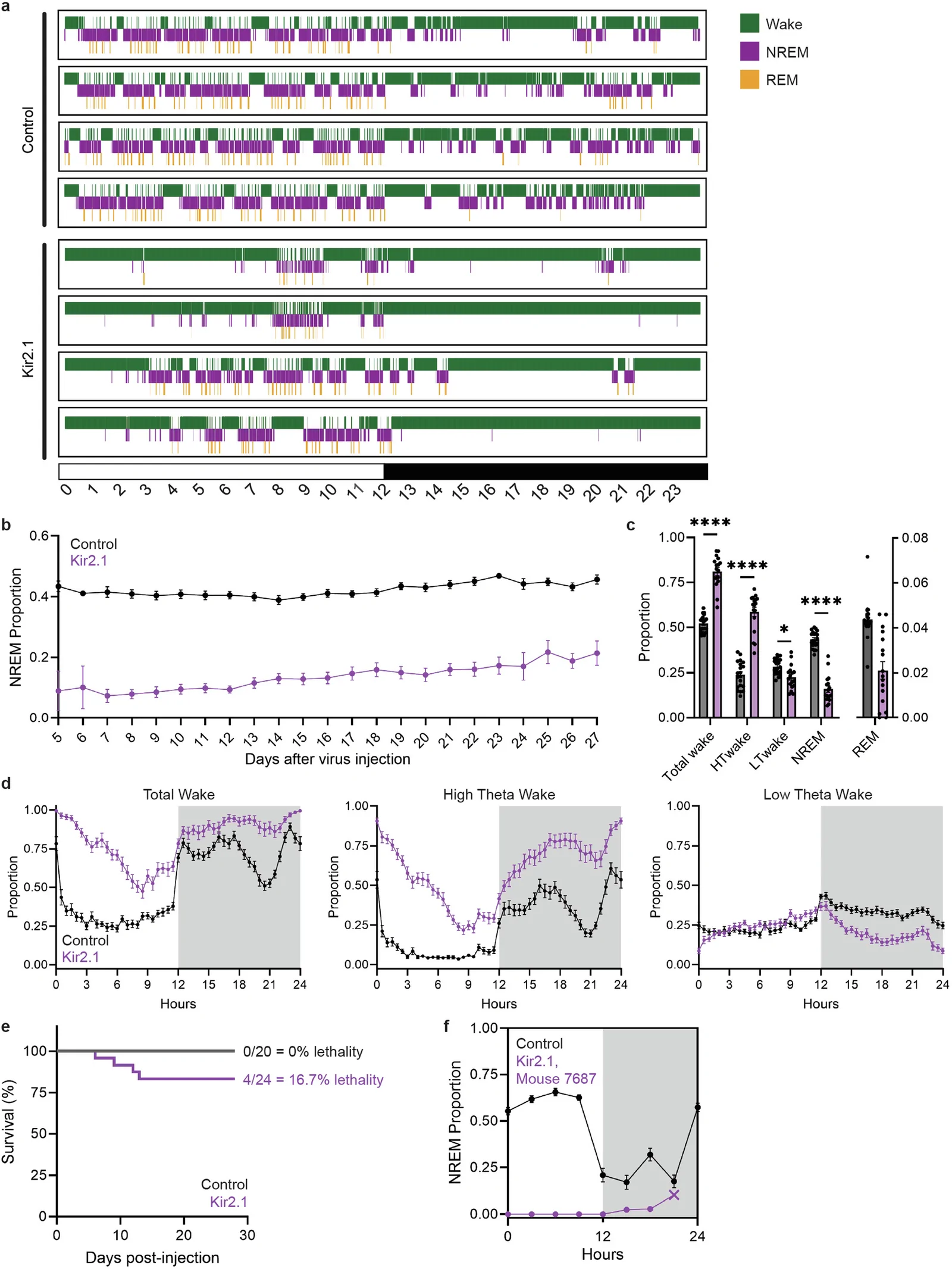
Chronic inhibition of MR GABAergic and serotonergic cells. **a**, Twenty-four-hour hypnograms for four representative control or Kir2.1 mice from the 5-day baseline period shown in Fig. 5c. Green, wake. Magenta, NREM. Yellow, REM. **b**, Daily NREM sleep proportion, based on EEG recording between 5 and 27 days after virus injection in *Sert-Cre*; *Vgat-Cre* mice. Black, Control EGFP. Red, Kir2.1. **c**, Proportion of time spent in each behavioural state for a 5-day period at the end of the recording period. Total wake is subdivided into either high-theta wake (HTwake) or low-theta wake (LTwake). *p = 0.0239, ****p < 0.0001, two-way repeated-measures ANOVA. **d**, Twenty-four-hour time courses of total wake, high theta wake, and low-theta wake. **b**–**d**, Control n = 20, Kir2.1 n = 17. Data presented as mean ± s.e.m. **e**, Survival of control and Kir2.1 mice during 28 days after virus injection. The Kir2.1 manipulation was lethal in 16.7% of cases. **f**, Example Kir2.1 mouse with EEG recording captured before lethality, exhibiting a near-complete absence of sleep. X marks point of death. Plot shows a time window 7–8 days after virus injection. Mean ± s.e.m for n = 20 control mice during the same time window is shown for comparison (black).

Acute inhibition of GABAergic and serotonergic cells decreased sleep for a period lasting around 3 h (Fig. 4j). To test whether chronic inhibition leads to a long-term decrease in sleep propensity, we virally expressed Kir2.1 in both cell types. Strikingly, chronic co-inhibition reduced NREM sleep by nearly 70% relative to controls, and increased the mean length of wake bouts by more than twofold (Fig. 5a–f and Extended Data Fig. 11a). Chronic co-inhibition was lethal in around 17% of cases, probably owing to even greater reductions in sleep (Extended Data Fig. 11e,f). In surviving Kir2.1 mice, a marked reduction in sleep persisted during multiple weeks of observation (Fig. 5b and Extended Data Fig. 11b). Despite being awake for over 6.5 h more per day than control mice, surviving Kir2.1 mice exhibited lower delta power during unperturbed conditions. Whereas control mice reached peak delta power values around 150% over baseline, Kir2.1 mice exhibited peak values of only around 115% (Fig. 5g). These results indicate that long-term inhibition of GABAergic and serotonergic cells considerably alters sleep pressure accumulation kinetics. Indeed, expressing Kir2.1 in both cell types substantially reduced delta power accumulation during wake bouts (Fig. 5h). Slower delta accumulation in these mice was not caused by an increased proportion of quiet wakefulness. On the contrary, the robust decrease in delta accumulation was particularly striking given that Kir2.1 mice spent more time in high-theta wakefulness (Extended Data Fig. 11c,d)—a wake substate that correlates with greater behavioural arousal and is thought to play a privileged part in sleep pressure accumulation^49,50^. Consistent with these results, Kir2.1 mice accumulated less delta power during sleep deprivation, and did not exhibit either sleep attempts during deprivation or significant rebound sleep during recovery, despite their chronically reduced sleep (Fig. 5i–m). Thus, inhibition of GABAergic or serotonergic cells profoundly and persistently decreases behavioural and physiological indicators of sleep drive.

## Co-inhibition allows high-arousal wake maintenance

**Extended Data Fig. 12:**
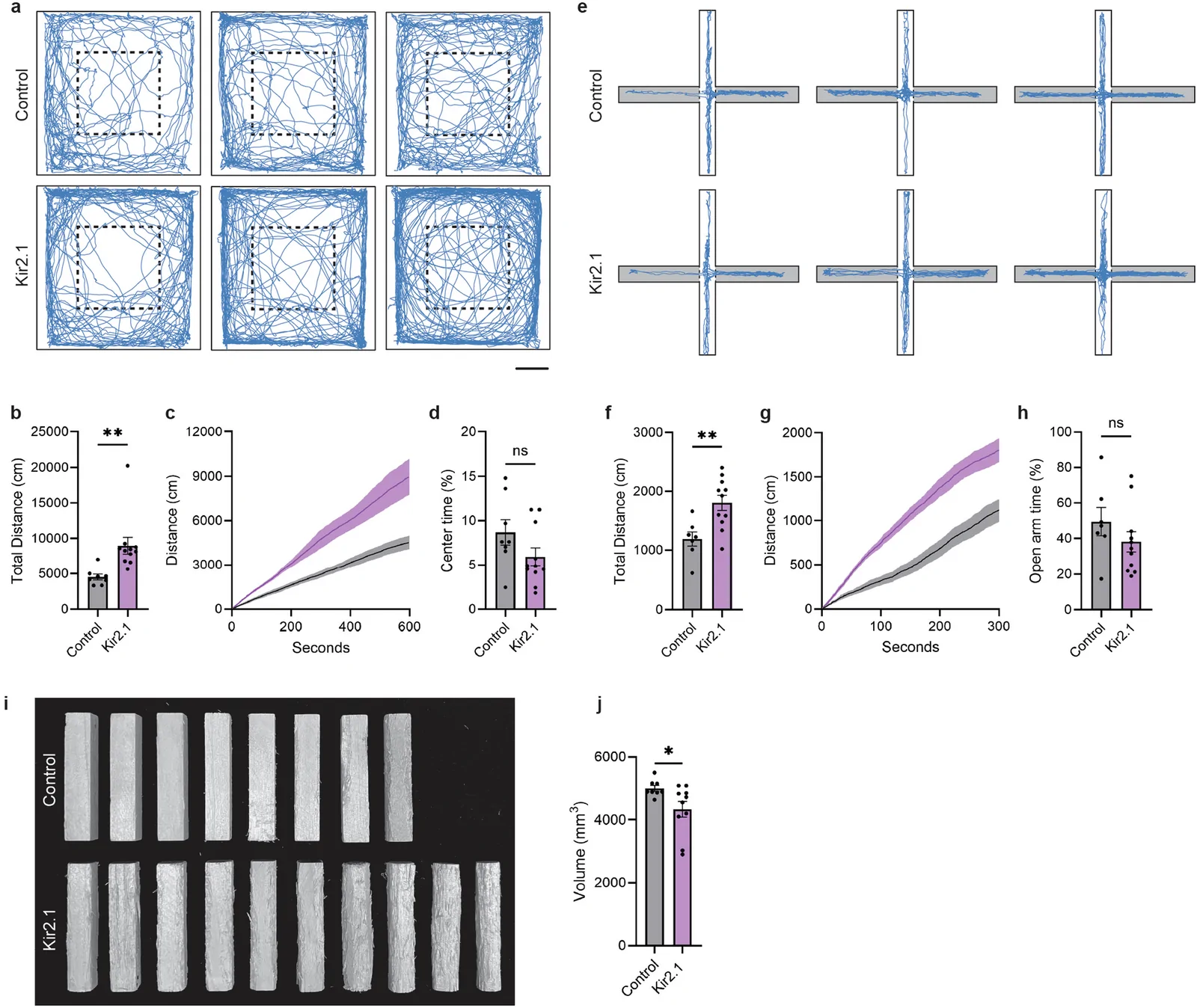
Behavioural analyses for MR chronic inhibition. Cre-dependent Control EGFP or Kir2.1 virus was injected into the MR in *Vgat-Cre*; *Sert-Cre* mice. **a**, Locomotor traces for three representative control (top) or Kir2.1 (bottom) mice in the open field test. Dotted line indicates centre of the arena. Scale bar, 10 cm. **b**, Total distance travelled during the 10 min of the assay. Grey, control, n = 8; magenta, Kir2.1, n = 11. Each data point represents one mouse. **p = 0.0064, unpaired two-tailed *t*-test. **c**, Cumulative distance travelled. **d**, Per cent time spent in the centre zone. ns, not significant, unpaired two-tailed *t*-test. **e**, Locomotor traces for three representative individual control (top) or Kir2.1 (bottom) mice in the elevated plus maze assay. Shaded areas indicate enclosed arms. **f**, Total distance travelled during the five minutes of the assay. **p = 0.0052, unpaired two-tailed *t*-test. **g**, Cumulative distance travelled. **h**, Per cent time spent on the open arms. ns, not significant, Unpaired two-tailed *t*-test. All data presented as mean ± s.e.m. **i**, Greyscale image of wood blocks exposed to individual control or Kir2.1 mice, for a period of 18 days in the home cage, arranged in order of surface abrasion. Wood block could not be recovered from one Kir2.1 mouse, and was presumed to have completely disintegrated. **j**, Wood blocks from each mouse were 3D scanned using a tripod-mounted Shining Einscan Pro HD and turntable, and reconstructions were quantified for total volume. Control n = 8 blocks, Kir2.1 n = 10 blocks. *p = 0.0386, unpaired two-tailed *t*-test.

Although chronic co-inhibition was lethal in around 17% of cases, most mice survived. We thus combined multiple behavioural analyses to examine how the chronic sleep loss of surviving Kir2.1 mice affects behaviour and cognition. Kir2.1 mice showed higher levels of locomotion, but did not exhibit significantly increased anxiety-like behaviours in the open field or elevated plus maze assays (Extended Data Fig. 12a–h). Kir2.1 mice also spent more time interacting with wood blocks, as evidenced by the increased surface abrasion and reduced volume of blocks from Kir2.1 mice relative to controls (Extended Data Fig. 12i,j). This suggests that Kir2.1 mice can maintain high behavioural engagement despite their chronic sleep loss, a hypothesis that is also supported by their sustained interaction with novel objects during sleep deprivation (Fig. 5l).

**Extended Data Fig. 13:**
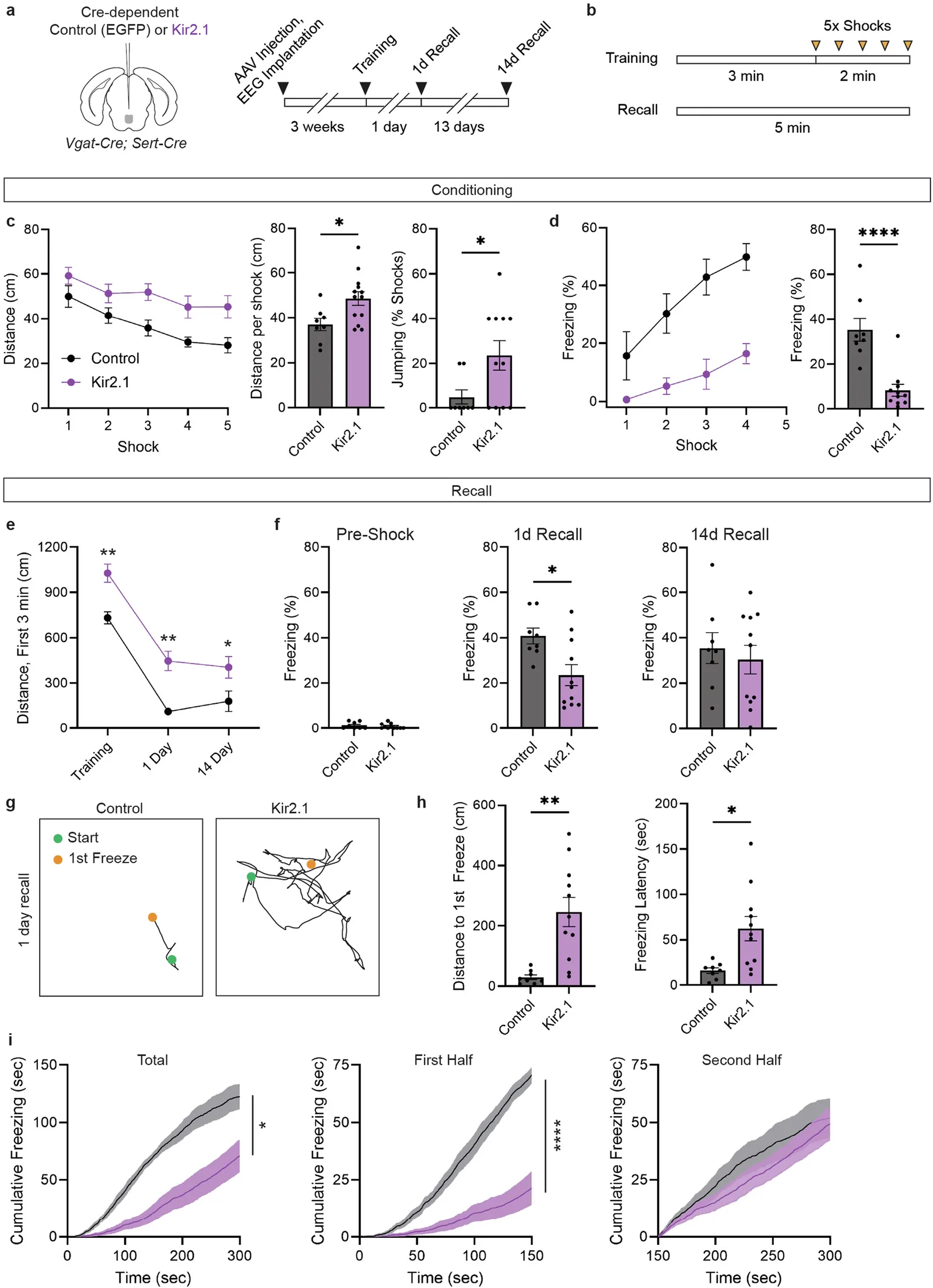
MR Kir2.1 mice form contextual fear memories. **a**, Strategy to test contextual fear memory. Kir2.1 or EGFP was virally expressed in MR GABAergic and serotonergic cells. Three weeks after virus injection, mice were trained in a contextual fear conditioning task, in which they learn to associate a specific environment (the conditioned stimulus, CS) with an aversive experience, a series of 5 foot shocks (unconditioned stimulus, US). The encoded memory was tested 1 and 14 days after training, by re-exposing mice to the conditioned context in the absence of shocks (CS without US). Freezing behaviour was used a readout for memory strength and persistence. **b**, Schematic of the contextual fear conditioning task. During training, mice were allowed to explore a specific environment for 3 min, before 5 electric shocks were delivered at 30 s intervals. During recall, mice were re-introduced to the same environment for 5 min without any shocks. **c**, Left, horizontal distance travelled in response to each of the five training shocks, measured in the 2 s following each shock. Middle and right, Kir2.1 mice travel greater horizontal distances and exhibit more frequent jumping behaviour in response to shocks. Grey, control; magenta, Kir2.1 throughout figure. *p = 0.0165 (distance per shock), *p = 0.0357 (jumping), Unpaired two-tailed *t*-test. **d**, Kir2.1 mice freeze less than controls in between shocks. ****p < 0.0001, unpaired two-tailed *t*-test. **e**, Distance travelled during the first three minutes of the training or recall sessions. **p = 0.0041 (training), **p = 0.0010 (1 day recall), *p = 0.0397 (14 day recall), two-way repeated-measures ANOVA. **f**, Per cent time spent freezing during the 3-minute pre-shock period during training (left), or during the 5-minute recall sessions 1 day or 14 days after training (middle and right panels). *p = 0.0123, unpaired two-tailed *t*-test. **g**, Locomotion trace between start point and first freeze bout during 1 day recall. **h**, Kir2.1 mice travel greater distances and exhibit longer latencies before the first freeze bout. **p = 0.0015 (distance to 1st freeze), *p = 0.0103 (latency), unpaired two-tailed *t*-test. **i**, Cumulative freezing time during the 5-minute recall period. Kir2.1 mice freeze less during the first half of the recall session, but are indistinguishable from controls during the second half. *p = 0.0123, ****p < 0.0001, unpaired two-tailed *t*-test. **c**–**f**,**h**,**i**, All data are mean ± s.e.m. The brain schematic in **a** was adapted from ref. ^76^, Springer Nature Limited, under a Creative Commons licence CC BY 4.0.

Because sleep deprivation can disrupt learning and memory processes^51–54^, we also trained Kir2.1 mice in a contextual fear conditioning task, in which mice learn to associate a specific environment with an aversive experience (foot shocks; Extended Data Fig. 13a,b). Memory strength and long-term persistence were assessed by reintroducing mice to the same conditioning environment at recent (1 day) or remote (14 days) time points, and measuring freezing behaviour and locomotion in the absence of shocks. As in the open field and elevated plus maze assays, Kir2.1 mice exhibited increased locomotion during conditioning and recall (Extended Data Fig. 13c–e). Notably, all Kir2.1 mice were able to encode long-lasting memories, and froze significantly more than at naive (pre-shock) baseline levels up to 14 days after conditioning (Extended Data Fig. 13f). Memory strength was modestly—albeit significantly—weaker in Kir2.1 mice than in controls for recent recall (1 day), probably due to increased mobility when Kir2.1 mice were first re-introduced into the training context (Extended Data Fig. 13g–i). Despite this initial decrease, memory strength reached levels comparable with those of control mice by remote recall (14 days). Thus, chronic sleep loss of nearly 70% does not markedly compromise the encoding or long-term storage of an associative memory in these mice. Cumulatively, our analyses suggest that MR inhibition allows surviving mice to maintain high-arousal wakefulness for extended time periods without corresponding increases in sleep drive, and without the strong memory deficits that can follow severe sleep-deprivation protocols^51–54^.

## Discussion

Our study presents three main advances. First, we map brain-wide activity signatures that reflect both spontaneous and experimentally induced changes in sleep drive. Second, we identify neuronal populations in the MR and aMPO that are necessary and sufficient to generate increased sleep drive. Third, we persistently and drastically reduce sleep drive by inhibiting MR GABAergic and serotonergic neurons—a manipulation that produces lethality in a fraction of mice, but in most cases allows lasting maintenance of wakefulness without the behavioural deficits of severe sleep deprivation.

**Extended Data Fig. 14:**
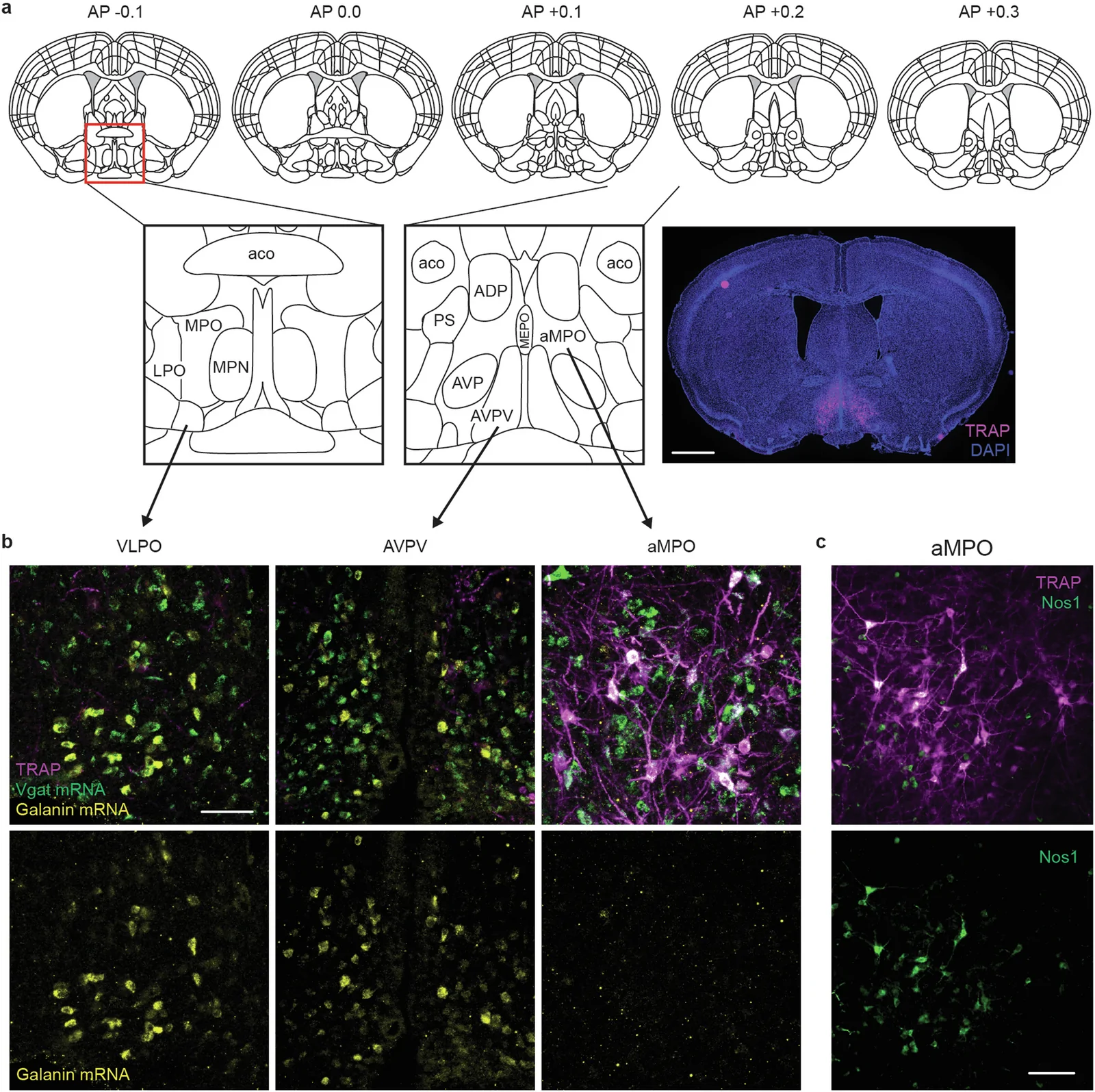
aMPO deprivation-TRAP cell identity. **a**, Schematic of the anterior medial preoptic area (aMPO) in anatomical context. Bottom right, representative coronal section with aMPO deprivation-TRAP cells (magenta) and DAPI counterstain (blue). Scale bar, 1 mm. aco, anterior commissure; MPO, medial preoptic area; LPO, lateral preoptic area; VLPO, ventrolateral preoptic area; ADP, anterodorsal preoptic nucleus; PS, parastrial nucleus; MEPO, median preoptic nucleus; aMPO, anterior medial preoptic area; AVP, anteroventral preoptic nucleus; AVPV, anteroventra paraventricular nucleus. **b**, *Vgat* and *Gal* expression in the ventral lateral preoptic area (VLPO), anteroventral paraventricular nucleus (AVPV), and aMPO. aMPO deprivation-TRAP cells (Magenta) express *Vgat* mRNA (Green) but not *Gal* mRNA (Yellow). Scale bar, 50 µm. **c**, aMPO deprivation-TRAP cells express NOS1 protein (green). Scale bar, 100 µm. **b**,**c**, Representative images selected from *n* = 4 replicates. The brain schematics in **a** were adapted from ref. ^76^, Springer Nature Limited, under a Creative Commons licence CC BY 4.0.

Although they are likely to act as part of a complex system that regulates sleep drive, several properties of the MR and aMPO suggest that they are key regulators. First, activation of cells in these regions increases during wakefulness and declines during periods of sleep. This pattern conceptually resembles the accumulation and dissipation of sleep pressure, and suggests that these cells have functions distinct from those of previously characterized sleep-active populations, such as LPO galanin^+^ cells^27^ or cortical nNOS^+^ cells^55,56^. Indeed, aMPO deprivation-TRAP cells expressed *Vgat* but not *Gal* (Extended Data Fig. 14), further distinguishing them from known sleep-promoting neurons in the preoptic area. Second, MR and aMPO manipulations can control sleep drive bidirectionally. Activating deprivation-responsive cells mimics high sleep pressure by increasing sleep duration and intensity, whereas inhibiting them attenuates the accumulation of sleep pressure and allows mice to maintain longer wake bouts before succumbing to sleep.

Our results diverge from those of other manipulations that decrease sleep by targeting either sleep-promoting or arousal-promoting systems. On the one hand, perturbations that decrease sleep amount do not necessarily reduce sleep drive. For example, rats with LPO lesions sleep less but initiate sleep more frequently, suggesting intact sleep propensity^28^. Similarly, mice that lack nNOS sleep less but are excessively sleepy, particularly during sleep deprivation^56^. These perturbations thus compromise normal sleep and the dissipation of sleep drive, whereas the MR and aMPO probably contribute to the build-up or detection of sleep drive. On the other hand, manipulations that increase arousal typically increase sleep drive as well. For example, activating specific cell types in the ventral tegmental area, lateral hypothalamus, preoptic area or basal forebrain strongly increases high-arousal wakefulness^50,57–59^, as does systemic administration of cocaine or methamphetamine^60,61^. All of these manipulations produce compensatory increases in NREM sleep duration and/or delta power. By contrast, mice with inhibited MR and aMPO cells exhibit fewer attempts to sleep, less rebound sleep and lower delta-power accumulation despite increased wake time (Extended Data Fig. 6 and Fig. 5). Much of this time is spent in high-arousal wakefulness, but with lower-than-expected behavioural or EEG markers of sleep debt. Chronic MR inhibition was lethal in around 17% of cases, perhaps owing to the physiological costs of sustained sleep loss. Notably, most mice with chronic MR inhibition survived and were able to maintain highly engaged wakefulness. They also successfully encoded persistent memories despite severe and long-term sleep reductions (Extended Data Figs. 11–13). MR manipulations could thus provide a new context in which to study the long-term effects of sleep loss, as well as the mechanisms that confer resilience to sleep deprivation.

The effects of MR and aMPO inhibition also differ from natural short-sleeper phenotypes in mice and humans. Mice with specific mutations from short-sleeper families and human habitual short sleepers both exhibit increased wakefulness and delta power, suggesting that they can tolerate higher sleep pressure^8,62^. MR and aMPO inhibition again contrasts with these cases, with mice exhibiting lower delta power despite extended wakefulness. Altogether, our observations suggest the intriguing possibility that these mice are not chronically sleep deprived, but rather, that they accumulate less sleep pressure.

Whereas investigations into raphe sleep functions have focused mainly on serotonergic neurons in the dorsal raphe^38,39,63,64^, our cell-type-specific manipulations reveal crucial roles for non-serotonergic populations in the MR. In particular, MR GABAergic cells are required for normal sleep, and their activation can increase sleep duration and intensity. However, both GABAergic and serotonergic populations become activated during sleep deprivation, which suggests that they cooperate to promote sleep during extended wakefulness. Co-activation of both cell types promoted sleep for longer than activation of either population alone (Fig. 4), whereas co-inhibition reduced baseline sleep profoundly and abolished post-deprivation rebound sleep (Figs. 4 and 5). These results are consistent with synergistic interactions, perhaps mediated by local signals in the MR or by the many overlapping targets of GABAergic and serotonergic projections^43,44^. Meanwhile, MR *Vglut2*^+^ cells were necessary and sufficient to promote wakefulness (Fig. 4), complementing previous experiments in which *Vglut2*^+^ cell activation promoted exploratory behaviour on shorter, minutes-long timescales^65^. The MR can thus modulate behavioural state powerfully and in a cell-type-specific manner. Future experiments will be able to extend these observations by further characterizing the functions of MR neuron molecular subtypes, as well as their neuronal activity dynamics during sleep-drive accumulation and dissipation.

According to the ‘flip-flop switch’ model, reciprocal inhibition between sleep- and wake-active neuronal populations stabilizes behavioural states and allows rapid state switching while minimizing hybrid states^66^. It remains unclear how sleep drive regulates the switch. In this framework, MR deprivation-responsive neurons are well positioned to bias the switch towards sleep. Their broad projections include key switch components; for example, suppressing the wake-associated LHA and promoting sleep through the LPO (Fig. 3). However, because MR deprivation-responsive cells also increase sleep intensity, their functions extend beyond simply initiating sleep transitions. MR wake-associated activity might induce lasting changes in downstream targets to modulate both the amount and the quality of ensuing sleep. Because deprivation-responsive cells do not project directly to cortical areas (Fig. 3), they might regulate NREM oscillations through their thalamic projections or via other indirect pathways. The relative contributions of these targets require further clarification, as do the functions of the broad input sources to the MR^44,67^.

The mechanisms that increase MR activation during sleep deprivation remain unclear, as does their relationship to electrical activity in specific MR cell types. Sleep deprivation increases the excitability of MR GABAergic cells by depolarizing membrane potential and lowering firing threshold. GABAergic cells are thus more likely to spontaneously fire action potentials, and exhibit higher firing rates in sleep-deprived mice (Fig. 5). This increased excitability and firing could reflect both cell-autonomous changes and increased presynaptic inputs, or other types of synaptic or structural plasticity during prolonged wakefulness^68,69^. Conceptually related mechanisms regulate sleep in *Drosophila*^9,70^ as well as motivated behaviours such as feeding, mating and aggression in several species^13,71–75^. According to these scenarios, physiological changes in specific deprivation-responsive neurons might determine whether an animal maintains wakefulness or engages in sleep-preparatory behaviours and transitions to sleep. By identifying neuronal populations that control sleep drive, our results provide entry points to further dissect the circuit and molecular mechanisms that ultimately make sleep inescapable.

## Methods

### Mice

Mice were maintained on a 12 h:12 h light:dark cycle with ad libitum access to food and water, at 22 ± 2 °C and 55 ± 10% humidity. All experiments were performed in accordance with the Swiss National Institutional Guidelines on Animal Experimentation and were approved by the cantonal Veterinary Office Committees for Animal Experimentation. Male C57BL6/J mice were used for whole-brain FOS mapping during circadian and sleep-deprivation time points, and both males and females were used for all other experiments. Age-matched mice (2–6 months) of the appropriate genotype were randomly distributed into experimental groups. Knock-in and transgenic lines were obtained from the Jackson Laboratory, including the TRAP2 knock-in line (*Fos-2A-iCreERT2*, strain 030323)^40^, *Ai14* Cre reporter (*RCL-tdT*, strain 007914), *Sert-Cre* (*Slc6a4*^*tm1(cre)Xz*^, strain 014554)^77^, *Vgat-IRES-Cre* (*Slc32a1*^*tm2(cre)Lowl*^, strain 028862)^78^ and *Vglut2*-*IRES-Cre* (*Slc17a6*^*tm2(cre)Lowl*^, strain 016963)78.

### Sleep deprivation and sleep attempts

Nesting material was removed from the cage at the beginning of sleep deprivation, and replaced at the end of the deprivation period. Mice were housed individually for all deprivation experiments. For grooming-based sleep deprivation, mice were finely misted with Milli-Q water to elicit grooming behaviour every 15 min for up to 6 h. Sleep attempts were interrupted by opening the cage door or applying an additional water mist. For novel-object-based sleep deprivation, a new object was placed in the cage every 15 min to maintain behavioural engagement for up to 6 h. Sleep attempts were interrupted by opening the cage door and displacing the object. Sleep attempts were defined as the adoption of a sleep-like posture and the concurrent emergence of NREM-like slow-wave activity in live EEG recordings, which reverted to wake-like EEG after cage-door opening, water misting or novel object displacement, and could be detected post hoc as sleep bouts by automated state classification (see Extended Data Fig. 7 and Methods sections below).

### Whole-brain immunostaining and imaging

For all mapping experiments, mice were housed individually and allowed to habituate to the home cage for several days. For circadian mapping, whole brains were collected every 3 h during the unperturbed light–dark cycle. For deprivation mapping, whole brains were collected 1.5 h, 3 h, 5 h and 6 h into sleep deprivation, as well as 1.5 h and 3 h into the recovery period after 6 h deprivation. Six to seven brains per time point were collected for the circadian experiment and light-phase sleep deprivation, and four brains per time point were collected for dark-phase sleep deprivation. All samples were immunostained and cleared according to the iDisco^79^ workflow (https://idisco.info/) and imaged in horizontal orientation on a MesoSPIM system (Center for Microscopy and Image Analysis, University of Zurich) with corresponding control software (https://github.com/mesoSPIM/mesoSPIM-control) and a Hamamatsu Orca camera, using the following settings: 1.25× zoom, z_stepsize 5.0 μm, pixel size 5.26 μm, 2,048 × 2,048 resolution. Immunostaining was performed using a polyclonal rabbit anti-FOS antibody (Synaptic Systems, 226 003) at 1:2,000 and donkey anti-rabbit Alexa Fluor 647 (Invitrogen, A31573) at 1:800. Autofluorescence from the 561-nm channel was collected for overall brain morphology.

### Whole-brain FOS mapping

Whole-brain light-sheet data were registered to an average mouse-brain template using the SHIELD-based workflow^24^. For each brain sample, striping artefacts were removed from the raw autofluorescence channel before resizing to the dimensions of a 461 × 471 × 323-pixel light-sheet-optimized average brain template^25^ (https://github.com/Gubra-ApS/LSFM-mouse-brain-atlas) and performing automated non-rigid alignment with sitk-align. Alignment results were inspected and manually corrected by reiteratively adjusting data:atlas correspondences and re-warping images in nuggt (Neuroglancer ground truth; https://github.com/chunglabmit/nuggt, master) using the nuggt-align function^24^. In parallel, FOS^+^ nuclei were automatically detected in three-dimensional (3D) stacks using a Laplacian of Gaussian filter (skimage.feature.blob_log, scikit-image v.0.19.3, https://scikit-image.org/). Identical alignment parameters were then applied to FOS^+^ nuclei from the same sample. To create heat maps of FOS^+^ cell density for each brain sample, Gaussian blur (sigma = 2.25) was applied to aligned FOS nuclei and the signal was averaged between the left and right brain hemispheres, before performing batch correction based on the ComBat approach^80^. To evaluate distinct patterns of brain activation across experimental conditions, averages for mice within the same experimental time point were normalized across time points within each experiment by dividing by the total activation per voxel. We then segregated each experimental dataset by peak activation time point, resulting in four categories per experimental condition: late wake (zeitgeber time (ZT)15–ZT21 circadian, sleep deprivation (SD) time points SD5h and SD6h for deprivation), sleep (ZT03–ZT09 circadian, recovery (R) time points R1.5h–R3h for deprivation), early wake (ZT12 circadian, SD1.5h–SD3h deprivation) and transition (ZT00 circadian, ZT12 dark deprivation). Finally, hierarchical clustering (clustering depth of 20) was performed for each category, and each cluster was manually categorized as type 1–type 3 on the basis of its mean response pattern during sleep deprivation and recovery. Whole-brain datasets can be browsed at: https://sleep-wake-atlas.scicore.unibas.ch/, and code for all analyses is available at https://gitlab.com/ceda-unibas/sleep-brain-atlas. For Pearson correlation analyses using unperturbed circadian data (Extended Data Fig. 3), voxel intensity correlations with wake duration were calculated for a 2-h time window preceding each experimental time point.

### EEG analyses

For EEG implantation, electrodes were stereotactically placed over the right cerebral hemisphere at anteroposterior (AP) −2.25; lateral (L) +1.7 and AP +1.5, L +1.2 relative to bregma. Custom EEG implants were fixed to the skull using superglue and orthodontic resin (Paladur, Kulzer). All mice were allowed to recover from surgery for at least one week in their home cages. Mice were then connected to a flexible recording cable with a commutator and allowed to habituate to custom-made behaviour cages for at least 3 days, before EEG and video tracking were collected continuously for the duration of each experiment. Data were acquired using a 16-channel AC amplifier (A-M Systems, model 3500), filtered (0.3–300 Hz) with a gain of 500, digitized at 200 Hz and recorded with Spike2 (v.9.09a). The EEG signal was downsampled to 100 Hz and partitioned into 2-s epochs. Behavioural states were classified as high-theta wake, low-theta wake, NREM sleep and REM sleep using custom software (offline electroencephalography state space analysis (OESSA); https://github.com/VBits/oessa). EEG recording was used to quantify sleep behaviour in all cases. For the sleep-deprivation FOS-mapping experiments in Fig. 1, only a subset of representative mice was recorded given continuous wake maintenance by manual sleep deprivation. EEG power spectra in the 0–50 Hz range were calculated using a fast Fourier transform at 0.25-Hz resolution. To account for inter-individual variability in EEG signal, spectral data for each mouse were normalized to the total power for each vigilance state and shown as a percentage. Delta power (0.25–4 Hz) time-course data were normalized to mean baseline values from the last four hours of the light phase, when delta power is lowest^2,81^. To evaluate the relationship between wake duration and NREM delta power, a three-term filter for wake-enriched episodes^42^ was applied to EEG data from a 5-day undisturbed baseline period: NREM ≥ 10 min preceding wake ≥ 15 min, immediately followed by NREM ≥ 15 min. NREM segments allowed brief wake or REM intrusions of 1 min or less, and wake-enriched segments allowed brief NREM intrusions of 3 min or less.

### TRAP induction

4-Hydroxytamoxifen (4-OHT; Sigma H6278) was dissolved in ethanol at 20 mg ml^−1^ and combined with two volumes of a 4:1 blend of sunflower seed oil:castor oil (Sigma, S5007 and 259853). After mixing at room temperature for 2–3 h, ethanol was evaporated by vacuum centrifugation and the final 10 mg ml^−1^ solution was injected intraperitoneally (i.p.) at a dose of 50 mg kg^−1^. Mice were habituated to handling and i.p. injection for 4–5 days before TRAP induction. For deprivation-TRAP experiments, 4-OHT was administered 5 h into a 6-h sleep-deprivation period starting at the beginning of the light phase. For recovery-TRAP, 4-OHT was administered 3 h after the end of the 6-h sleep-deprivation period. For rested control-TRAP, 4-OHT was administered to undisturbed mice 5 h into the light phase. Deprivation-TRAP cells in the MR were labelled during induced grooming-based sleep deprivation, which yielded similar results to novel-object-based deprivation-TRAP (see Extended Data Fig. 5). aMPO deprivation-TRAP cells were labelled using novel-object-based sleep deprivation.

### Chemogenetics

Under surgical anaesthesia, AAV5-hSyn-DIO-hM3Dq-mCherry (Addgene 50474), AAV5-hSyn-DIO-hM4Di-mCherry (Addgene 50474), AAV9-hSyn-DIO-hM4Di-mCherry or AAV5-Syn-FLEX-PSAM4-GlyR-IRES-EGFP (Addgene 119741) were stereotactically injected into the MR, aMPO, LPO or LHA with a Nanoject III (Drummond Scientific). Coordinates relative to bregma and injection volumes were as follows: MR: AP −2.28; dorsoventral (DV) −4.90, angled 22° anteriorly relative to the horizontal plane, 100 nl; aMPO: AP +0.80; L ±0.28; DV −5.10, 45 nl bilateral; LPO: AP 0.00; L ±0.70; DV −5.20, 100 nl bilateral; LHA: AP −1.60; L ±1.03; DV −4.95, 100 nl bilateral. For all sleep classification experiments, custom EEG implants were attached immediately following virus injection. For TRAP experiments, 4-OHT was administered 2 weeks after virus infusion, and chemogenetics experiments started 3–4 weeks later. All other chemogenetics experiments commenced 3–4 weeks after virus injection. Mice were habituated to handling and i.p. injection for 3 days before each experiment. The same mice were then injected on consecutive days with either control phosphate-buffered saline (PBS) or 1 mg kg^−1^ CNO (Sigma, SML2304), a dosage chosen on the basis of the absence of CNO-induced phenotypes in control mice. For experiments mapping MR activation, AAV5-hSyn-DIO-hM3Dq was injected into the MR in TRAP2 mice. Three weeks after deprivation-TRAP induction, CNO was administered at the beginning of the dark phase and mice were euthanized 1.5 h later for iDisco-based FOS analyses. For MR-projection-based manipulations, AAVretro-EF1a-FlpO (Addgene 55637) was injected into the LPO or dorsomedial hypothalamus. After 1–2 weeks, AAV8-EF1a-Con/Fon-hM3Dq-mCherry (Gene Vector and Virus Core, Stanford University) was injected into the MR, and PBS or CNO was administered 3–4 weeks later. Viral targeting was confirmed post-mortem, and mice with off-target expression were excluded from the experiment.

### Anterograde tracing

AAV9-hSyn-FLEX-mGFP-2A-Syp-mRuby (Viral Core Facility, Charité–Universitätsmedezin Berlin) was injected into the MR in TRAP2 mice 2 weeks before deprivation-TRAP induction. Brains were collected for sectioning and immunostaining 3–4 weeks after TRAP induction. Synaptophysin-mRuby puncta were registered to Allen Mouse Brain CCFv3 using the QUINT workflow^82^.

### Chronic manipulations and cell ablation

For chronic inhibition of deprivation-TRAP cells, AAVdj-CMV-DIO-Kir2.1-2A-ZsGreen or AAVdj-CMV-DIO-EGFP (Gene Vector and Virus Core, Stanford University) was injected into the MR 1–2 weeks before TRAP induction as described above. Sleep behaviour was analysed 3–4 weeks after TRAP induction. For TRAP cell ablation, a mix of AAV9-EF1a-FLEX-DTA (University of Zurich Viral Vector Facility) and AAV5-EF1a-DIO-YFP (Addgene 27056) was injected into the MR 1–2 weeks before TRAP induction. In cell-type-specific ablation experiments, a mix of AAV5-FLEX-taCasp3-TEVp (Addgene 45580) and AAV5-EF1a-DIO-YFP was injected into the MR of *Sert-Cre*, *Vgat-IRES-Cre* and *Vglut2-IRES-Cre* mice. For all ablation experiments, control mice were injected with AAV5-EF1a-DIO-YFP only. Sleep behaviour was analysed 3–4 weeks after TRAP induction or virus infusion, and cell ablation was confirmed post-mortem.

### Immunohistochemistry

Mice were perfused with chilled PBS followed by chilled 4% paraformaldehyde (PFA). Brains were post-fixed overnight at 4 °C before cryoprotection in 30% sucrose. Cryosections (40 μm) were washed in PBS, incubated in blocking solution (10% normal donkey serum, 0.2% Triton X-100 in PBS) for 30–60 min at room temperature and incubated for 2–3 days in primary antibody. Sections were then washed at room temperature with 0.2% Triton X-100 in PBS and incubated for 1–3 days in secondary antibody. After washing in 0.2% Triton X-100 in PBS, sections were stained with DAPI and mounted on slides with Fluoromount-G (Invitrogen). Images were acquired with a Zeiss AxioScan.Z1 or a Zeiss LSM800 confocal microscope, using respective AxioScan and ZEN Blue software. Primary antibodies: rabbit anti-FOS (polyclonal, Synaptic Systems 226 003, 1:1,000-1:4,000), rabbit anti-FOS (monoclonal, Synaptic Systems 226 008, 1:2,000), rat anti-FOS (Synaptic Systems 226 017, 1:2,000), chicken anti-GFP (Invitrogen A10262, 1:1,000), rabbit anti-ZsGreen (Takara 632474, 1:1,000), rat anti-RFP (Chromotek 5f8-150, 1:1,000), goat anti-tdTomato (Origene AB8181, 1:1,000), goat anti-5HT (Immunostar 20079, 1:2,000), guinea pig anti-orexin A (Synaptic Systems 389 004, 1:500), rabbit anti-MCH (Phoenix, H-070-47, 1:500) and mouse anti-nNOS (Sigma N2280, 1:500). Primary antibodies were matched with appropriate donkey or goat secondary antibodies conjugated to Alexa 488, Alexa 546, Alexa 555, Cy3, Alexa 647 or Cy5, and used at 1:750-1:1,000 (Thermo Fisher Scientific and Jackson ImmunoResearch).

### In situ hybridization

Multiplex HCR (hybridization chain reaction) probe sets for *Slc32a1* (*Vgat*), *Slc7a6* (*Sert*) and *Gal* and corresponding amplifiers were used according to the manufacturer’s instructions (Molecular Instruments) with modifications. In brief, 40-μm cryosections were collected as described above, incubated in 70% ethanol for 2 h at room temperature and rinsed in 5× SSCT before the pre-hybridization step. After overnight probe hybridization, sections were washed in a 100%/75%/50%/25%/0% probe wash buffer/5×SSCT series at 37 °C, before proceeding to amplification, washes and mounting. For HCR in situ and immunohistochemistry in the same sections, HCR was completed first and sections were fixed in 4% PFA for 10 min at room temperature before proceeding to immunohistochemistry.

### Electrophysiology

All recordings and analyses were performed blinded to experimental groups. To label GABAergic or serotonergic cells, AAV9-CAG-DIO-tdTomato or AAV5-hSyn-DIO-tdTomato was injected into the MR in 2–4-month old *Vgat-Cre* or *Sert-Cre* mice, respectively. Approximately 2 weeks later, mice were euthanized 6 hours after the beginning of the light phase, immediately after a 6-h sleep deprivation or after being left undisturbed. Coronal slices (200 μm) containing the MR were cut in carbogenated (95% O_2_/5% CO_2_) ice-cold cutting solution (sucrose 205 mM, glucose 10 mM, NaHCO_3_ 25 mM, KCl 2.5 mM, NaH_2_PO_4_ 1.25 mM, MgCl_2_ 7.5 mM and CaCl_2_ 0.5 mM) with a Leica VT1200S vibratome (horizontal oscillation amplitude 1.80 mm). Slices were kept at 33.0 ± 1 °C in carbogenated artificial cerebrospinal fluid (aCSF: NaCl 125 mM, KCl 2.5 mM, NaH_2_PO_4_ 1.2 mM, NaHCO_3_ 24 mM, sodium ascorbate 5 mM, glucose 12.5 mM, MgCl_2_ 1 mM and CaCl_2_ 2 mM, pH 7.4) for 30 min and then kept at room temperature for around 1 h before starting recordings.

During recording sessions, slices were held at 33.0 ± 1 °C in a custom chamber with carbogenated aCSF circulation. Whole-cell patch clamp recordings of tdTomato^+^ MR GABA or serotonin cells were performed in current clamp mode using pClamp11 software with a Multiclamp 700B amplifier (Molecular Devices). Cells were visualized using an upright microscope equipped with gradient contrast infrared visualization (Luigs and Neumann) and a 60× objective. For all experiments, data were digitized by a Digidata 1440a (Molecular Devices) at 10 kHz and filtered at 1 kHz. Patch pipettes (4–8 MΩ) were pulled with a Sutter Instruments P-1000 micropipette puller and filled with the following intracellular solution: 142 mM potassium gluconate, 10 mM HEPES, 1 mM EGTA, 2.5 mM MgCl_2_, 4 mM Mg-ATP, 0.3 mM Na-GTP and 10 mM Na-phosphocreatine, with 0.2% biocytin. Passive membrane properties and action potentials were quantified using Clampfit 10 (Molecular Devices).

Sections were fixed immediately after recordings, and recorded cell identity was confirmed post hoc using streptavidin–Alexa488 or streptavidin–Alexa647 (Thermo Fisher Scientific), together with tdTomato immunostaining as described above.

### Open field test, elevated plus maze and wood block engagement

For all behavioural assays, AAVdj-CMV-DIO-Kir2.1-2A-ZsGreen or AAVdj-CMV-DIO-EGFP (control) was injected into the MR in *Vgat-Cre*;*Sert-Cre* double transgenic mice 3 weeks before experiments. EEG recordings were analysed to confirm sleep loss phenotypes before testing. Both open field and plus maze tests were done at the beginning of the dark phase, when mice naturally spend more time awake. For the open field test, mice were placed in the centre of a 50 × 50-cm square box with 30-cm walls, and video was recorded from above for 10 min at 25 fps. For the plus maze assay, mice were placed at the centre of a maze with 35 cm × 6-cm arms, 74 cm above the ground, and video was recorded for 5 min. Locomotion, time in the open field centre and time in the closed arms were quantified using open source OptiMouse software (https://github.com/yorambenshaul/optimouse)^83^.

To assess wood block engagement, 1 × 1 × 5-cm wood blocks (Labodia 213-1011) were placed in the home cage for 18 days, and then 3D scanned using a tripod-mounted Shining Einscan Pro HD and turntable. Total volume was quantified from 3D reconstructions.

### Contextual fear conditioning

Fear conditioning acquisition and recall sessions were performed at the beginning of the dark phase, when mice naturally spend more time awake. During acquisition sessions, mice were placed in the middle of a 25 × 25-cm square box with a 2% acetic acid odour source and an electrifiable grid floor (Fear Conditioning System, Ugo Basile, with EthoVision XT software v.14-17), and allowed to explore freely. After 3 min, five foot shocks (0.8 mA, 1 s) were delivered at 30-s intervals. Recall was assessed at recent (1 day) and remote (14 days) time points after the acquisition session. For recall sessions, mice were placed in the same training context, and allowed to explore freely for 5 min without any foot shocks. During all sessions, mouse behaviour was recorded with an overhead infrared camera (Basler acA1300-60gm, Basler GenICam). Memory recall was assessed by quantifying the fraction of time spent freezing in the shocked context. Freezing behaviour was automatically classified by the EthoVision XT software, and defined as bouts of complete immobility (apart from breathing) lasting at least 2 s (pixel change < 1–3% adjusted for each mouse), as previously published^84^.

### Statistics and reproducibility

Details regarding the number of mice and statistical tests for each experiment are provided in the figure legends. All statistical tests are two-sided. Sample sizes were chosen on the basis of previous experiments and published studies, and all experiments included at least three replicates. Statistical analyses were performed in Python or GraphPad Prism v.10.

### Reporting summary

Further information on research design is available in the Nature Portfolio Reporting Summary linked to this article.

## Online content

Any methods, additional references, Nature Portfolio reporting summaries, source data, extended data, supplementary information, acknowledgements, peer review information; details of author contributions and competing interests; and statements of data and code availability are available at https://doi.org/10.1038/s41586-026-10928-3.

## Supplementary information

Reporting Summary

Peer Review file

## Source data

Source Data Fig. 1

Source Data Fig. 2

Source Data Fig. 3

Source Data Fig. 4

Source Data Fig. 5

## Data Availability

All data will be made public or available upon request. Whole-brain atlas data are available at https://sleep-wake-atlas.scicore.unibas.ch/. Data from the Allen Brain Atlas CCFv3 and a previously published light sheet fluorescence microscopy (LSFM) optimized atlas^25^ are available at https://brain-map.org/atlases/anatomy and https://github.com/Gubra-ApS, respectively. Source data are provided with this paper.

## Code availability

Custom code will be made available upon request. EEG analysis code is available at https://github.com/VBits/oessa. Code for all whole-brain FOS analyses is available at https://gitlab.com/ceda-unibas/sleep-brain-atlas. Code to generate the corresponding web resource is available at https://gitlab.com/ceda-unibas/bav.
