# Supplementary material for: Wake-activated neuronal populations that regulate sleep drive
Source: Nature. 2026 Aug 19;658(8137):987–97. doi: 10.1038/s41586-026-10928-3 (PMC13686944; doi:10.1038/s41586-026-10928-3)
Supplement: Supplementary file 1 [file 41586_2026_10928_MOESM1_ESM.pdf]

nature portfolio

Corresponding author(s): Alexander F. Schier et al.

Last updated by author(s): May 12, 2026

Reporting Summary

Nature Portfolio wishes to improve the reproducibility of the work that we publish. This form provides structure for consistency and transparency in reporting. For further information on Nature Portfolio policies, see our Editorial Policies and the Editorial Policy Checklist.

Statistics

For all statistical analyses, confirm that the following items are present in the figure legend, table legend, main text, or Methods section.

n/a	Confirmed	
☐	☒	The exact sample size (*n*) for each experimental group/condition, given as a discrete number and unit of measurement
☐	☒	A statement on whether measurements were taken from distinct samples or whether the same sample was measured repeatedly
☐	☒	The statistical test(s) used AND whether they are one- or two-sided<br>*Only common tests should be described solely by name; describe more complex techniques in the Methods section.*
☐	☒	A description of all covariates tested
☐	☒	A description of any assumptions or corrections, such as tests of normality and adjustment for multiple comparisons
☐	☒	A full description of the statistical parameters including central tendency (e.g. means) or other basic estimates (e.g. regression coefficient) AND variation (e.g. standard deviation) or associated estimates of uncertainty (e.g. confidence intervals)
☐	☒	For null hypothesis testing, the test statistic (e.g. *F*, *t*, *r*) with confidence intervals, effect sizes, degrees of freedom and *P* value noted<br>*Give P values as exact values whenever suitable.*
☒	☐	For Bayesian analysis, information on the choice of priors and Markov chain Monte Carlo settings
☒	☐	For hierarchical and complex designs, identification of the appropriate level for tests and full reporting of outcomes
☒	☐	Estimates of effect sizes (e.g. Cohen's *d*, Pearson's *r*), indicating how they were calculated

Our web collection on statistics for biologists contains articles on many of the points above.

Software and code

Policy information about availability of computer code

Data collection	EEG data was collected using Spike2 v9.09a. Lightsheet imaging data was acquired using mesoSPIM control software (available at https://github.com/mesoSPIM/mesoSPIM-control). Confocal and brightfield imaging data was collected using ZEN Blue and AxioScan software from Zeiss.
Data analysis	EEG data was recorded using Spike2 (Version 9.09a), and analyzed using custom Python code, freely available at https://github.com/VBits/oessa. Statistical analyses were performed using Python or Graphpad Prism v10. Whole brain fos imaging data were registered using Elastix and nuggt (see Methods, Swaney et al 2019, and https://github.com/chunglabmit/nuggt, master branch). Additional analyses on whole brain imaging data were performed using sckit-image v0.19.3 (skimage.feature.blob_log, https://scikit-image.org/) and custom Python code, available at https://gitlab.com/ceda-unibas/sleep-brain-atlas. Code to visualize whole brain data using a web resource is available at: https://gitlab.com/ceda-unibas/bav. The QUINT software pipeline (https://quint-workflow.readthedocs.io/en/latest/index.html) was used to analyze anterograde tracing data. Optimouse software (https://github.com/yorambenshaul/optimouse) was used to quantify behavior in open field test and elevated plus maze assays.

For manuscripts utilizing custom algorithms or software that are central to the research but not yet described in published literature, software must be made available to editors and reviewers. We strongly encourage code deposition in a community repository (e.g. GitHub). See the Nature Portfolio guidelines for submitting code & software for further information.

Data

Policy information about availability of data

All manuscripts must include a data availability statement. This statement should provide the following information, where applicable:

- Accession codes, unique identifiers, or web links for publicly available datasets
- A description of any restrictions on data availability
- For clinical datasets or third party data, please ensure that the statement adheres to our policy

All data will be made public or available upon request. Whole brain atlas data is available at https://sleep-wake-atlas.scicore.unibas.ch/. Data from the Allen Brain Atlas CCFv3 and the Perens et al 2021 LSFM optimized atlas are available at (https://brain-map.org/atlases/anatomy) and (https://github.com/Gubra-ApS), respectively.

Research involving human participants, their data, or biological material

Policy information about studies with human participants or human data. See also policy information about sex, gender (identity/presentation), and sexual orientation and race, ethnicity and racism.

Reporting on sex and gender	n/a
Reporting on race, ethnicity, or other socially relevant groupings	n/a
Population characteristics	n/a
Recruitment	n/a
Ethics oversight	n/a

Note that full information on the approval of the study protocol must also be provided in the manuscript.

Field-specific reporting

Please select the one below that is the best fit for your research. If you are not sure, read the appropriate sections before making your selection.

☒ Life sciences ☐ Behavioural & social sciences ☐ Ecological, evolutionary & environmental sciences

For a reference copy of the document with all sections, see nature.com/documents/nr-reporting-summary-flat.pdf

Life sciences study design

All studies must disclose on these points even when the disclosure is negative.

Sample size	No statistical methods were used to predetermine sample sizes. Suitable sample sizes were estimated based on previous experiments and previously published studies in the mouse neural circuits, neurobiology, and sleep fields.
Data exclusions	Exclusion criteria were pre-established for all virally targeted experiments: animals were excluded if virus expression was 1) undetectable using immunohistochemistry or 2) did not specifically target the region of interest.
Replication	All experiments included at least three replicates (separate animals and/or separate experiment days) and yielded reproducible results.
Randomization	Mice were randomly allocated into experimental groups.
Blinding	Sleep/wake behaviors were automatically classified using computer software and not subject to interpretation by individuals. Parameters for behavioral quantifications remained unchanged between groups.

Reporting for specific materials, systems and methods

We require information from authors about some types of materials, experimental systems and methods used in many studies. Here, indicate whether each material, system or method listed is relevant to your study. If you are not sure if a list item applies to your research, read the appropriate section before selecting a response.

Materials & experimental systems

n/a	Involved in the study
☐	☒ Antibodies
☒	☐ Eukaryotic cell lines
☒	☐ Palaeontology and archaeology
☐	☒ Animals and other organisms
☒	☐ Clinical data
☒	☐ Dual use research of concern
☐	☐ Plants

Methods

n/a	Involved in the study
☒	☐ ChIP-seq
☒	☐ Flow cytometry
☒	☐ MRI-based neuroimaging

Antibodies

Antibodies used

Rabbit anti-Fos (polyclonal, Synaptic Systems 226 003), Rabbit anti-Fos (monoclonal, Synaptic Systems 226 008), Rat anti-Fos (Synaptic Systems 226 017), Chicken anti-GFP (Invitrogen A10262), Rabbit anti-ZsGreen (Takara 632474), Rat anti-RFP (Chromotek 5f8-150), Goat anti-tdTomato (Origene AB8181), Goat anti-5HT (Immunostar 20079), Guinea pig anti-OrexinA-A (Synaptic Systems 389 004), Rabbit anti-Mch (Phoenix, H-070-47), Mouse anti-nNos (Sigma N2280). Primary antibodies were matched with appropriate Donkey or Goat secondary antibodies conjugated to Alexa 488, Alexa 546, Alexa 555, Cy3, Alexa 647, or Cy5 (Thermo Fisher and Jackson ImmunoResearch).

Validation

All antibodies were validated based on comparison with known target expression patterns of the respective targets in mouse brain tissue. Suppliers for the following antibodies note additional validations as summarized below, and additional references are noted as necessary. Rabbit anti-Fos (polyclonal, Synaptic Systems 226 003): human, mouse, rat, dog, cow. Rabbit anti-Fos (monoclonal, Synaptic Systems 226 008): Reacts with: mouse (P01101), rat (P12841), human (P01100). Validated for western blot, immunohistochemistry, immunofluorescence, iDisco, Clarity. Rat anti-Fos (Synaptic Systems 226 017): Reacts with mouse (P01101), rat (P12841), human (P01100). Validated for western blot, immunohistochemistry, immunofluorescence, iDisco, FACS. Chicken anti-GFP (Invitrogen A10262): Validated for western blot, immunohistochemistry, immunofluorescence, immunoprecipitation. Rabbit anti-ZsGreen (Takara 632474): Validated for western blot and immunoprecipitation, and used for immunofluorescence in Mortessagne et al 2024, Scientific Reports, He et al 2018, Nature Protocols. Rat anti-RFP (Chromotek 5f8-150): Validated for immunofluorescence and ELISA. Recognizes RFP variants including mOrange, DsRed, mCherry, mRFP, mRFPRuby, mScarlet, tdTomato, mKate2, mPlum. Goat anti-tdTomato (Origene AB8181): Validated for western blot, immunohistochemistry, immunofluorescence. “In 293HEK cells transfected with cds plasmid detects a band of 55 kDa by Western blot. It also detects tdTomato in brain sections by IHC. This antibody (AB8181) is specific for tdTomato and mCherry proteins. It does not cross-react to GFP (green fluorescent protein).” Goat anti-5HT (Immunostar 20079): Reacts with Cat, Cod, Cow, Crayfish, Dolphin, Fish, Frog (Xenopus Laevis), Guinea Pig, Hamster, Human, Mexican salamander (Ambystoma Mexicanum), Monkey, Moth, Mouse, Pig, Rabbit, Ram (Sheep), Rat, Salamander, Snail, Tapeworm, Worm, Zebrafish. Validated for immunohistochemistry and immunofluorescence. Guinea pig anti-OrexinA (Synaptic Systems 389 004): Validated for immunohistochemistry, immunofluorescence. “Recognizes Orexin A with only minor cross-reactivity to the unprocessed precursor protein. Does not cross-react to Orexin B. Knockout validated.” Rabbit anti-Mch (Phoenix, H-070-47): Validated in brain sections in numerous publications, for example del Cid-Pellitero & Jones 2012, J. Neurosci. Mouse anti-nNos (Sigma N2280): Validated in comparison to different nNos antibodies by Paul et al 2018, eNeuro.

Animals and other research organisms

Policy information about studies involving animals; ARRIVE guidelines recommended for reporting animal research, and Sex and Gender in Research

Laboratory animals

The following mouse strains were used, and are listed with JAX strain numbers: C57BL/6J (Strain#000664), (Fos-2A-iCreERT2, Strain #030323), Ai14 Cre reporter (RCL-tdT, Strain #007914), Sert-Cre (Slc6a4tm1(cre)Xz, Strain #014554), Vgat-IRES-Cre (Slc32a1tm2(cre)Lowl , Strain #028862), and Vglut2-IRES-Cre (Slc17a6tm2(cre)Lowl, Strain #016963). Mice were maintained on a 12 h:12 h light:dark cycle with ad libitum access to food and water, at 22±2C and 55±10% humidity. Experimental animals were used between 2-6 months of age.

Wild animals

No wild animals were used in this study.

Reporting on sex

Male C57BL6/J animals were used for whole brain Fos mapping during circadian and sleep-deprivation timepoints, while both males and females were used for all other experiments.

Field-collected samples

No field-collected samples were used in this study.

Ethics oversight

All experiments were performed in accordance with Swiss National Institutional Guidelines on Animal Experimentation and were approved by the cantonal Veterinary Office Committees for Animal Experimentation.

Note that full information on the approval of the study protocol must also be provided in the manuscript.

Plants

Seed stocks	n/a
Novel plant genotypes	n/a
Authentication	n/a
