# Supplementary material for: Wake-activated neuronal populations that regulate sleep drive
Source: Nature. 2026 Aug 19;658(8137):987–97. doi: 10.1038/s41586-026-10928-3 (PMC13686944; doi:10.1038/s41586-026-10928-3)
Supplement: Supplementary file 2 [file 41586_2026_10928_MOESM2_ESM.pdf]

nature portfolio

Peer Review File

Wake-activated neuronal populations that regulate sleep drive

Corresponding Author: Dr Alexander Schier

Any redactions in this file are there to maintain patient confidentiality, the confidentiality of unpublished data, or to remove third-party material.

This file contains all reviewer reports in order by version, followed by all author rebuttals in order by version.

Attachments originally included by the reviewers as part of their assessment can be found at the end of this file.

Version 0:

Reviewer comments:

Referee #1

(Remarks to the Author)
Sleep homeostasis (catching up on lost sleep) is not a fully understood process. So, identifying how this works precisely could give insights into the function of sleep. Here, Joo et al investigate the circuits, rather than the molecules, that could regulate sleep homeostasis in mice. They find several neuronal types that regulate this, in addition to the ones already in the literature. The manuscript is well written and beautifully presented; I think it is a lovely paper; however, I do not see what is fundamentally new or step-changing about this work (that means it should be published in Nature). The approaches used are fairly standard (cFOS trapping, cell reactivation; potassium channel inhibition, EEG analysis). The final result in the paper is the most impressive one, that co-inhibition of GABA and serotonergic cells substantially reduces sleep amount (I really like Fig. 6b!) without influencing sleep homeostasis. As a phenomenon this is intriguing, but then it is not any different from modafinil or some other drugs that promote extended wakefulness without influencing sleep homeostasis.
I think a key thing missing from the manuscript would be a mechanism for how these “sleep homeostasis” cells get activated; indeed, the authors themselves write at the end of their paper that their results provide “entry points to dissect the molecular mechanisms that ultimately make sleep inescapable”. This is very well put, and I feel this is what is needed now (from all of us) to move the field forward, rather than further circuit descriptions.
In the text, the authors write that “induction of the immediate early gene Fos can label activated neurons that control homeostatic behaviours such as feeding or drinking” (line 46). At the same time, the authors give the, I am sure unintentional, impression that this method has not been used to study sleep homeostasis. Yet cFos-trapping and cell reactivation was first used to identify preoptic hypothalamic neurons involved in mouse sleep homeostasis (and temperature regulation) ten years ago (ref. 30, Zhang et al 2015). Since then, a whole range of neuronal types have been identified, not all mentioned by Joo et al., as participating in mouse sleep homeostasis (for example, preoptic galanin neurons (ref. 32 Ma et al, but also galanin neurons in zebra fish, Reichert et al 2019, Neuron), VTA GABA neurons (Yu X et al 2021,Mol Psychiatry, PMID: 32555422), prefrontal cortex somatostatin neurons identified by cFos trapping, Tossell K et al 2024 Nat Neurosci) that contribute to catching up on lost sleep; in fact, I think it is quite likely that the homeostasis network will be distributed, and that these “homeostasis cells” will just be the same cells as those that induce sleep without sleep deprivation.
Specific points:
Major:
1. Although using immediate early genes like c-fos is a useful and accessible approach for observing and quantifying neuronal activation, it lacks high temporal resolution due to the time required for c-Fos protein synthesis following c-fos gene expression. Since immunostaining was used to assess activity changes, the measured density at each time point does not represent real-time activity but rather reflects expression levels approximately 1–1.5 hours prior. For example, the c-Fos density at 1.5h into the recovery sleep in Figure 1 indicates the neuronal activity at the end of the 6h sleep deprivation. This would suggest that the activity of Type 3 neurons has already started to decline before the recovery sleep was initiated. This pattern is similar to that of Type 1 group but only with greater intensity.
2. The authors should show in vivo whether Type 3 (MR and aMPO) neurons reflect the duration of wakefulness to support their hypothesis. Does the activity of these neurons increase as sleep loss accumulates? Are they more active at the end of

the dark period compared to that at the beginning of the light period?
3. The statement in Line 73 - "…Type 3 regions do not selectively respond to stress or aversive stimuli." is not supported by experimental data. The process of sleep deprivation itself is aversive and the fact that the activity of these neurons is higher during sleep deprivation than that in the baseline undisturbed wakefulness suggests the opposite. Type 3 neurons could be responding and essential for maintaining high level of arousal caused by external aversive stimuli.
4. It is unclear when wake-active MR/aMPO neurons promote sleep when activated. Do they detect sleep loss and subsequently trigger sleep at a threshold? In the undisturbed condition, MR neurons become less active during sleep, suggesting their activity is not required for normal sleep. Why do they induce sleep upon activation? (refer to Figure 2). Also during sleep deprivation, these neurons show high activities but do not immediately induce sleep. Similarly, artificially activating them do not necessarily induce sleep (refer to Figure 2). How to interpret this discrepancy?
5. In Figure 3i, sleep duration does not seem to change in light phase. If the sleep deprivation was performed using the same protocol at the beginning of the light phase, what cause the changes in Figure 3m and n? Does the delta power change in during the light phase when inhibiting aMPO?
6. In Figure 3 and 4, the authors specifically targeted MR or aMPO neurons that are selectively active during sleep deprivation, which typically represent a small subset of neurons. In contrast, in Figure 5 and 6, the authors manipulated entire populations of GABAergic, serotonergic and glutamatergic neurons, including many that do not respond to sleep deprivation. This suggests that the sleep-promoting effect is a general property of certain MR populations without interfering with sleep homeostasis. Notably, this is similar to previously identified serotonergic neurons in the dorsal raphe and GABAergic neurons in the VTA, which are wake-active but can generate NREM sleep upon activation.

Minor:
1. In figure 2 and 5, the effect of CNO appear to be longer than the time points shown on the x-axis. It would be better to present the results over a longer duration e.g. 12h to capture the dynamics of these changes.
2. Figure 2, n=3 for recovery TRAP which is low.
3. In Figure 3, it would be better to represent the changes of baseline sleep in light and dark phases separately.
4. In figure 4f, no presentative images shown.

Referee #2

(Remarks to the Author)
In their report the authors use multiple methodologies to demonstrate that a category of wake-active neurons regulate sleep drive. Below I comment their findings following the paper organization.
I see some artefactual staining on the tissue surrounding the ventricules in their illustrations. In these regions, Fig 1C, some Type 3 neurons in particular are localized in these areas. I wonder whether the authors used a way to remove artefact counting?
The cFos staining shown in Extended Fig. 2 does not look in focus and for some images neurons have strange morphology with vertical lines extending from them? Is it due to the clearing process?
For some structures, like the first photo on the left of VTA it is really out of focus and the neurons shown don't seem to be inside the VTA but more the interpeduncular nucleus (IP)?
For S1 there is a strong labeling in layer 1 particularly after 3h of deprivation. This is quite unusual and the size of the neurons seems big. Did the authors verified that it corresponds to neurons?

The authors state that to control for stimulus-specific effects, they compared these regimes to sleep deprivation during the dark phase. What they mean by stimulus-specific effects? Like the method for depriving? However, it might be that the differences are due to the fact that wakefulness occurring during the night is not inducing the same cFos staining that during the day. In addition, circadian time could induce also other neuronal activation.
The authors state that they used voxel-wise clustering of Fos density across experimental timepoints to reveal three types of subtypes. I assume that it corresponds to three subtypes of structures although it is not clearly mentioned at that point of the text. What is not clear in the text, in the Figures including Fig 1 and extended Fig. 2-3 is how they did discriminate these regions? There is indeed no statistical tests shown in the text, the figures or the method section.
Because of that lack I'm not sure what means the segregation between the different types. Further, it is well know that for nearly all structures there is an overlap of wake and sleep-active neurons. How they deal with such question?
My question also relates to the illustration Fig 1C showing type 3 cells in the ventricles or in white matter.
I'm also a bit skeptical on the neuroanatomical landmarks used in Fig. 1. Indeed there is a mixture of classical structures:
DG, dentate gyrus; MR, median raphe; VTA, ventral tegmental area;
With some subdivisions of structures never reported before to my knowledge:
VMHdm, ventromedial hypothalamus, dorsomedial portion;
aMPO, anterior medial preoptic area
LHAdl, lateral hypothalamic area dorsolateral portion
Why the authors made such subdivisions? For example for the LHAdl, where is it located? It could be the zona incertain in fact? And the LHA is a very extensive structure, is it rostral or caudal LHA?
One important denomination is the aMPO. I'm not sure it is not misleading name since from their Extended Fig. 10 it rather seems to correspond to the Median preoptic nucleus as stated by the Allen Atlas.
Finally they used quite vague denominations for other structures like:
pHbMB, peri-habenular midbrain;
PTh, posterior thalamus;
There are known structures in these regions. Why they dont use classical terminology? The peri-habenular midbrain is an interesting name but I have no idea where it is located.

In summary, the whole brain analysis made is impressive since it is done for the first time with so many time points both in control conditions, during sleep deprivation and sleep recovery. However, the description of the results is not accurate and complete and lack statistical comparison.
In the text, the authors used the term voxel but in the Fig1. Legend they wrote cFos density. Is it really a density meaning that they divided the number of cFos+ neurons by the surface of the structures? It seems not since it is based on voxel analysis. May be this is the reason why they found in some cases that only subparts of a structure are activated. I'm not sure this is the way to go since it highly depends on the alignment of each brain with the atlas. It is certainly different between brains and therefore very difficult to be sure all voxels are exactly in the same subpart of a structure. It would make more sense to compare a mean of the density in a whole classical structure defined by the atlas.
Now about the different types of structures they identified. They report that Type 1 responses peaked 1.5-3h into sleep deprivation and decayed thereafter and that they accounted for the greatest proportion of the brain and were enriched in cortical areas. I understand that it corresponds to neurons active during wake. How they explain that the labeling of these structures decrease over time during sleep deprivation? If there are active during wake they should be cFos+ during all the sleep deprivation period and even a bit after since cFos expression is visible normally one hour after stimulation? In fact, I expect that Type 3 neurons are also containing wake active structures? Here again, I would like to see the statistical tests showing that Type 1 and Type 3 are really behaving differently.
In fact, I would like to see a supplementary Table with all structures analyzed. What's happening for example in the Dorsal raphe, the tuberomammillary nucleus and the locus coeruleus, three classical wake systems?
Indeed Fig 1 is not very informative and if Type 1 structures are quite big, the aspect of Type 2 and Type 3 structures is really puzzling. For example you have multiple Type 3 spots inside the caudate nucleus. This is really strange. Indeed, it seems impossible that the analysis made in multiple mice and conditions can lead to such spotty positive spots. The dentate gyrus and the ventral medulla are Type 3 structure. I wonder whether the authors did differentiate NREM and REM sleep in their analysis? It would be important to know whether there is a strong REM or nonREM increase in their recovery period?
When you report line 74 that Type 3 structures activation is correlated with wake duration, I see no statistical analysis there. This is a crucial point since it is the basis of the demonstration.
The same for Type 2 structures and the link with sleep, I don't see the statistics
I'm also surprised to see the LHAdl and VTA are type 3 structures. They indeed contain wake-system which rather inactive during NREM and orexin and dopamine neurons of the VTA are not known to increase their activity with sleep pressure?
Concerning the TRAP experiment, the authors state that the control condition is a rested control (line 405) with injection in undisturbed animals in the light phase but in Fig. 2 , they state recovery TRAP?
Also the number of mice with control TRAP injection is only of three. This is not sufficient for such experiments.
I'm also lacking the statistical differences in the hour by hour figure 2e, k and in all the other figures. I don't get what happens with REM? It is not modified? I would like to see two hypnograms showing the control and a deprived TRAP stimulation mice to have an idea of the distribution of each state.
Fig. 2i, the box is rather centered on the nucleus accumbens than the MPO. It is not clear what area the photo is showing since there is no landmark.
Interestingly, the authors also studied the effect of LHA stimulation. Strangely here that don't specify that it is the dorsolateral portion of it although their square in Extended Fig. 5a is dorsal in the HLA. Anyway in B the photo show a quite extensive transfection across the entire HLA. Further, the orexin neurons seem transfected but not the MCH ones. They then stimulated the TRAPed neurons and obtained wakefulness and concluded that some Type 3 structures are not regulating sleep but wake and Type 1 neurons as I indeed discussed above. My question here is whether it is because there are not selective to the dorsolateral portion or that the nomenclature is in fact covering Type 1 neurons?
For the Kir experiments, I dont see in the Fig3 the significance related to line 127. Overall, the experiments quite convincingly show that MR and MPO might play a role in sleep drive. However I'm missing more detailed analysis with the hM4Di experiments since the effect on sleep should be more pronounced that after the permanent inactivation using Kir. We know that CNO effect last 6h so I wonder what would happen if they inject CNO at the end of the deprivation? This would directly prove the involvement of the median raphe and these experiments are necessary also for the MPO.
The authors then look at the efferents and afferents of the TRAPed neurons of the median raphe. Then, they stimulate the TRAPed neurons and show that some of the structures targeted by the median raphe contained a larger number of cFos and others less suggesting that both excitatory and inhibitory neurons compose the TRAPed neurons. Since they show that some median raphe neurons are GABA and others are serotonin, do they believe that serotonin neurons are excitatory?
They should use LHb for lateral habenula for clarity. It is also a bit surprising that they found a decrease in cFos in the VI since it is a oculomotor nucleus. They should confirm that it is not a nearby structure.
Finally, they show that chemogenetic activation of the TRAPed neurons projecting to the lateral preoptic area induces an increase in NREM suggesting a role for such pathway in the induction of NREM by the median raphe.
Then the authors made triple staining using vGAT, vGlut2 and serotonin to determine the neurotransmitter content of the median raphe TRAPed cells and demonstrated that the cells are mostly serotonin or vGAT but not vGlut2 positive. They don't determine whether serotonin neurons are also GABAergic but it seems it has been shown in a previous paper and they should specify it if it is indeed the case.
Then, the authors used vgat-cre, vglut2-cre and Sert-Cre lines to stimulate and inhibit by chemogenetic the three populations of median raphe neurons. They nicely show that serotonergic and GABAergic neurons induce the same effect that the TRAPed neurons indicating that these two populations are responsible for promoting sleep after sleep deprivation. In contrast, glutamatergic neurons are responsible for inducing wakefulness.
The more convincing result might be their last experiment in which they used Kir to inhibit both the serotonergic and GABAergic neurons of the median raphe chronically. They indeed observed a 70% decrease of NREM sleep compared to control and increased mean wake bout length over 2-fold and this during nearly one month. Further, delta power increased is reduced after wake periods.
It would be really interesting to look at the effects on behavior of such long lasting insomnia. By the way, the authors did not discuss the possibility that median raphe neurons dysregulation could play a role in insomnia and they should I believe.

In summary, the results presented in the present paper are quite impressive and identify a full new target for sleep induction of a different kind that previously identified systems.
As described above I have some criticisms in particular concerning the neuroanatomical analysis. Although it constitutes a huge work the way it is analyzed and presented is not optimal and even the interpretation might not be accurate since most regions contain mixed populations. The median raphe is even a good example since it contains neurons with opposite role although they seem to be all active during wakefulness. Also missing from the paper is recording of the median raphe neurons to show that their activity is different than that of classical wake systems.
I have also some concern on the identification of the median preoptic area and even of its definition and this should be corrected.
Overall despite these concerns I believe the report is of great interest for understanding how sleep is regulated.

Referee #3

(Remarks to the Author)
The manuscript by Joo and colleagues investigates the neural circuitry underlying sleep drive after prolonged wakefulness. Prior work has identified direct sleep state regulation circuits, and the neural drivers of sleep pressure are still not well understood. This manuscript demonstrates that specific cell types within the median raphe have distinct and substantial effects on sleep/wake behavior, and specifically the buildup of sleep pressure during wakefulness. This is an exciting topic and the results are interesting and well-written. There are some questions about the data and analyses. The whole brain analysis in particular is an important part of the results but has weaknesses that prevent interpretation of the results.

- the Fos data in Fig 1 are interesting and a key part of the paper, but represent a qualitative rather than quantitative assessment of activity, and it is not currently possible to assess which parts of these results are robust. The way the data are analyzed is statistically circular, where clustering is performed and then the difference between the clusters is displayed, which can produce misleading results. It's therefore not clear which specific brain areas are showing real effects vs. noise. To draw a rigorous conclusion in Fig 1., the distinct spatiotemporal patterns would need to be quantitatively compared, and statistically testing data that are already separated by clustering is problematic unless a statistically independent method is used to define the clusters.

- the methods for the whole-brain analysis need more detail to enable replication. How large was the blurring kernel? Where were the manually selected index voxels and how were they chosen? An analysis to determine whether a region's inclusion/weighting in a given cluster is statistically robust is needed.

- Fig 1e states 'Representative data for 8 animals' - how were these data selected? What do the error shaded bars represent? It would be better to show all the data with full statistics.

- It is not completely clear in Fig 2, but it seems there is no control with mice receiving CNO without cell-type-specific activation. CNO can directly induce sedative-like effects and could be expected to increase NREM sleep behavior, and a control statistically comparing matched animals receiving CNO and without the Deprivation/Recovery-TRAP is not shown. Extended data Fig. 4d suggests this non-specific CNO effect could be present since the rested mice also show a trend towards more NREM sleep with CNO; to use this condition as a control, the difference between different CNO groups would need to be reported, but the statistical comparison between the rested and sleep-deprived conditions is missing (Niewenhuis et al., Nature Neuro, 2011)

- n animals missing from some figures; e.g. Fig. 3o,p;

- The control data in Fig. 5m vs Fig. 5g show very different NREM behavior patterns (top panels); the difference in the control groups is as large as the stated effect size of the CNO. Why are these groups so different, is this reflecting that activation vs. inhibition experiments were done in different phases (light/dark) for different cell types? If so why were the manipulations switched in phase across experiments, given that the effects of each cell type were not known in advance?

- what is the n in Fig. 6h, how many mice did these bouts come from? Why are there so many fewer wake bouts from the Kir2.1 mice even though Fig. 6d shows they are awake more than controls? Were data excluded prior to this calculation?

- The discussion states the MR cell-type-specific effects are the key regulator of sleep drive, but based on Fig. 1 it seems unclear that it's justified to state that sleep drive is specific to this one region; many areas are flagged and it seems also plausible that this is a systems-level process of which MR is one important part, but which doesn't necessarily have only one area as a single switch. While inhibiting MR is proposed to specifically reduce sleep drive and is contrasted with areas that cause animals to be 'excessively sleepy' in the discussion, and it's suggested that these kir2.1 animals are not sleep-deprived, whether this manipulation actually removes sleepiness from a cognitive/functional perspective is not part of this paper. This does not require new experiments but could be considered in the discussion.

Version 1:

Reviewer comments:

Referee #1

(Remarks to the Author)
The authors have substantially strengthened the manuscript by adding an extensive whole brain data set in the form of an online sleep–wake atlas, which will constitute a valuable community resource. They further show in new experiments that, although the animals with tonically inhibited GABA and 5HT cells exhibit markedly reduced sleep, this sleep loss does not measurably impair their cognitive performance, while prolonged sleep deprivation is associated with approximately 17% lethality (extended data Fig. 14e), perhaps underscoring the physiological cost of sustained sleep loss.

Figure 6, documenting the prolonged genetic sleep deprivation paradigm, remains particularly striking and compelling.

The new electrophysiological data (Fig. 5p,q) are also informative: they demonstrate that MR GABAergic neurons become progressively more excitable with ongoing sleep deprivation, providing additional support for the idea that sleep drive is encoded by plasticity-based mechanisms. This is an emerging new idea in the field.

The authors have thoroughly and convincingly addressed all my previous concerns. Overall, this is an elegant and comprehensive study that will be of broad interest to researchers investigating the regulation and functional significance of sleep.

Happy to sign as: William Wisden

Referee #2

(Remarks to the Author)
Overall, the number of experiments made is amazing and it indeed seems that the median raphe GABAergic neurons plays a role in sleep induction. In a way, there are two many experiments shown to make such demonstration. It is therefore difficult to assimilate all of them. The will to provide a huge number of experiments to show that the study is going in the right direction is rather deserving it. Of course, the neuroanatomical data is crucial as well as the chemogenetic and Kir experiments but other ones are not that necessary.
One key experiment is on the other hand still missing: the recordings of the Median raphe neurons. I'm indeed not convinced by the « in vitro » recordings. Even fiber photometry recordings which are way simpler would be more convincing. Of course, the best would be to provide unit recordings and to show the behavior of the Median raphe GABA and serotonin neurons across sleep-wake states.

Below, my other concerns remaining in particular with regard to the cFos experiments.

Concerning the artifactual labeling near the ventricule, the new images are indeed very nice and indeed showing that there are a few cFos labeled cells near the ventricules. However, I'm not convinced at all that it corresponds to their staining in all their voxel figures like Fig. R2 for the recovery condition. Indeed, it seems for example that the magenta above the lateral ventricule is in the white matter. It is well known that cFos immunostaining whatever the methods used give rise to artifactual labeling. To eliminate such artefactual labeling the authors would need to use a more classical method than the one they use. Classically, automatic counting is done for each cFos labeled cells using deep learning based on the form factor and the size of the cells. What I don't buy in their analysis is that instead that use voxel analysis which is not strictly corresponding to the cells. By this mean, they loose the power of the method which is giving a cell resolution of the staining. Further, they cannot eliminate artifactual labeling.
Then, they state that:
« voxel-based quantifications more faithfully capture the data and are more suited to isolate sleep or wake-related Fos signals than quantifications across entire Allen Brain structures. »
This sentence is interesting but wrong. The main advantage of the cFos method is that it is showing activated neurons at the cellular level. By this means, it is vastly superior to classical imaging. Therefore, to remove such accuracy with voxeling is a mistake. I agree with the authors that using just the Allen Brain Atlas is not enough since it is a very simplified atlas. But the best way is to look at the cFos labeling and to add accurate real subdivisions based on previous publications like those mentioned by the authors. Such method also allow to make corrections of the alignement between mice which always occur when making such study. The brains of different mice is indeed never fully aligned automatically. I personally dislike Fig. R10 since it looks like functional imaging and gives no ideas of the real density of the cFos cells. Please add to this figure, a photomicrograph showing the cFos labeled cells.
I'm also not convinced by their explanation that the decrease of cFos staining after 3h of deprivation is due to the fact that sensory-motor systems are less activated. Indeed, the longer you sleep-deprive the more you need to simulate the animals to keep them awake. In addition, across time different sensory-motor neurons are recruited in the cortex since the experience is always different and they introduce new objects and therefore induce the activation of different neurons over time.
I'm also not convinced by their answer concerning spotty labeling in the caudate nucleus. When you align many brain, it is impossible that it is so perfect you can see such very localized activation across animals. The authors need to verify their cFos staining to understand why they got this spotty labeling. I don't like their answer there since they simply did not control this point.
« In contrast, "CNO injection at the end of deprivation" would overlap with the naturally occurring decline in activation during the recovery period, thus making it difficult to interpret the results. »
This is a purely speculative statement if they did not test it.

« As for VI, both Figure 4 and the additional data below demonstrate MR projections to VI, as well as suppression of Fos in VI. We now include this in an extended data figure. »
This is a puzzling results, how the authors interpret these results? It cannot be a pathway implicated in wake or sleep control. Is it known that the serotonin neurons of the MnPo project to the VI? Again their voxel image is not convincing to me. They should look at the neurons and confirm that the cFos-labeled neurons are inside the VI only composed of motoneurons which are quite big.
« the MR includes three non-overlapping cell types with distinct neurotransmitter identities. GABAergic Vgat+ cells represent 60% of MR cells, while glutamatergic Vglut2+ cells and serotonergic cells respectively comprise ~25% and ~10%." We concurrently cite the two relevant references: Sos et al 2017 and Szonyi et al 2019. We hope this provides additional clarity. »
In fact, it has been reported that 5-HT neurons of the MR express vGlut3 (Ren et al., 2019). This might explain why they show many activated regions when they stimulated these neurons.
I realized based on their answer to reviewer 3 that EEG and EMG recordings have not been done in all mice in particular in the cFos experiments. They need to clearly state which mice were recorded in the paper since it is a crucial information. Indeed without sleep recordings how they can be sure that the mice were not sleeping or on the contrary awake?
How the authors explain that in Fig 1.d they don't show high activation in the cortex during the night since Wake is predominant at that time?
Extended data 2 figure, I'm still not convinced by that figure. The morphology of cFos labeled cells is not clear cut and should be. It seems there is a high background. The delimitations of the structures is also not very convincing in particular for aMPO. It is needed to show the bottom of the brain to localize the nucleus.
Extended Data Fig. 3a, their LHA seems to include the zona incerta. Did they look whether it is not rather zona incerta since there is a recent paper showing that it also contain W-active sleep inducing neurons?
I finally wonder how the authors explain that the Kir mice freeze less during the shocks (FigR5)

In summary, the demonstration that the median raphe contains neurons active during wake involved in sleep induction is new and interesting. However, there are still problems in the data shown and the manuscript could be streamlined. The addition of « in vivo » unit recordings is key to my point of view and is still lacking.

Referee #3

(Remarks to the Author)
The authors have addressed my comments and I congratulate them on the interesting and well-written paper, which elucidates the neural mechanisms underlying sleep drive.

One suggestion is that the authors could consider rephrasing the statements in the discussion that co-inhibition does not produce behavioral deficits, or perhaps adding context to this statement somewhere in the discussion. The statement gives the impression that manipulating these cells can fully remove the need to sleep, but since 17% of the animals receiving this manipulation died, it does cause behavioral deficits. It seems plausible that only the more cognitively robust animals survived to be tested on the behavioral tasks, which may bias the behavioral results somewhat. However this is just a discussion point for the authors to consider.

Version 2:

Reviewer comments:

Referee #2

(Remarks to the Author)
I would like to congratulate again the authors for their discovery that MR GABA and to a lesser extent serotonergic neurons play a role in sleep induction although they are mostly activated during sleep deprivation. This is an original concept complementing recent discoveries on the reuniens and zona incerta neurons with closed properties.
I see that I will have to wait to see the recordings of the MR serotonin and GABA neurons. I understand this is a bit demanding but it would have strongly supported their demonstration.
I would like also to thank the authors for their explanations on the specificity of their analysis of cFos labeled cells. I also would like to thank them for their review of papers using the same voxels approach with great success. This discussion is of great interest and importance for me since I have been using cFos for decades and I'm happy that the authors used it successfully again meaning that this old method is still bringing amazing results. I'm just reluctant on the downsampling of the labeling obtained made in their study and others. I don't believe this is the best way to go although I buy their results. By the way, I see the MR neurons in our own sleep deprivation experiments. I therefore fully support the results obtained. I nevertheless believe that looking at individual cells is a better approach since in most nuclei, neurons responsible for NREM, REM and Wake are intermingled and therefore a voxel approach cannot identify a structure with such heterogeneity.
In conclusion, I would like to congratulate the authors for their discovery. I hope it will open a new path of research on the mechanisms responsible for inducing sleep complementing previous hypothesis on the inhibitory interactions between sleep and wake systems.

Open Access This Peer Review File is licensed under a Creative Commons Attribution 4.0 International License, which permits use, sharing, adaptation, distribution and reproduction in any medium or format, as long as you give appropriate credit to the original author(s) and the source, provide a link to the Creative Commons license, and indicate if changes were made.
In cases where reviewers are anonymous, credit should be given to 'Anonymous Referee' and the source.
The images or other third party material in this Peer Review File are included in the article's Creative Commons license, unless indicated otherwise in a credit line to the material. If material is not included in the article's Creative Commons license and your intended use is not permitted by statutory regulation or exceeds the permitted use, you will need to obtain permission directly from the copyright holder.
To view a copy of this license, visit https://creativecommons.org/licenses/by/4.0/

Response to Reviewer Comments

We thank each referee for their thoughtful feedback and their enthusiasm for our manuscript. The referees describe our approach and findings as "impressive," "beautifully presented…a lovely paper" (Referee 1); "impressive and [identifying] a new target for sleep induction," and "of great interest for understanding how sleep is regulated (Referee 2); and "well-written," "substantial," and "interesting" (Referee 3).

In our previous submission, we used comprehensive whole-brain activity mapping and circuit manipulations to identify key regions that regulate mammalian sleep drive. Specifically, we 1) comprehensively mapped brain-wide activity signatures that reflect both spontaneous and experimentally induced changes in sleep drive, 2) identified novel neuronal populations in the median raphe and hypothalamus that are necessary and sufficient to generate increased sleep drive. Our study culminated with the remarkable observation that inhibiting specific neurons in the median raphe reduces sleep by ~70% without increases in the urge to sleep.

Our revised manuscript has been substantially improved based on referee suggestions: We now include an online atlas of our whole-brain mapping results, which allows users to browse activation patterns across circadian or deprivation timepoints. Furthermore, we now include new behavior and electrophysiology experiments that provide novel mechanistic insights. Accordingly, our main and supplementary figures have been extensively revised and expanded, and the manuscript includes 8 additional extended data figures.

We first summarize these major improvements, before providing point-by-point responses to all referee suggestions below.

Notes: For readability, figures are provided in-line with the text and numbered as Figures R1-R11. Where applicable, the corresponding figure numbers for the paper main text are indicated in the legends. References are listed at the end of this document. In the paper main text, significant changes from the initial submission are highlighted in yellow.

<u>1. Whole brain data analysis and presentation</u>: At the beginning of our manuscript, we map brain-wide activity signatures that reflect both spontaneous and experimentally induced changes in sleep drive. Based on referee comments, we assessed the robustness of our approach using two new analyses: 1) Based on comments from Referee 3, we re-analyzed our data using less biased clustering approaches (see Methods and Figure R1). In our initial submission, our strategy relied on manual selection of brain regions with circadian, wake, or sleep-correlated patterns, followed by clustering and label propagation to the rest of the brain. In our updated approach, we first automatically segment all voxels within each experiment according to peak activation timepoints, followed by hierarchical clustering into distinct response types (Figure R1 below). This analysis likewise allowed us to segment the brain according to wake or sleep-correlated response patterns, and reached identical conclusions as our previous approach. 2) Based on comments from Referee 2, we added statistical analyses that further distinguish the responses of individual anatomical regions (Fig 1). The Methods section reflects these changes, and we elaborate in greater detail in the point-by-point responses below.

Furthermore, based on questions from Referee 2, we created a publicly available atlas that allows users to browse or query our whole-brain Fos data (https://sleep-wake-atlas.scicore.unibas.ch/ - See Figure R2 below). Specifically, the atlas website allows users to:

1) Examine Fos responses over time for all experiments
2) Query Fos responses by brain regions of interest
3) Query Fos responses by temporal patterns of interest
4) Examine hierarchical clustering of Fos response profiles

Together with our revisions, we believe the website addresses many of the referees' neuroanatomy questions (eg: How do regions not directly analyzed or discussed in the paper respond during sleep deprivation?). It will also serve as a useful resource for the neuroscience community.

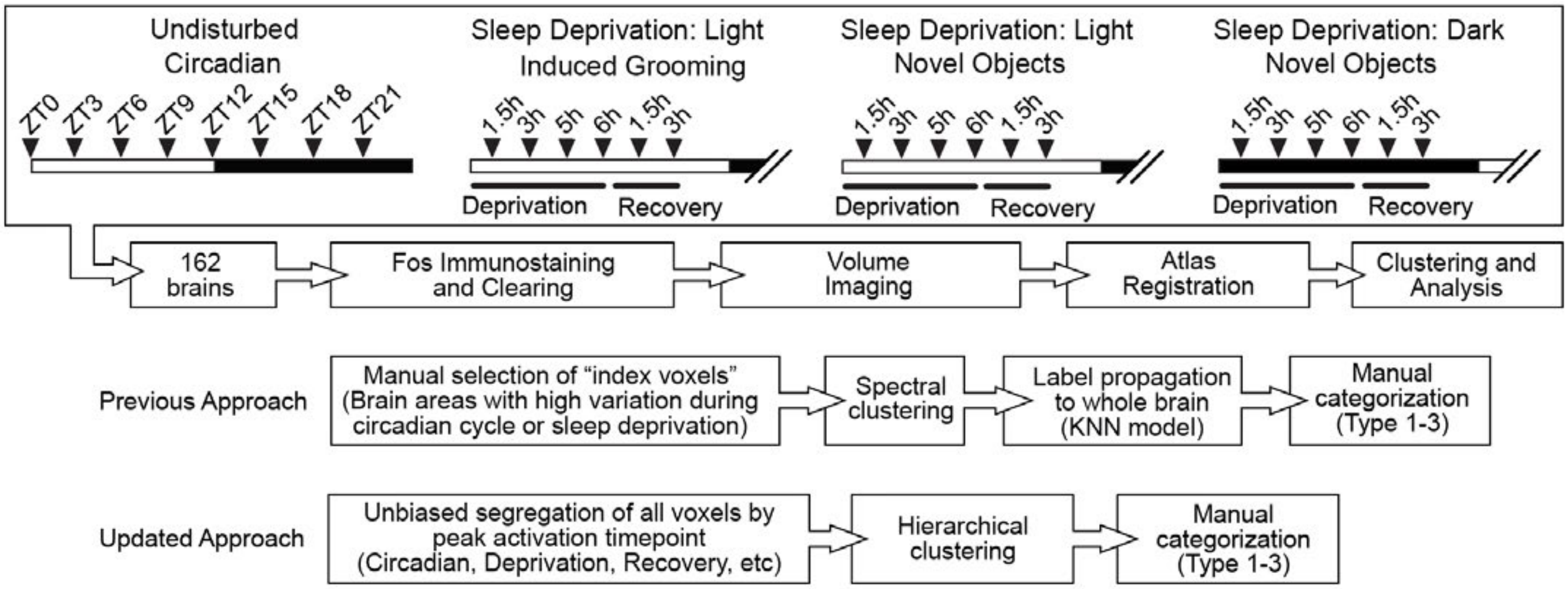

Figure R1: Whole brain activity mapping approaches.
Top: Summary of timepoints for whole brain Fos immunostaining. Middle: Previous clustering approach, which relied on manual selection of brain regions with circadian, wake, or sleep-correlated patterns, followed by clustering and label propagation to the rest of the brain. Bottom: Updated clustering approach. Within each experiment, all voxels were automatically segregated according to peak activation timepoints, followed by hierarchical clustering.

53

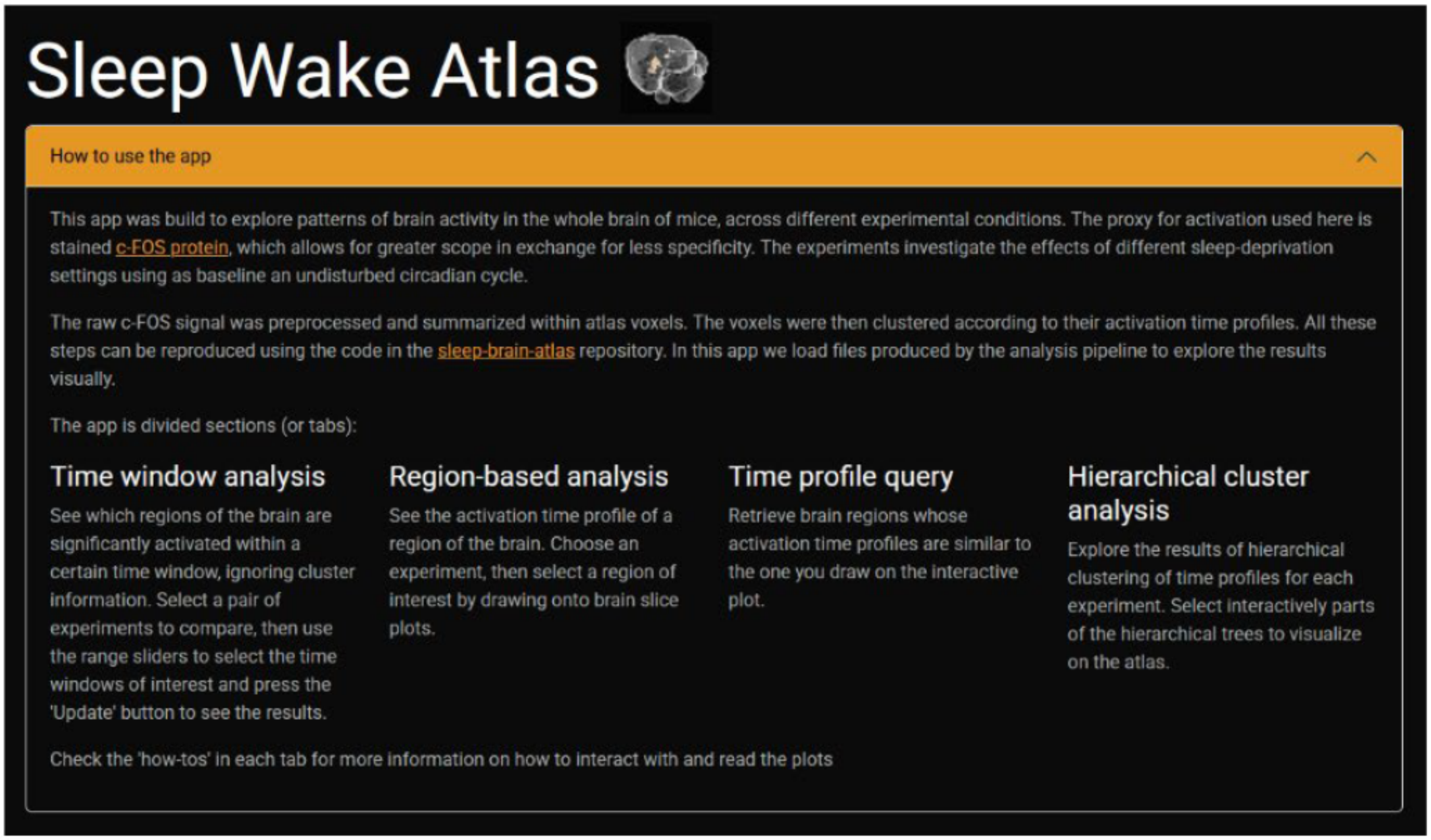

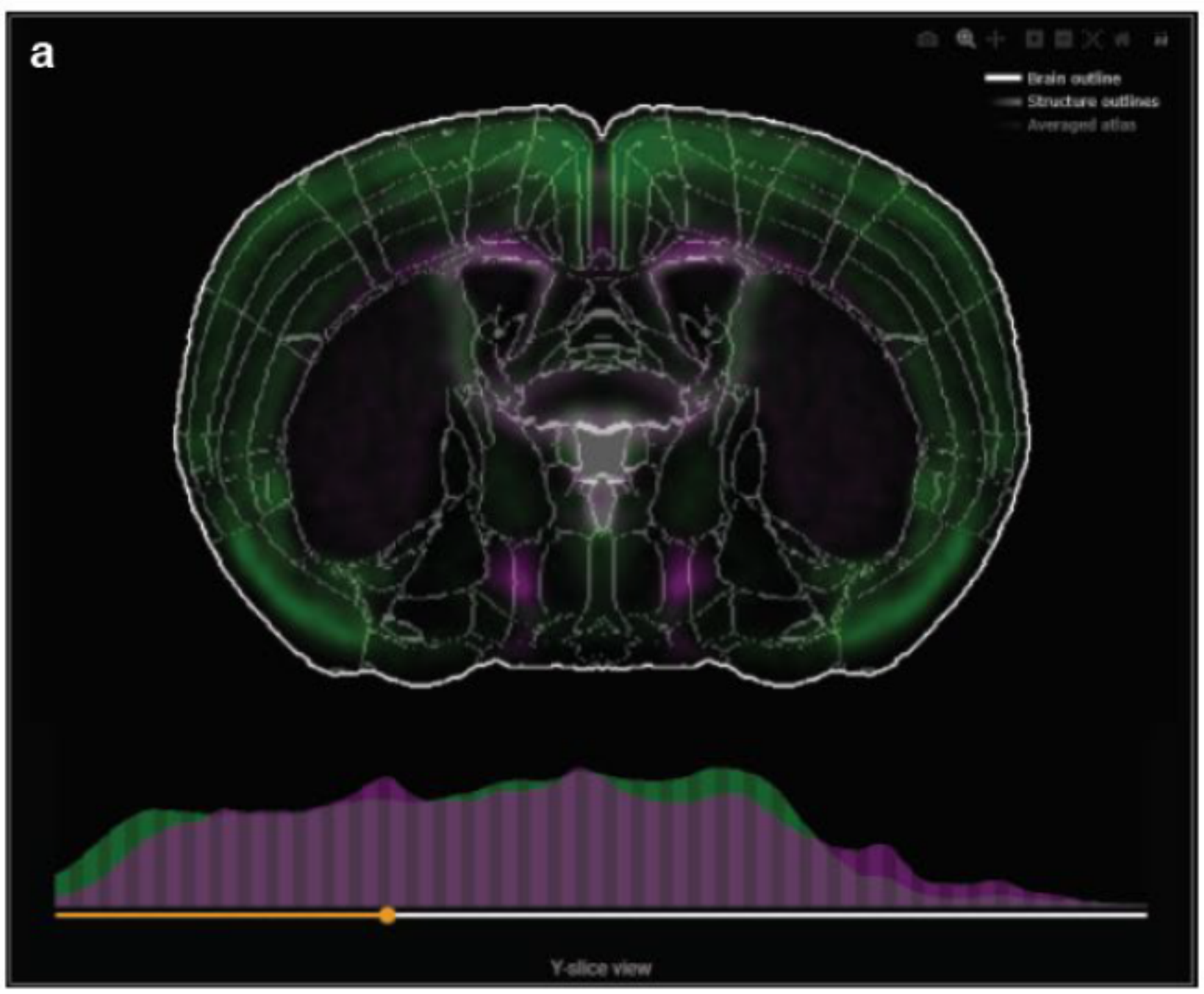

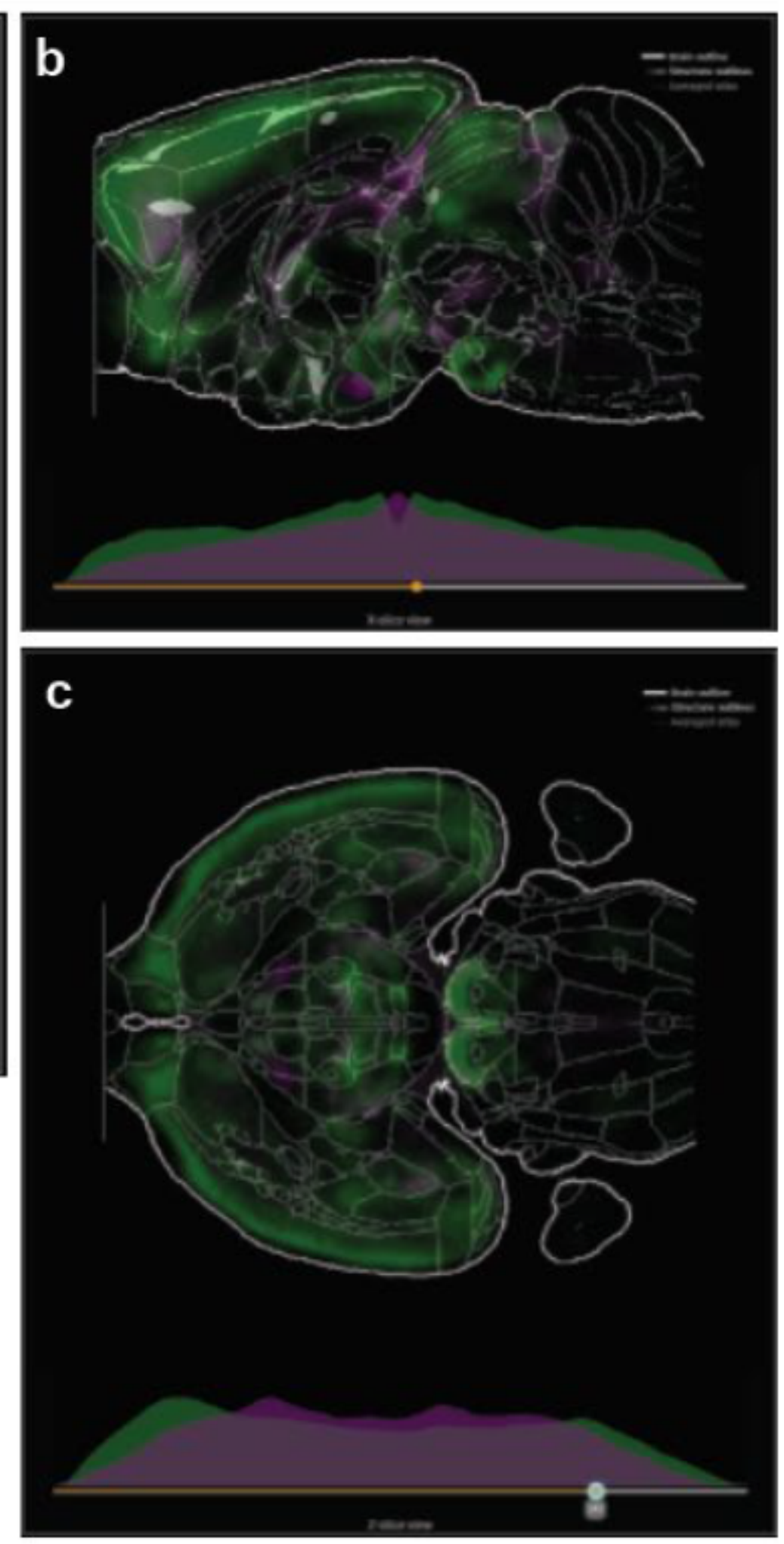

Figure R2: An online resource to explore whole-brain sleep and circadian activity signatures.
Top: Landing page. **a-c,** Example images of Fos heatmaps during novel objects-based sleep deprivation (green) and recovery (magenta).
d-e, Example user-defined query to search for brain areas exhibiting greater Fos expression during recovery relative to deprivation. **f-I,** Visualization of Fos responses segmented by hierarchical clustering. Highlighted areas in h-i exhibit the time profile shown in g.

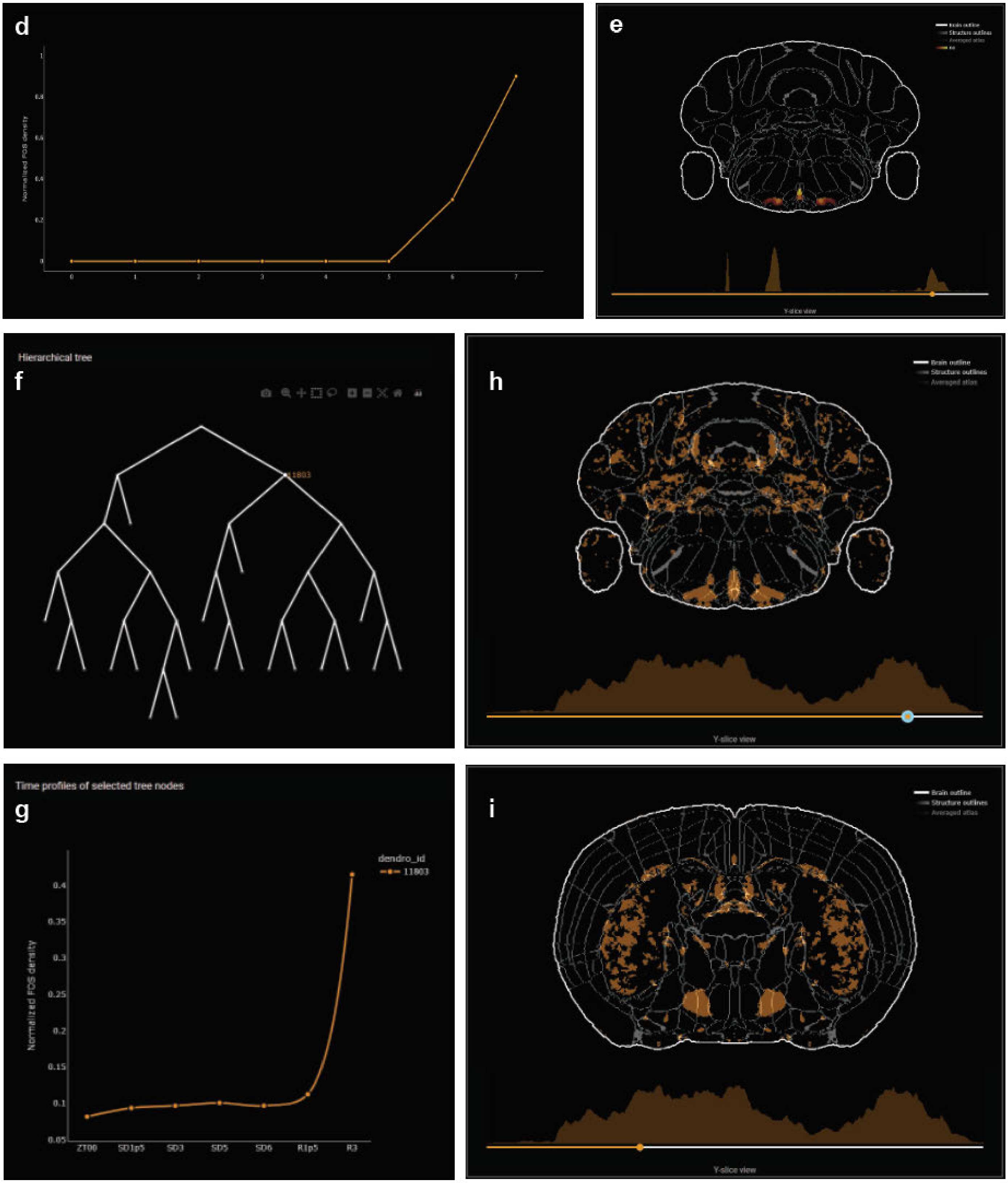
d
Normalized FOS density
e
Y-slice view
Hierarchical tree
f
11803
h
Brain outline
Structure outlines
Averaged atlas
Y-slice view
Time profiles of selected tree nodes
g
dendro_id
11803
Normalized FOS density
ZT00
SD1p5
SD3
SD5
SD6
R1p5
R3
i
Brain outline
Structure outlines
Averaged atlas
Y-slice view

2. Behavioral effects of chronic and profound sleep loss: We reported in the first submission that chronically inhibiting GABA and serotonin cells in the median raphe persistently reduces sleep amount by ~70% (Figure 6, Kir2.1 animals). As pointed out by Referees 2 and 3, this discovery presents a unique opportunity to examine the behavioral and cognitive consequences of chronic sleep loss. We therefore conducted additional experiments and found that Kir2.1 animals are more mobile, but do not exhibit significantly elevated anxiety-like behaviors in the open field or elevated plus maze assays (Figure R3). Kir2.1 animals also spent more time interacting with wood blocks placed in the homecage, as evidenced by the increased surface abrasion and reduced volume of blocks from Kir2.1 animals relative to controls (Figure R4). This suggests that Kir2.1 animals can maintain high behavioral engagement despite their chronic sleep loss, a hypothesis additionally supported by their increased mobility in open field and plus maze assays, as well as their sustained interaction with novel objects during sleep deprivation (Figure 6).

Because sleep deprivation can disrupt learning and memory processes, we also trained Kir2.1 animals in a contextual fear conditioning task, in which mice learn to associate a specific environment with an aversive experience (foot shocks). Memory strength and long-term persistence were assessed by reintroducing mice to the same conditioning environment at recent (1 day) or remote (14 days) timepoints, and measuring freezing behavior and locomotion in the absence of shocks. Interestingly, all Kir2.1 animals were able to encode long-lasting memories, and exhibited freezing levels significantly higher than naïve (pre-shock) baseline up to 14 days after conditioning (Figure R5f). Memory strength was modestly, albeit significantly, weaker than in controls at recent recall (1 day), likely due to elevated mobility when Kir2.1 mice were first reintroduced into the training context (Figure R5g-i). Despite this initial decrease, memory strength reached comparable levels to those of control mice by remote recall (14 days). Thus, chronic ~70% sleep loss does not dramatically compromise the encoding or long-term storage of an associative memory (See Figure R5 or new Extended Data Figure 17, and further discussion in point-by-point responses below). These observations further distinguish MR inhibition from other manipulations that strongly reduce sleep. For example, virally mediated Calcineurin Aα knockout reduces sleep by ~50%, but knockout animals exhibit pronounced anxiety-like behavior and completely abolished contextual and cued fear memory recall (Yin et al 2025).

Together with the absence of rebound sleep/delta (Fig 6), our behavioral analyses support the hypothesis that MR inhibition reduces the detection or buildup of sleep drive and allows animals to maintain high arousal wakefulness for extended time periods.

These new results are now summarized in Extended Data Figures 15, 16, and 17.

Figure R3 (Extended Data Figure 15): MR$^{GABA+Sert}$ Kir2.1 animals do not exhibit significantly elevated anxiety-like behaviors. Cre-dependent Control EGFP or Kir2.1 virus was injected into the MR in *Vgat-Cre; Sert-Cre* animals. **a**, Locomotor traces for three representative control (top) or Kir2.1 (bottom) animals in the open field test. Dotted line indicates center of the arena. **b**, Total distance traveled during the ten minutes of the assay. Gray, Control. Red, Kir2.1. Each dot represents one mouse. **p<0.01, Unpaired t-test. **c**, Cumulative distance traveled. **d**, Percent time spent in the center zone. **e**, Locomotor traces for three representative control (top) or Kir2.1 (bottom) animals in the elevated plus maze assay. Shaded areas indicate enclosed arms. **f**, Total distance traveled during the five minutes of the assay. **p<0.01, Unpaired t-test. **g**, Cumulative distance traveled. **h**, Percent time spent on the open arms.

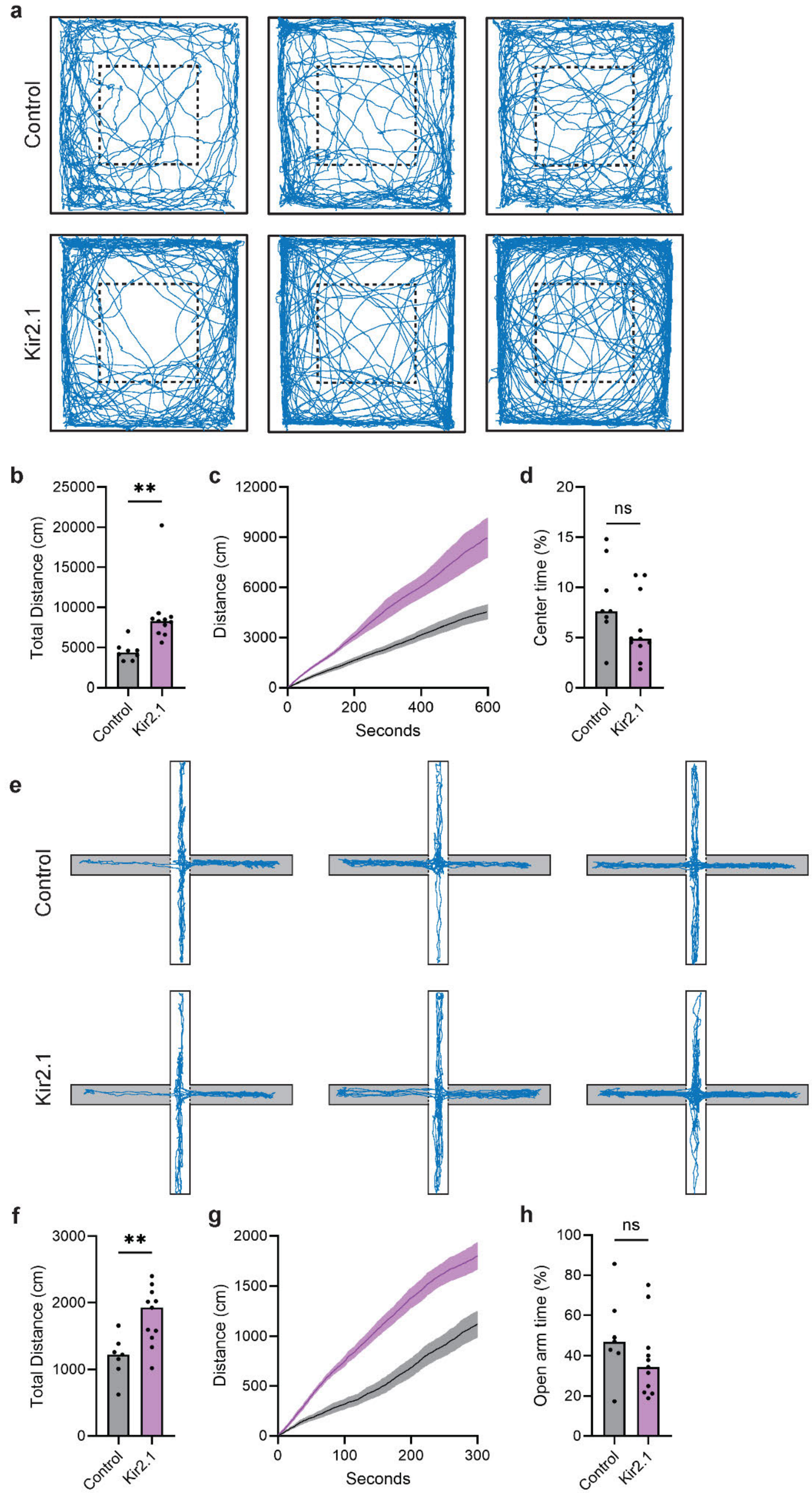

97

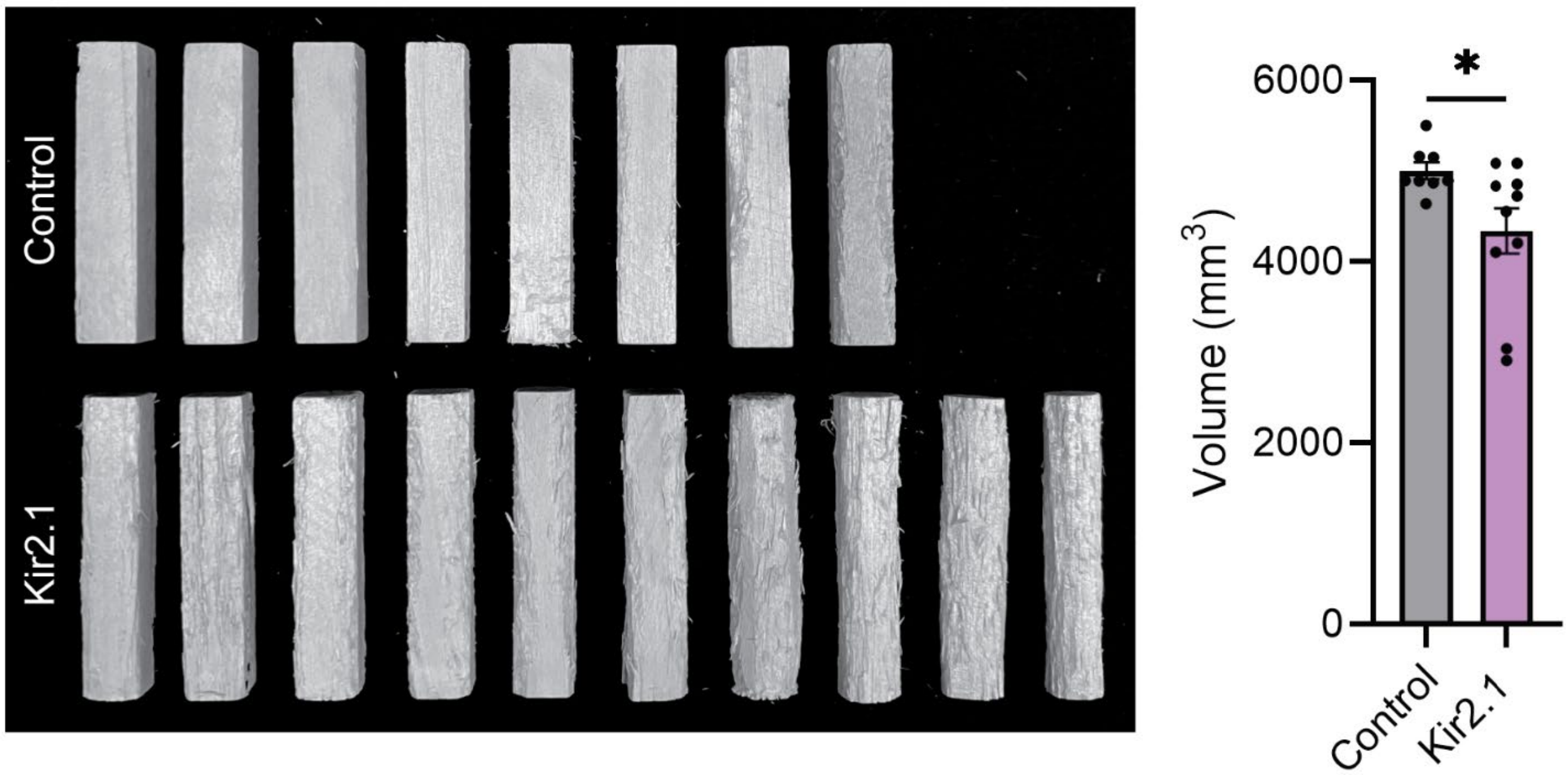

Figure R4 (Extended Data Figure 16): MR$^{GABA+Sert}$ Kir2.1 animals exhibit greater interaction with wood blocks. Left panel: Grayscale image of wood blocks exposed to individual control or Kir2.1 animals, for a period of 3 weeks in the home cage, arranged in order of surface abrasion. Wood block could not be recovered from one Kir2.1 animal, and is presumed completely disintegrated. Right panel: Wood blocks from each animal were 3D-scanned using a tripod-mounted Shining Einscan Pro HD and turntable, and reconstructions were quantified for total volume. Control n=8 blocks, Kir2.1 n=10 blocks. *p<0.05, Unpaired t-test.

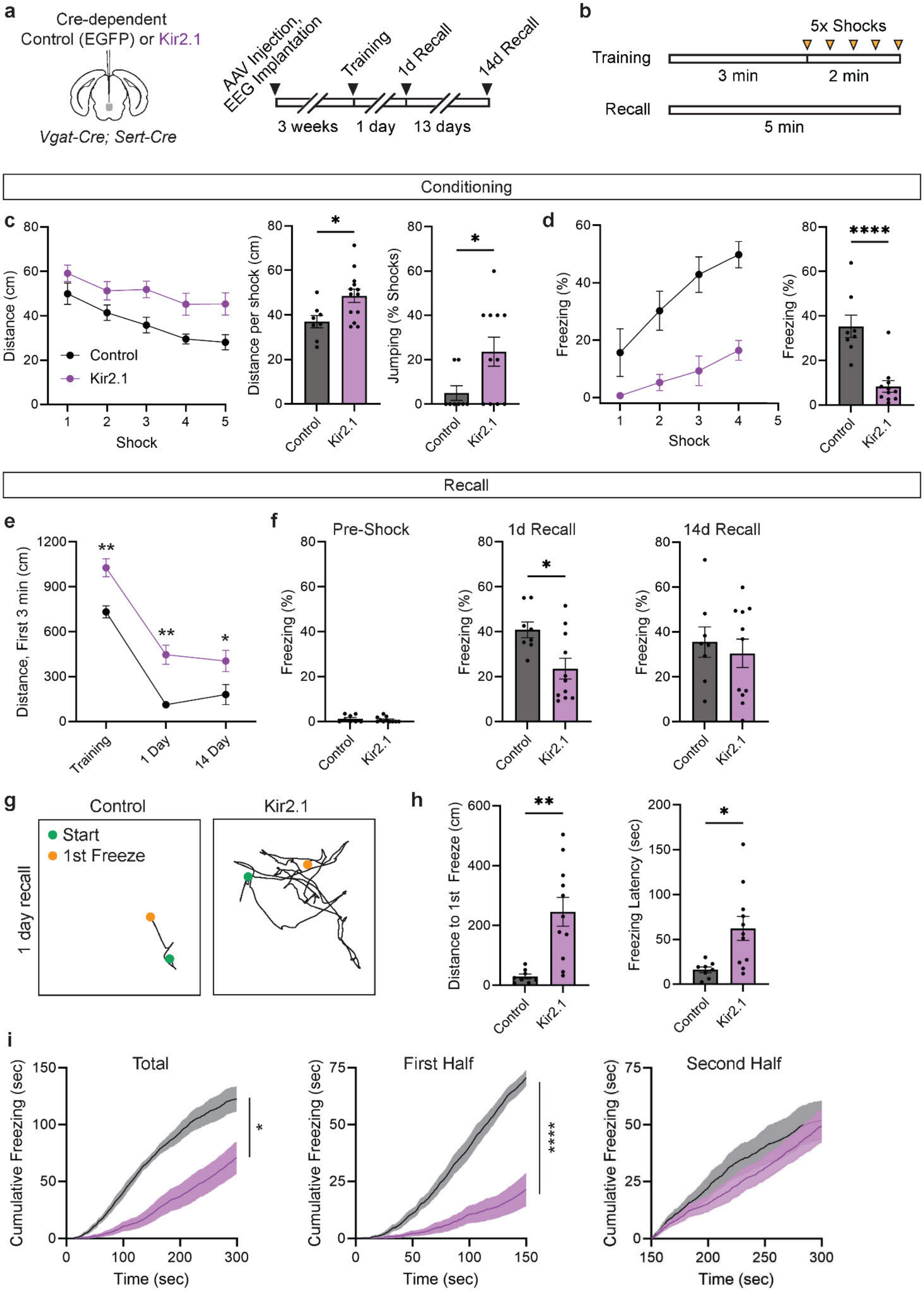
a
Cre-dependent
Control (EGFP) or Kir2.1
Vgat-Cre; Sert-Cre
AAV Injection, EEG Implantation
Training
1d Recall
14d Recall
3 weeks
1 day
13 days
b
5x Shocks
Training
3 min
2 min
Recall
5 min
Conditioning
c
Distance (cm)
Shock
Control
Kir2.1
Distance per shock (cm)
Jumping (% Shocks)
d
Freezing (%)
Shock
Recall
e
Distance, First 3 min (cm)
Training
1 Day
14 Day
f
Pre-Shock
1d Recall
14d Recall
Freezing (%)
g
Control
Kir2.1
Start
1st Freeze
1 day recall
h
Distance to 1st Freeze (cm)
Freezing Latency (sec)
i
Total
First Half
Second Half
Cumulative Freezing (sec)
Time (sec)

Figure R5 (Extended Data Figure 17): $MR^{GABA+Sert}$ Kir2.1 animals form contextual fear memories
a, Strategy to test contextual fear memory. Kir2.1 or EGFP was virally expressed in MR GABAergic and serotonergic cells. Three weeks after virus injection, animals were trained in a contextual fear conditioning task, in which they learn to associate a specific environment (the Conditioned Stimulus, CS) with an aversive experience, a series of 5 foot shocks (Unconditioned Stimulus, US). The encoded memory was tested 1 and 14 days after training, by reexposing animals to the conditioned context in the absence of shocks (CS without US). Freezing behavior was used a readout for memory strength and persistence.
b, Schematic of the contextual fear conditioning task. During training, animals were allowed to explore a specific environment for 3 minutes, before 5 electric shocks were delivered at 30 second intervals. During recall, animals were re-introduced to the same environment for 5 minutes without any shocks. **c**, Left, Horizontal distance traveled in response to each of the five training shocks, measured in the 2 seconds following each shock. Middle and right, Kir2.1 animals travel greater horizontal distances and exhibit more frequent jumping behavior in response to shocks. *p<0.05, Unpaired t-test. Black, Control. Magenta, Kir2.1 throughout figure. **d**, Kir2.1 animals freeze less than controls in between shocks. ****p<0.0001. Unpaired t-test. **e**, Distance traveled during the first three minutes of the training or recall sessions. *p<0.05, **p<0.01, Two-way ANOVA. **f**, Percent time spent freezing during the 3-minute pre-shock period during training (left), or during the 5-minute recall sessions 1 day or 14 days after training (middle and right panels). *p<0.05, Unpaired t-test. **g**, Locomotion trace between start point and first freeze bout during 1 day recall. **h**, Kir2.1 animals travel greater distances and exhibit longer latencies before the first freeze bout. **i**, Cumulative freezing time during the 5-minute recall period. Kir2.1 animals freeze less during the first half of the recall session, but are indistinguishable from controls during the second half. *p<0.05, ****p<0.0001, Unpaired t-test.

3. Electrophysiology of sleep deprivation-responsive MR neurons: In our first submission, Fos mapping and TRAPing approaches indicated increased activation of MR GABAergic and serotonergic cells during sleep deprivation. To examine how sleep deprivation leads to the activation of these cell types, we compared their intrinsic properties in rested or sleep deprived mice using whole-cell patch clamp recordings. Strikingly, sleep deprivation depolarized the membrane potential of GABAergic cells and reduced their firing thresholds, whereas membrane time constants remained unaltered (Figure R6a; also see revised Figure 5 and Extended Data Figure 13). Consistent with these changes, sleep deprived animals exhibited a greater proportion of GABAergic cells that spontaneously fired action potentials, and action potential firing rates were elevated compared to rested controls (Figure R6a and Figure R7g). Furthermore, rheobase was also lower in sleep deprived animals (Figure R7f). In contrast, serotonergic neurons showed no significant changes in resting membrane potential or firing threshold (Figure 5q). Sleep deprivation thus shifts neuronal intrinsic properties towards a more excitable state specifically in MR GABAergic cells. Separate mechanisms likely promote activation of serotonergic neurons, and remain to be identified in future studies.

Our new results indicate that sleep deprivation triggers cell type-specific plasticity mechanisms in the MR. Increased excitability may reflect either cell-autonomous changes or increased presynaptic input. Conceptually related mechanisms regulate Drosophila sleep as well as feeding, mating, and aggression across multiple species (Liu et al 2016; Lee et al 2025; Liu et al 2012; Grzelka et al 2023; Yang et al 2011; Wu et al 2024; Zhang et al 2024; Thornquist et al 2021; Yan et al 2024). Long-lasting plastic changes that persist across hours or days may thus be a common theme between sleep and other motivated behaviors.

These new results are summarized in Figure 5o-q and Extended Data Figure 13.

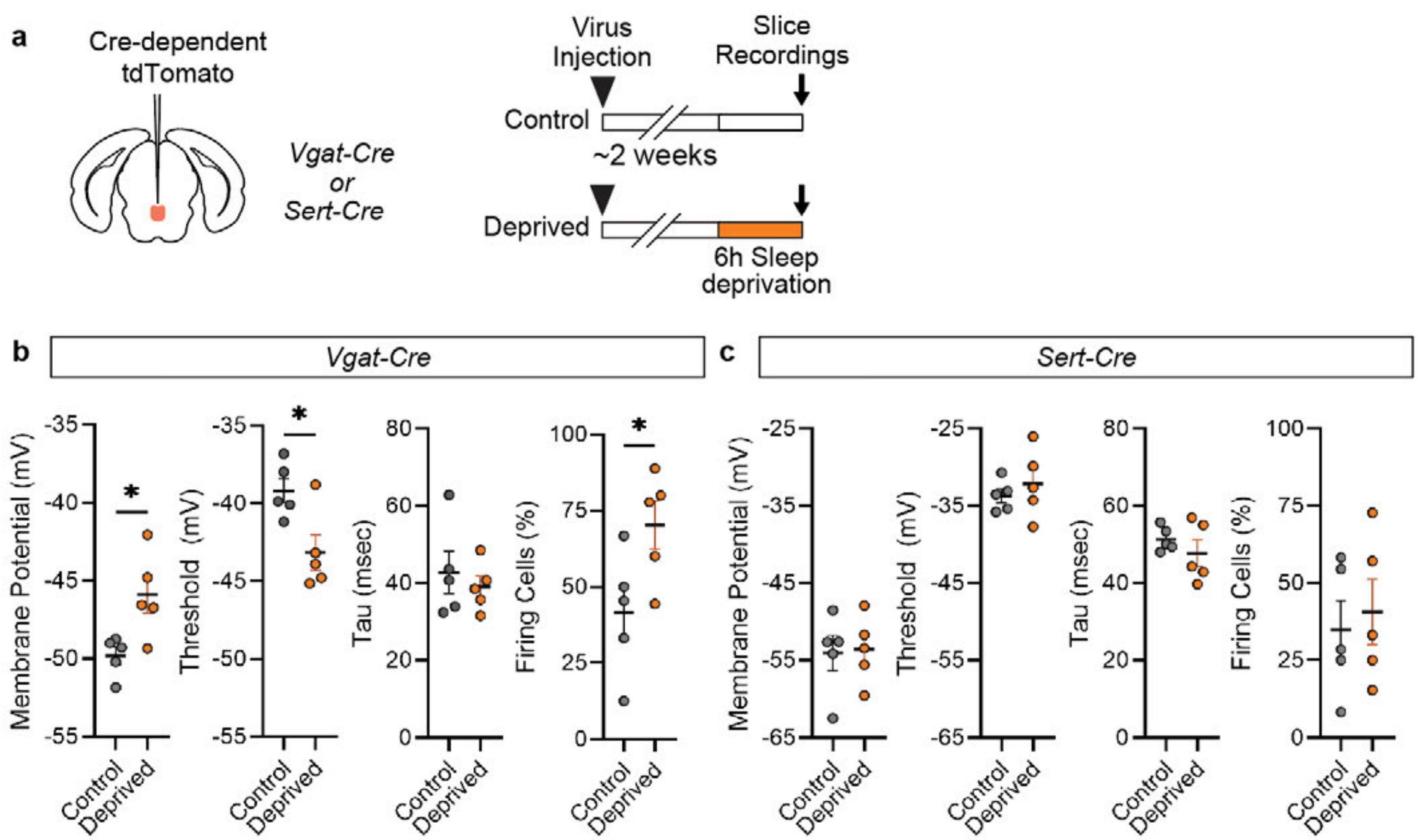

Figure R6 (Figure 5o-q): Sleep deprivation increases MR GABAergic neuron excitability. a, Strategy to examine neuronal intrinsic properties in rested vs sleep deprived animals. Vgat+ or Sert+ cells were virally labeled with Cre-dependent tdTomato, and acute slices were prepared from rested or 6h sleep deprived animals for whole-cell recordings. **b**, Sleep deprivation depolarizes resting membrane potential and lowers firing threshold in Vgat+ cells. Right-most panel, Percent Vgat+ cells per animal that spontaneously fire action potentials. Plots display animal averages. Control n=5 animals, 48 cells. Deprived n=5 animals, 46 cells. *p<0.05, Unpaired t-test. **c**, Deprivation does not alter resting membrane potential or firing threshold in serotonergic cells. Control n= 5 animals, 60 cells. Deprived n=5 animals, 48 cells.

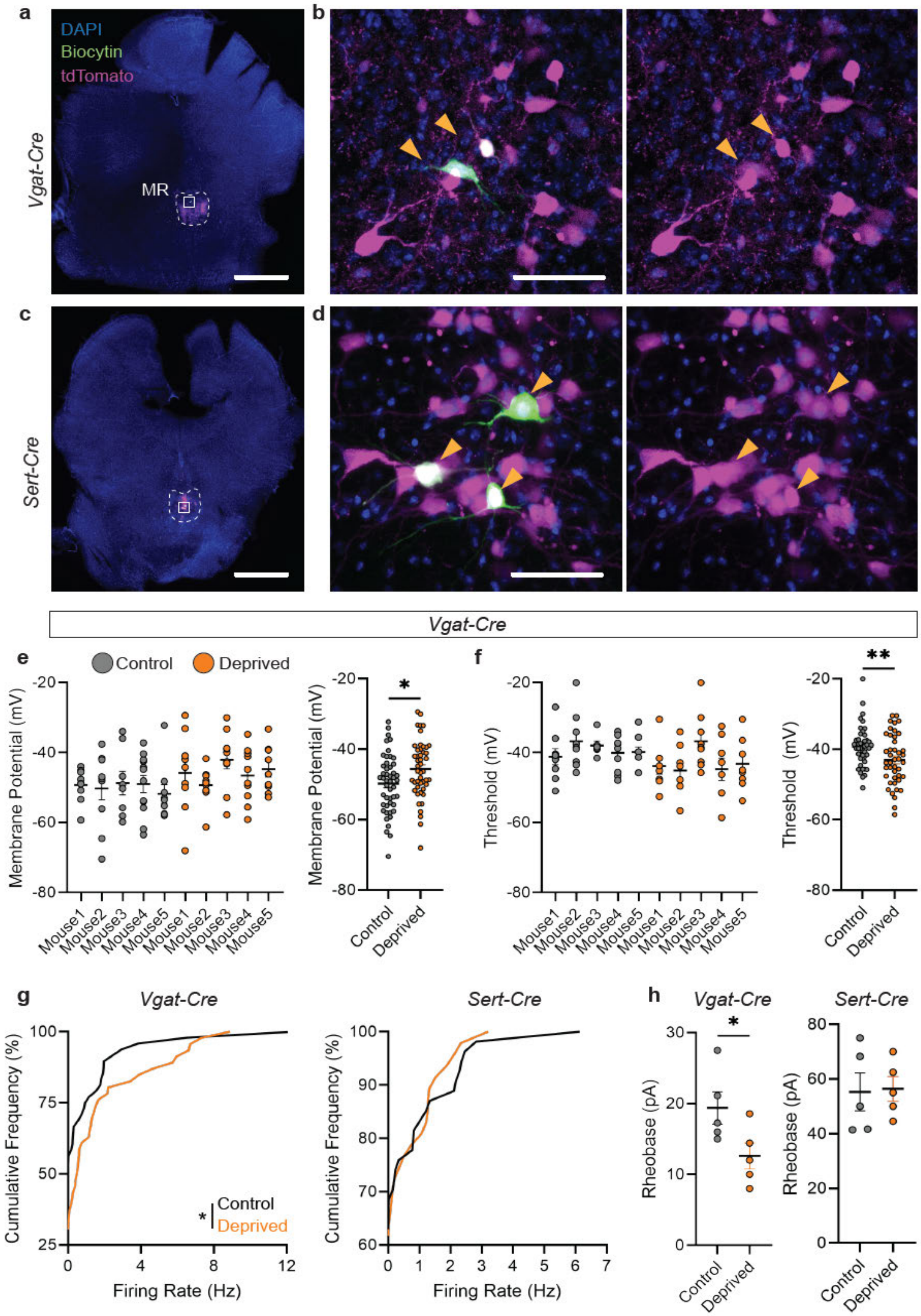

Figure R7 (Extended Data Figure 13): MR slice physiology

a, Representative Vgat-Cre brain section. Dotted lines indicate the median raphe (MR). Cells were targeted for recordings using Cre-dependent tdTomato expression (magenta), and recorded cells were filled with biocytin (green) for post-hoc confirmation. Scale bar = 1 mm. **b**, Higher magnification images of the boxed region in a. Arrowheads indicate recorded tdTomato and biocytin double-positive cells. Scale bar = 50 um. **c-d**, Representative brain section and recorded Sert+ cells. **e-f**, Membrane potential and firing threshold for all Vgat+ cells, segregated by each animal. Right panels, Membrane potential and action potential firing threshold for all cells. Control n=5 animals, 48 cells (Gray). Deprived n=5 animals, 46 cells (Orange). *p<0.05, **p<0.01, Unpaired t-test. **g**, Cumulative distribution of spontaneous action potential firing rates for Vgat+ and Sert+ cells. Sleep deprivation increases Vgat+ cell firing rates. *p<0.05, Mann Whitney U test. **h**, Sleep deprivation lowers rheobase in Vgat+ cells. *p<0.05, Unpaired t-test.

Point-by-point Responses

Below, we provide a point-by-point response (black text) to each referee comment (blue text).

REFEREE 1 (Remarks to the Author):

Sleep homeostasis (catching up on lost sleep) is not a fully understood process. So, identifying how this works precisely could give insights into the function of sleep. Here, Joo et al investigate the circuits, rather than the molecules, that could regulate sleep homeostasis in mice. They find several neuronal types that regulate this, in addition to the ones already in the literature. The manuscript is well written and beautifully presented; I think it is a lovely paper; however, I do not see what is fundamentally new or step-changing about this work (that means it should be published in Nature). The approaches used are fairly standard (cFOS trapping, cell reactivation; potassium channel inhibition, EEG analysis). The final result in the paper is the most impressive one, that co-inhibition of GABA and serotonergic cells substantially reduces sleep amount (I really like Figure 6b!) without influencing sleep homeostasis. As a phenomenon this is intriguing, but then it is not any different from modafinil or some other drugs that promote extended wakefulness without influencing sleep homeostasis.

We thank the referee for their positive feedback. The goal of our study was to identify the circuits that control sleep drive, not drug-induced wakefulness, but we appreciate the referee's thoughtful comparisons to other manipulations. Modafinil is a notable example of pharmacological approaches to extend wakefulness. However, our MR inhibition phenotypes are distinct from the effects of modafinil for two primary reasons:

First, modafinil's mechanism of action is not well-understood, and it remains unclear whether modafinil can regulate or alter sleep drive. Indeed, numerous studies show that, like amphetamines and other stimulants, modafinil increases wakefulness but is followed by increased sleep duration and delta power relative to controls (Edgar & Seidel 1997; Buguet et al 1995; Kopp et al 2002; Qu et al 2008; Bodenmann & Landolt 2010; Vas et al 2020). These studies indicate that modafinil-induced wakefulness increases sleep drive. In contrast, MR inhibition profoundly reduces baseline sleep and abolishes rebound sleep, and can thus drastically reduce sleep drive.

Second, modafinil acts on a short, hours-long timescale, and requires repeated administration for sustained increases in wakefulness. The behavioral effects of long-term modafinil treatment remain poorly studied, although chronic treatment can lead to suppression of theta and gamma power during wakefulness (Vas et al 2020). In contrast, MR inhibition stably reduces sleep by ~70%, and animals can maintain long bouts of high-theta wakefulness.

We have also performed additional behavioral experiments which further distinguish MR manipulations from modafinil and other manipulations (see Figures R3, R4, and R5). In short, MR inhibition allows maintenance of extended wakefulness without the severe memory deficits of high-dose modafinil or amphetamine. Higher doses of modafinil administered during contextual fear conditioning nearly abolished fear memory recall (Shuman & Anagnostaras 2009), whereas all Kir2.1 animals were able to encode long-lasting fear memories (Figure R5). In summary, our experiments thus reveal a novel way to manipulate sleep drive, and (in the words of referee 2) "identify a new target for sleep induction of a different kind than previously identified systems."

I think a key thing missing from the manuscript would be a mechanism for how these "sleep homeostasis" cells get activated; indeed, the authors themselves write at the end of their paper that their results provide "entry points to dissect the molecular mechanisms that ultimately make sleep inescapable". This is very well put, and I feel this is what is needed now (from all of us) to move the field forward, rather than further circuit descriptions.

We agree that the molecular mechanisms underlying sleep remain poorly understood, even in organisms such as *Drosophila*, where dedicated cells and circuits for sleep homeostasis have been more specifically defined than in mammals (Donlea et al 2014 and 2018; Kempf et al 2019; Rorsman et al 2025; Sarnataro et al 2025).

To determine how MR sleep-promoting cells become activated, we compared their intrinsic properties in rested or sleep deprived mice using whole-cell patch clamp recordings. Strikingly, sleep deprivation depolarized the membrane potential of GABAergic cells and lowered their firing thresholds (Figure R6a; also see revised Figure 5 and Extended Data Figure 13). Consistent with these changes, sleep deprived animals exhibited a greater proportion of GABAergic cells that spontaneously fired action potentials, and action potential firing rates were elevated compared to rested controls (Figure R6a and Figure R7g). Furthermore, rheobase was also lower in sleep deprived animals (Figure R7f). In contrast, serotonergic neurons showed no significant changes in resting membrane potential or firing threshold (Figure 5q). Sleep deprivation thus shifts neuronal intrinsic properties towards a more excitable state specifically in MR GABAergic cells. Separate mechanisms likely promote activation of serotonergic neurons, and remain to be identified in future studies.

These results link sleep homeostasis to specific neuronal intrinsic properties in GABAergic neurons, and constitute an important step in further identifying synaptic and molecular mechanisms that regulate the balance between sleep and wakefulness.

In the text, the authors write that "induction of the immediate early gene Fos can label activated neurons that control homeostatic behaviours such as feeding or drinking" (line 46). At the same time, the authors give the, I am sure unintentional, impression that this method has not been used to study sleep homeostasis. Yet cFos-trapping and cell reactivation was first used to identify preoptic hypothalamic neurons involved in mouse sleep homeostasis (and temperature regulation) ten years ago (ref. 30, Zhang et al 2015). Since then, a whole range of neuronal types have been identified, not all mentioned by Joo et al., as participating in mouse sleep homeostasis (for example, preoptic galanin neurons (ref. 32 Ma et al, but also galanin neurons in zebra fish, Reichert et al 2019, Neuron), VTA GABA neurons (Yu X et al 2021,Mol Psychiatry, PMID: 32555422), prefrontal cortex somatostatin neurons identified by cFos trapping, Tossell K et al 2024 Nat Neurosci) that contribute to catching up on lost sleep; in fact, I think it is quite likely that the homeostasis network will be distributed, and that these "homeostasis cells" will just be the same cells as those that induce sleep without sleep deprivation.

We thank the referee for drawing connections to these previous studies, and have now included the previously uncited references and others in the main text. We have also revised the original sentence to clarify that Fos has also been used to identify sleep-promoting cells. Our study extends these and other studies by comprehensively profiling Fos dynamics during the course of deprivation and during natural variations in sleep pressure in undisturbed animals. In the words of referee 2, this approach is "impressive since it is done for the first time with so many time points in control conditions, during sleep deprivation, and during recovery." We have also created an online resource that allows users to browse and query the whole-brain datasets. Our observations indeed support the idea that "the homeostasis network will be distributed" (Fig 1), and future studies must further dissect how such a network acts to regulate sleep. Based on the referee's comments, we have also modified the discussion to include the idea that the MR acts as part of a distributed network that regulates sleep drive.

"Specific points, Major:

1. Although using immediate early genes like c-fos is a useful and accessible approach for observing and quantifying neuronal activation, it lacks high temporal resolution due to the time required for c-Fos protein synthesis following c-fos gene expression. Since immunostaining was used to assess activity changes, the measured density at each time point does not represent real-time activity but rather reflects expression levels approximately 1–1.5 hours prior. For example, the c-Fos density at 1.5h into the recovery sleep in Figure 1 indicates the neuronal activity at the end of the 6h sleep deprivation. This would suggest that the activity of Type 3 neurons has already started to decline before the recovery sleep was initiated. This pattern is similar to that of Type 1 group but only with greater intensity."

Fos protein expression indeed lacks the temporal resolution of other neuronal activity measures such as calcium indicators. However, previous studies show that Fos protein can decay rapidly after the cessation of activating stimuli. For example, in brain regions that regulate nutrient or fluid homeostasis, Fos protein expression decays within 0.5-2h after post-deprivation refeeding or rehydration (Becksei et al

2009; Tan et al 2014; Ji et al 2005; Wotus et al 2007; Basheer et al 1997). Similarly, Fos protein can return to baseline levels 1h after the end of sleep deprivation in specific cortical areas. Likewise, Fos protein expression in V1 declines by ~50% within 30 minutes after the end of visual stimulation, and can return to baseline 1h later (in alternate visual stimulation protocols, V1 Fos protein decays significantly by 2h post-stimulation; Zangenehpour & Chaudhuri 2002; Yang et al 2024). Based on these reports and others, we believe that decreased Fos 1.5h into recovery sleep is consistent with decreased neuronal activation during the 1.5h recovery period, and does not necessarily indicate a preceding decline during sleep deprivation ("before recovery sleep was initiated").

Nonetheless, we agree that future studies must determine how Type 3 Fos patterns relate to bona fide neuronal activity and sleep/wake behavior. In response to the referee's comment, we conducted new electrophysiology experiments, and found that sleep deprivation increases the excitability of MR GABAergic cells (see Figure R6 and R7, revised Figure 5 and Extended Data Figure 10). These results are consistent with increased Fos activation, and indicate that sleep deprivation triggers plastic changes in the MR.

"2. The authors should show in vivo whether Type 3 (MR and aMPO) neurons reflect the duration of wakefulness to support their hypothesis. Does the activity of these neurons increase as sleep loss accumulates? Are they more active at the end of the dark period compared to that at the beginning of the light period?"

To examine how sleep deprivation leads to activation of MR GABAergic and serotonergic cells, we compared the intrinsic properties of both cell types in rested and sleep deprived mice using whole-cell patch clamp recordings (Figures R6-7 above. Also see revised Figure 5 and Extended Data Figure 13). Strikingly, sleep deprivation depolarized the membrane potential of GABAergic cells and lowered their firing thresholds, whereas membrane time constants remained unaltered. Accordingly, sleep deprived animals exhibited a greater proportion of GABAergic cells that spontaneously fired action potentials, and action potential firing rates were increased relative to rested controls (Figure R6a and Figure R7g). Consistent with these effects, rheobase was also lower in sleep deprived animals (Figure R7f). In contrast, serotonergic neurons did not exhibit significantly altered resting membrane potential or firing threshold (Figure 5q). Sleep deprivation thus shifts neuronal intrinsic properties towards a more excitable state specifically in MR GABAergic cells. Separate mechanisms likely promote activation of serotonergic neurons, and remain to be identified in future studies.

These new results are consistent with increased Fos activation in the MR, and suggest that sleep deprivation triggers plastic changes, either cell autonomously in GABAergic cells or in their presynaptic inputs. In future studies, we hope to extend these observations to additional brain areas and timepoints using both electrophysiology and in vivo imaging-based approaches.

"3. The statement in Line 73 - "…Type 3 regions do not selectively respond to stress or aversive stimuli." is not supported by experimental data. The process of sleep deprivation itself is aversive and the fact that the activity of these neurons is higher during sleep deprivation than that in the baseline undisturbed wakefulness suggests the opposite. Type 3 neurons could be responding and essential for maintaining high level of arousal caused by external aversive stimuli."

We believe that at least some Type 3 regions may reflect wake history rather than stress, as they increase during the night and decrease during the day, in the absence of any manipulations or potentially stressful deprivation stimuli. Nonetheless, we agree that Type 3 cells may also include neurons that respond to or maintain high arousal, hence the necessity for functional tests as in Figure 2 or Extended Data Figure 6. We have modified the original statement as follows: "The consistent wake-correlated responses during both sleep deprivation and undisturbed sleep/wake behavior suggest that Type 3 regions are not selective for sleep deprivation, but may also reflect natural changes in sleep drive in the absence of experimental stimuli."

"4. It is unclear when wake-active MR/aMPO neurons promote sleep when activated. Do they detect sleep loss and subsequently trigger sleep at a threshold? In the undisturbed condition, MR neurons become less

active during sleep, suggesting their activity is not required for normal sleep. Why do they induce sleep upon activation? (refer to Figure 2). Also during sleep deprivation, these neurons show high activities but do not immediately induce sleep. Similarly, artificially activating them do not necessarily induce sleep (refer to Figure 2). How to interpret this discrepancy?"

MR and aMPO neurons indeed promote sleep. We show in Figures 2, 4, and 5 that activating these cells using TRAP, projection-restricted, or cell type-specific approaches all robustly induce sleep. During experimental sleep deprivation, we hypothesize that separate wake-promoting circuits oppose the otherwise sleep-promoting effects of MR/aMPO cells. For example, the LHA is also activated during sleep deprivation, and LHA Deprivation-TRAP cells promote wakefulness, likely through orexinergic projections (Figure 1 and Extended Data Figure 6). Accordingly, while MR/aMPO cells promote sleep, animals do not stably transition into sleep until sleep deprivation stimuli cease.

The threshold mechanism mentioned by the referee is one exciting possibility for how the MR promotes sleep. The MR activates the preoptic area while suppressing the LHA (Figure 4), making it well-poised to bias multiple circuits towards sleep when sufficiently activated. However, it remains unclear how the opposing influences of sleep and wake centers interact during ongoing behavior. This is a fascinating question for the sleep field, which we believe is beyond the scope of the present study.

"5. In Figure 3i, sleep duration does not seem to change in light phase. If the sleep deprivation was performed using the same protocol at the beginning of the light phase, what cause the changes in Figure 3m and n? Does the delta power change in during the light phase when inhibiting aMPO?"

We find that delta power increases at a lower rate in aMPO Kir2.1 animals relative to controls (Figure 3l), suggesting slower sleep pressure accumulation. This likely contributes to the delayed increase in sleep attempts during sleep deprivation. Nonetheless, as the referee points out, the effects of Kir2.1 expression in aMPO are weaker than in MR. We propose that Kir2.1-expressing aMPO cells still become activated enough to promote normal baseline sleep, but further activation during deprivation is blunted, thus decreasing sleep attempts (Figure 3m).

"6. In Figure 3 and 4, the authors specifically targeted MR or aMPO neurons that are selectively active during sleep deprivation, which typically represent a small subset of neurons. In contrast, in Figure 5 and 6, the authors manipulated entire populations of GABAergic, serotonergic and glutamatergic neurons, including many that do not respond to sleep deprivation. This suggests that the sleep-promoting effect is a general property of certain MR populations without interfering with sleep homeostasis. Notably, this is similar to previously identified serotonergic neurons in the dorsal raphe and GABAergic neurons in the VTA, which are wake-active but can generate NREM sleep upon activation."

Deprivation-TRAP indeed labels fewer cells than Cre lines. We believe Deprivation-TRAP underestimates the fraction of deprivation-responsive cells for the following reasons: 1) TRAP typically labels a subset of Fos+ cells for a given stimulus. For example, Fos immunostaining labeled at least ~3-fold more cells than TRAP in auditory cortex. Similarly, Thirst-TRAP labeled ~60% of MnPO Fos cells induced by dehydration, while Anesthesia-TRAP labeled ~25% of Fos cells across different brain regions (Tasaka et al 2018; Allen et al 2017; Yatziv et al 2021). 2) Fos expression itself underestimates the fraction of neurons with changes in activity. Indeed, Fos expression does not always correlate with increased neuronal spiking, and its response to other types of activity patterns is not well-understood across different cell types. In short, the fraction of MR neurons that respond to sleep deprivation likely exceeds the ~40% of serotonin cells and ~20% of GABAergic cells labeled by Deprivation-TRAP (Figure 5a-c).

Consistent with this idea, we were able to detect sleep deprivation-induced changes in GABAergic cell excitability as well as an increased frequency of spontaneously firing cells and higher firing rates, even under the sparse recording conditions of slice electrophysiology (See Figures R6-7 above or Figure 5o-p and Extended Data Figure 13).

We thank the referee for drawing interesting parallels to other sleep-promoting cells in the DR and VTA. While optogenetically activating DR serotonergic cells can promote sleep, their specific contributions to sleep drive remain unclear, as the Oikonomou et al 2019 study ablates all serotonergic neurons in the DR and MR, with milder effects than our MR inhibition experiments. In contrast, we demonstrate that MR

GABAergic cells can synergistically promote sleep with MR serotonergic cells, and that these specific populations are crucial for normal sleep drive.

MR sleep-promoting cells also exhibit intriguing differences with VTA GABAergic cells. For example, VTA GABAergic cells are activated by aversive experiences like air puffs or social defeat stress (Harris et al 2022; Yu et al 2022), and are thought to promote sleep following stressful experiences to allow animals to reduce anxiety and restore mental and bodily functions. It remains unknown how VTA GABAergic cells respond to sleep deprivation, but an Sst+ subset exhibited minimal responses, and could even be suppressed by deprivation (Yu et al 2022). Another study observed higher population activity of VTA GABAergic cells during NREM sleep rather than wake, and neuronal activity during NREM was required for the maintenance of sleep (Chowdhury et al 2022). In contrast to these studies, we find that the MR is activated during two different sleep deprivation regimes, and also during periods of naturally high sleep drive in unperturbed animals (Figure 1). Furthermore, ablating VTA GABAergic cells primarily reduced sleep during the active dark phase (Yu et al 2018), whereas chronically inhibiting MR GABA/Sert cells severely reduced sleep during both light and dark phases (Figure 6).

In summary, our manipulation experiments indicate that the MR is a crucial regulator of sleep drive, and differentiate MR GABA/serotonin cells from sleep-promoting populations in the DR or VTA. Future efforts must unravel how the distinct circuits emanating from the MR, DR, VTA, and other regions cooperate to regulate sleep and wakefulness.

“Minor:

1. In figure 2 and 5, the effect of CNO appear to be longer than the time points shown on the x-axis. It would be better to present the results over a longer duration e.g. 12h to capture the dynamics of these changes.”

We have updated the plots in Figure 2 and Figure 5 to include the entire time window of phenotypes induced by chemogenetic activation/inhibition.

“2. Figure 2, n=3 for recovery TRAP which is low.”

We have analyzed additional animals for this experiment, bringing the sample size to n=11 for Deprivation TRAP and n=8 for Recovery TRAP. The conclusions remain unchanged.

“3. In Figure 3, it would be better to represent the changes of baseline sleep in light and dark phases separately.”

We have now created a new extended data figure that quantifies changes in sleep/wake separately between light and dark phases.

“4. In figure 4f, no presentative images shown.”

We now include representative images of Cre/Flp intersectional hM3Dq-mCherry expression in the MR in Figure 4g.

REFEREE #2 (Remarks to the Author):

Note: For clarity, we have added numbers to each of the referee's comments.

In their report the authors use multiple methodologies to demonstrate that a category of wake-active neurons regulate sleep drive. Below I comment their findings following the paper organization.

1. "I see some artefactual staining on the tissue surrounding the ventricules in their illustrations. In these regions, Fig 1C, some Type 3 neurons in particular are localized in these areas. I wonder whether the authors used a way to remove artefact counting?"

As summarized in the Methods, we automatically detect Fos-positive cells using a stringent LoG filter, and include all detected cells without further corrections. As the reviewer points out, we observe Fos signal around the ventricle walls, and believe it represents bona fide signal rather than artefacts that must be removed, for the following reasons:

1. We confirmed that Fos-positive cells could be detected in or near ventricular walls using conventional immunostaining in 2D coronal sections, suggesting that ventricular signal is not an artifact of the iDisco+ method.
2. We also confirmed that ventricular signal could be detected using different Fos antibodies, as well as an antibody for phospho-S6, another marker for cellular activation (Knight et al. 2013, *Cell*).
3. As negative controls, we confirmed that ventricular signal is absent from the 561nm autofluorescence channel. Ventricular signal was likewise absent in controls with no primary antibody, and iDisco stains with other antibodies such as anti-RFP.
4. Finally, to visualize Fos upregulation in the ventricle using an antibody-independent method, we imaged 2D sections from *TRAP2; Ai14* (tdTomato Cre reporter) animals and "TRAPed" during sleep deprivation. Indeed, we observed tdTomato+ cells within or close to the ventricle wall, consistent with iDisco Fos signals.

These results are summarized in the figure below (Figure R8), and cumulatively indicate that peri-ventricular Fos signal is not artefactual. We now include these data in a new Extended Data Figure 4.

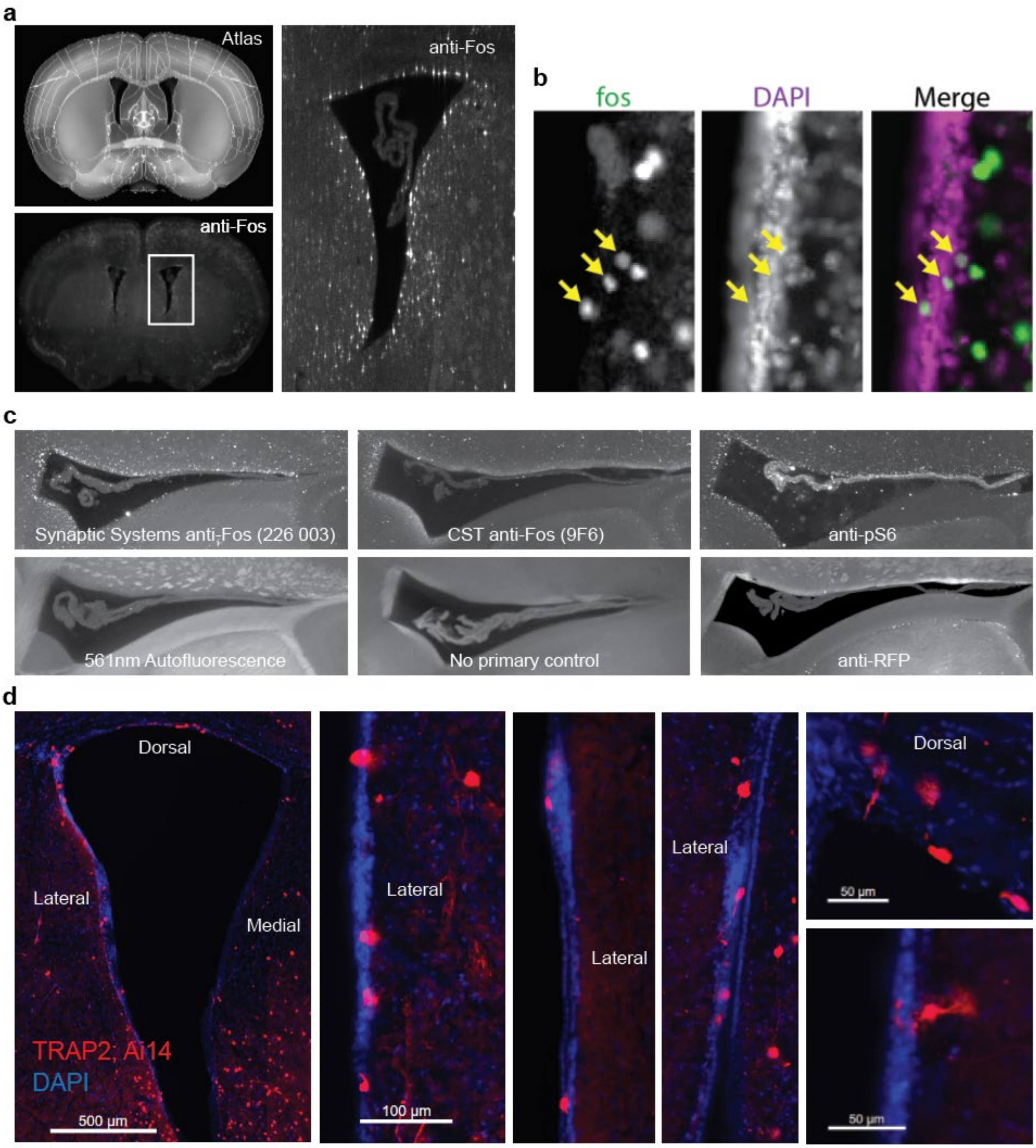

Figure R8 (Extended Data Figure 4): Peri-ventricular Fos-positive cells.
a, Representative coronal views of brain atlas and Fos immunostaining using the iDisco+ and light-sheet imaging. Sample was taken 6h into sleep deprivation. Right panel shows higher magnification of the lateral ventricle (boxed area on left). **b**, Fos-positive cells (yellow arrows) are detected in the lateral ventricle wall after conventional immunostaining of 2D coronal sections. Green, Fos; Magenta, DAPI. **c**, Top row, Representative horizontal views of the lateral ventricle in iDisco samples stained with two different Fos antibodies or phospho-S6 (pS6), another marker of cellular activation. Bottom row, Ventricular signal is absent in the 561nm autofluorescence channel, a control without Fos primary antibody, and immunostaining with RFP antibody. **d**, In *TRAP2; Ai14* animals, tdTomato+ cells (Red) are visible in the dorsal, lateral, and medial ventricular walls after Deprivation-TRAP. Blue, DAPI.

2. “The cFos staining shown in Extended Figure 2 does not look in focus and for some images neurons have strange morphology with vertical lines extending from them? Is it due to the clearing process?”

Brains were imaged using lightsheet microscopy in horizontal orientation, but we display coronal sections in Extended Data Figure 2, as coronal views are standard for neuroanatomy. The “streaking” observed is thus due to imaging vs reslicing orientation, rather than physical artifacts due to clearing. We have updated the corresponding method section to make this more explicit.

3. “For some structures, like the first photo on the left of VTA it is really out of focus and the neurons shown don’t seem to be inside the VTA but more the interpeduncular nucleus (IP)?”

We have added additional anatomical landmarks to these images, which now allow readers to clearly distinguish the boundaries of VTA vs IPN.

4. “For S1 there is a strong labeling in layer 1 particularly after 3h of deprivation. This is quite unusual and the size of the neurons seems big. Did the authors verified that it corresponds to neurons?”

We believe the labeling referred to here corresponds primarily to superficial layer 2. We did not test whether these Fos+ cells correspond to neurons or other cell types. Our observations are consistent with other studies that have observed strong activity-regulated gene expression in superficial layer 2 following sleep deprivation (Thompson et al 2010). We make no claims regarding the activated cell types in S1, and believe a detailed characterization of these cell types is beyond the scope of this paper. In response to the referee’s comments, we have added additional anatomical landmarks to Extended Data Figure 2c to more clearly delineate layer 1 vs layer 2/3.

5. “The authors state that to control for stimulus-specific effects, they compared these regimes to sleep deprivation during the dark phase. What they mean by stimulus-specific effects? Like the method for depriving? However, it might be that the differences are due to the fact that wakefulness occurring during the night is not inducing the same cFos staining that during the day. In addition, circadian time could induce also other neuronal activation.”

We appreciate these insightful comments. We indeed hoped to distinguish deprivation stimulus-induced signals from sleep/wake or circadian-related signals. As stated in the text, Type 1 responses decay early during deprivation, are largely similar between light vs dark deprivation, and respond minimally during unperturbed behavior (Figure 1). In contrast, Type 3 responses exhibit wake-correlated responses during both light/dark deprivation and unperturbed behavior. Together, these data suggest that 1) Type 1 responses “correlate with sensory stimulation, novelty, or cognitive engagement rather than wake duration,” while 2) Type 3 responses reflect wake duration rather than stimulus response. In summary, as the referee suggests, we were able to isolate candidate regions that regulate sleep drive by comparing different sleep deprivation regimes (day vs night, grooming vs novel objects) with unperturbed circadian behavior. In response to the referee’s comments, we have revised the corresponding sentence of the main text as follows: “The consistent wake-correlated responses during both sleep deprivation and undisturbed sleep/wake behavior suggest that Type 3 regions are not selective for experimentally induced sleep deprivation, but may also reflect natural changes in sleep drive in the absence of experimental stimuli.”

6. “The authors state that they used voxel-wise clustering of Fos density across experimental timepoints to reveal three types of subtypes. I assume that it corresponds to three subtypes of structures although it is not clearly mentioned at that point of the text. What is not clear in the text, in the Figures including Fig 1 and extended Figure 2-3 is how they did discriminate these regions? There is indeed no statistical tests shown in the text, the figures or the method section. Because of that lack I’m not sure what means the segregation between the different types. Further, it is well know that for nearly all structures there is an overlap of wake and sleep-active neurons. How they deal with such question?”

We apologize for any ambiguity in the text. Since we use a hierarchical clustering approach to segment the brain into different response profiles, the three primary response types each encompass

distinct voxels. While we do not restrict our analyses using pre-determined brain atlas boundaries, these voxel groupings indeed populate specific sub-regions in the brain atlas, as shown in Figure 1c and Extended Data Figure 3. In particular, Extended Data Figure 3 illustrates example regions that are classified as Type 1-3 based on voxel clustering (i.e. where in the brain are the clusters?). In new supplementary figures and the revised methods section, we now include additional details on our clustering approaches. As requested by the referee, we have also added additional statistical analyses to Fos density quantifications in Figure 1, Extended Data Figure 3, and Extended Data Figure 7.

As the referee reminds us, most brain areas contain heterogeneous cell types with distinct activity patterns or functions. We use Fos to highlight potential brain regions of interest, with net changes in neuronal activation. In other words, we focused on regions where the dominant Fos signal correlated with sleep/wake behavior. We then used the TRAP approach to label potentially intermingled neuronal ensembles and identify the relevant cell types (Figure 2, Extended Data Figure 6, Figure 5). Finally, we tested the functions of individual neuronal types (Figure 5-6). Cumulatively, this experimental approach allowed us to distinguish sleep-promoting vs wake-promoting cells in the MR and aMPO.

7. "I'm also a bit skeptical on the neuroanatomical landmarks used in Figure 1. Indeed there is a mixture of classical structures: DG, dentate gyrus; MR, median raphe; VTA, ventral tegmental area; With some subdivisions of structures never reported before to my knowledge: VMHdm, ventromedial hypothalamus, dorsomedial portion; aMPO, anterior medial preoptic area LHAdl, lateral hypothalamic area dorsolateral portion Why the authors made such subdivisions? For example for the LHAdl, where is it located? It could be the zona incertain in fact? And the LHA is a very extensive structure, is it rostral or caudal LHA? One important denomination is the aMPO. I'm not sure it is not misleading name since from their Extended Figure 10 it rather seems to correspond to the Median preoptic nucleus as stated by the Allen Atlas. Finally they used quite vague denominations for other structures like: pHbMB, peri-habenular midbrain; PTh, posterior thalamus; There are known structures in these regions. Why they dont use classical terminology? The peri-habenular midbrain is an interesting name but I have no idea where it is located."

We apologize to the referee for any lapses in clarity. Extended Data Figure 3 summarizes the anatomical locations of the regions indicated in Figure 1d - this is stated in the text, and we have now also revised the figure legend to further direct readers to the corresponding data.

Type 1-3 responses do not correspond perfectly to Allen Brain Atlas labels. For example, Type 3 responses are enriched in a portion of the midbrain directly behind the habenulae, which we refer to as the peri-habenular midbrain (pHbMB, Extended Data Figure 3a). The Allen Atlas assigns only the general "Midbrain" label to this region, necessitating a more specific (if provisional) name.

Where possible, we also specify subdivisions of known regions. We believe noting these subdivisions is important because some regions are (as the referee points out) very extensive. For example, Type 2 responses are clearly enriched in the dorsomedial portion of the VMH (Extended Data Figure 3a), hence our "VMHdm" nomenclature. Likewise, Type 3 responses are enriched in the anterior portion of the MPO, hence the "aMPO" abbreviation. To further orient readers to the anterior/posterior extent of these subregions, we have added additional anatomical landmarks and injection maps to Extended Data Figures 5, 7, and 18.

Furthermore, most of our anatomical subdivisions have been previously reported. For example, neuroanatomical, functional, and transcriptomic studies clearly distinguish between dorsomedial and ventrolateral VMH (VMHdm and VMHvl, respectively - Lindberg, et al; Wang, et al; Affinati et al; Meek et al 2016; Sabatini et al 2021). Similarly, multiple studies divide the LHA into medial vs lateral areas, or even finer subregions (Yoshida et al 2006; Harris et al 2005; Gonzalez et al 2012; Wang et al 2021). Therefore, we believe specifying anatomical subregions is highly relevant to the field.

For more detailed insight into regions not analyzed in this paper, readers can now browse the whole brain Fos data using our new web resource (See Figure R2 above or https://sleep-wake-atlas.scicore.unibas.ch/). Users can browse data according to experimental timepoints, query by region or activation pattern, or segment the brain by hierarchical clustering. We hope this can serve as a useful resource for the neuroscience community.

8. “In summary, the whole brain analysis made is impressive since it is done for the first time with so many time points both in control conditions, during sleep deprivation and sleep recovery.”

We thank the referee for their enthusiasm.

9. “In the text, the authors used the term voxel but in the Fig1. Legend they wrote cFos density. Is it really a density meaning that they divided the number of cFos+ neurons by the surface of the structures? It seems not since it is based on voxel analysis. May be this is the reason why they found in some cases that only subparts of a structure are activated. I’m not sure this is the way to go since it highly depends on the alignment of each brain with the atlas. It is certainly different between brains and therefore very difficult to be sure all voxels are exactly in the same subpart of a structure. It would make more sense to compare a mean of the density in a whole classical structure defined by the atlas.”

As in previous volumetric or whole-brain quantifications of Fos (Yamaguchi et al 2023; Hansen et al 2021; Renier et al 2016), we voxelize Fos signal after registering to a brain atlas, and compare voxel intensities between groups. As stated in the Methods section, we voxelize Fos signal by applying a Gaussian blur to registered Fos+ nuclei. In this scenario, voxel intensity provides an empirical probability density of Fos signal, which we call Fos density. Areas with denser Fos induction generate voxels with higher intensity values. For each experimental timepoint, we compare voxel intensities generated from 6-9 animals. As the referee assumes, this is indeed why we can resolve Fos induction in specific sub-regions. Figure R9 below shows a representative example of our warping results, which demonstrate high-fidelity correspondence between warped data and the reference atlas.

While density quantifications across entire Allen Brain structures can provide overall impressions of the data, we believe they drastically oversimplify the biology. Indeed, Allen Brain structures span large parts of the brain (as the referee notes in Point 7), and rarely correspond neatly to behaviorally relevant functional units. This is exemplified in Figure R10 below, which illustrates 1) the highly restricted nature of Fos induction patterns in the VMH and the MPO/LPO, and 2) the poor correspondence between cohesive Fos induction patterns and atlas region boundaries. Dorsomedial VMH (VMHdm) and MPO/LPO signals are strongest at the 3h recovery timepoint, and corresponding voxels are classified as Type 2 (sleep-correlated) by our clustering analyses (see Figure 1). Unsurprisingly, when we quantify Fos+ cells across the entire LPO or MPO, we observe a fundamentally different pattern in which sleep-related signals are averaged out. In summary, voxel-based quantifications more faithfully capture the data and are more suited to isolate sleep or wake-related Fos signals than quantifications across entire Allen Brain structures.

645

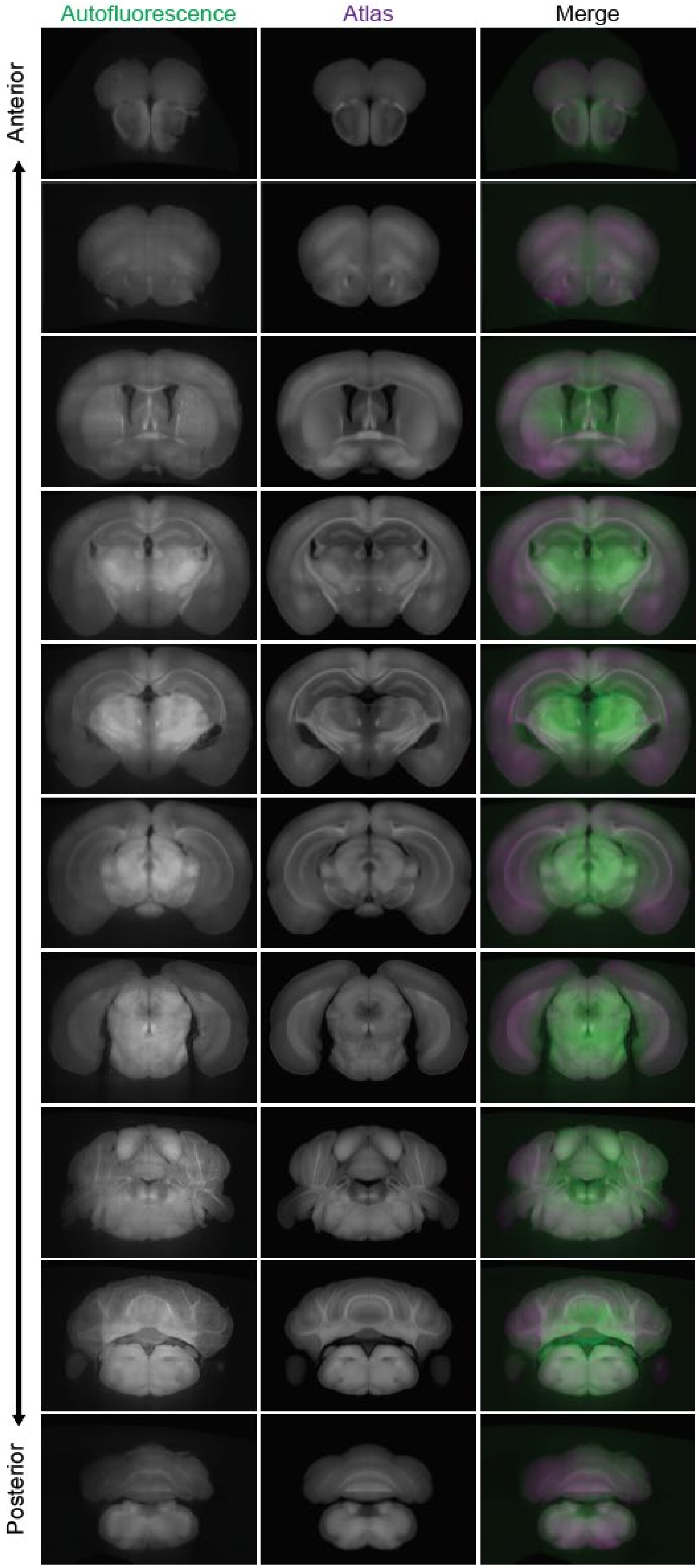

Figure R9: Representative brain warping results
Representative sample (Green, tissue autofluorescence) warped to an average mouse brain template (Magenta). Example coronal planes are shown in anterior to posterior order.

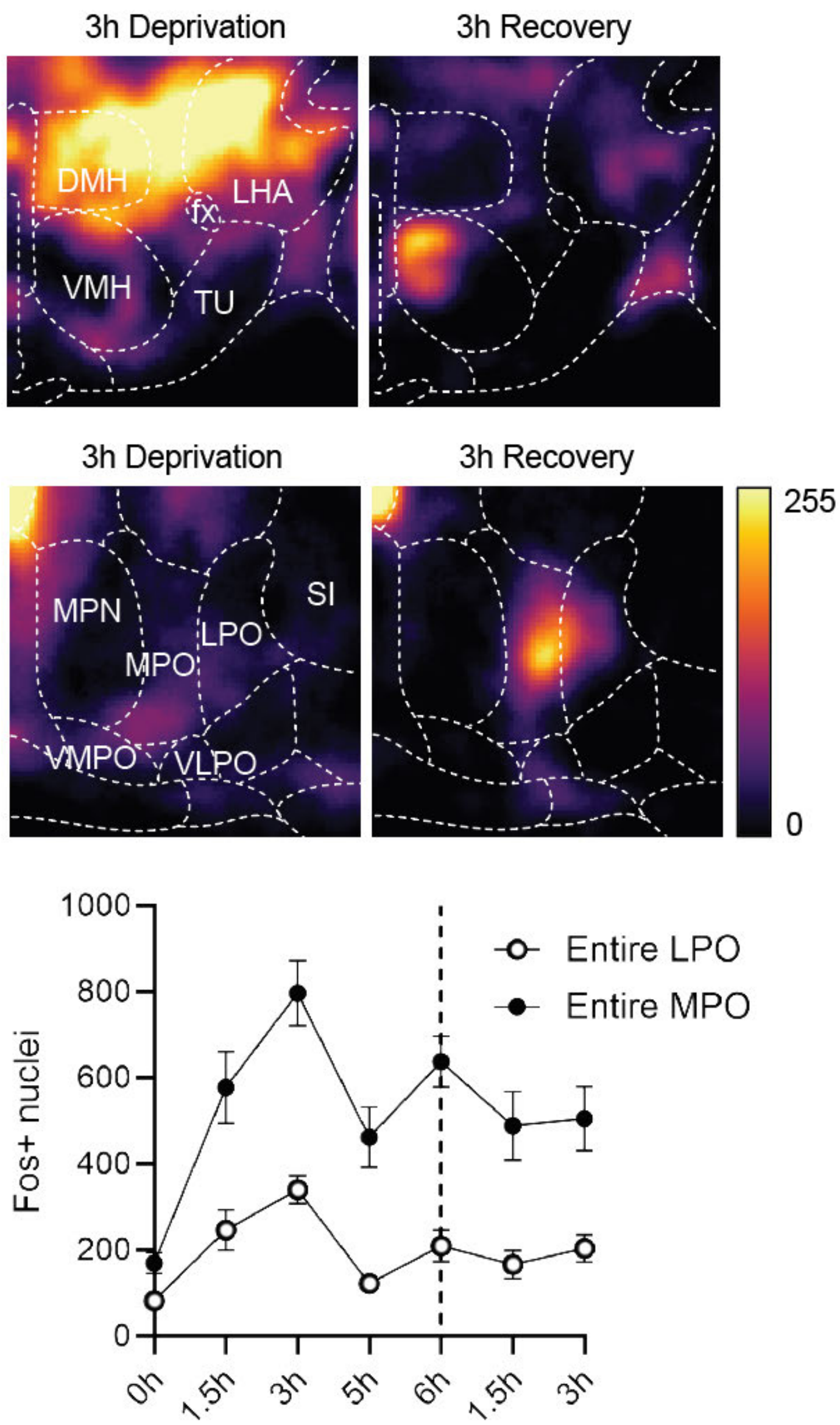

Figure R10: Voxel vs region-based quantifications of Fos induction
Average voxel maps for 3h deprivation (left) and 3h recovery (right) timepoints, highlighting sub-region patterns for VMH and LHA (top row) and MPO/LPO (bottom row). Bottom panel, Fos+ nuclei detected for the entire LPO (open circles) or MPO (closed circles) across deprivation timepoints.

10. “Now about the different types of structures they identified. They report that Type 1 responses peaked 1.5-3h into sleep deprivation and decayed thereafter and that they accounted for the greatest proportion of the brain and were enriched in cortical areas. I understand that it corresponds to neurons active during wake. How they explain that the labeling of these structures decrease over time during sleep deprivation? If there are active during wake they should be cFos+ during all the sleep deprivation period and even a bit after since cFos expression is visible normally one hour after stimulation? In fact, I expect that Type 3 neurons are also containing wake active structures?”

Like the referee, we were intrigued by brain areas that were activated during wakefulness and at the beginning of sleep deprivation. We interpret these Type 1 response patterns as reflecting sensory experience. Activity-regulated genes such as Fos can exhibit transient upregulation even during prolonged sensory, electrical, or pharmacological stimulation (Kushinsky et al 2024; Bisler et al 2002; Zangenehpour & Chaudhuri 2002; Semba et al 2001; Bullitt, et al 1992; Tyssowski, et al 2018). Furthermore, Type 1 regions exhibit minimal activation in unperturbed animals (Figure 1d), suggesting that deprivation stimuli are required for strong Fos induction in these areas. We therefore do not believe that these neurons “should be Fos+ during all the sleep deprivation period.”

Type 1 regions contrast strikingly with Type 3 regions, which exhibit wake-correlated activation even in the absence of deprivation stimuli. We agree that Type 3 regions may also include regions that respond to or maintain wakefulness. Accordingly, we functionally tested different Type 3 regions and found that the MR and aMPO promoted sleep (Figure 2), while the LHA promoted wakefulness (Extended Data Figure 7).

11. “Here again, I would like to see the statistical tests showing that Type 1 and Type 3 are really behaving differently.”

We have now added statistical tests to Figure 1d, which demonstrate that the three response types exhibit significantly different temporal response patterns.

12. “In fact, I would like to see a supplementary Table with all structures analyzed. What’s happening for example in the Dorsal raphe, the tuberomammillary nucleus and the locus coeruleus, three classical wake systems?”

A table summarizing hundreds of Allen Brain subdivisions cannot sufficiently describe the data (see response to Point 9 above), and would be difficult to present for our numerous timepoints and conditions.

As an alternate solution, we have created a new web resource (https://sleep-wake-atlas.scicore.unibas.ch/) that allows users to explore the whole-brain fos data from multiple perspectives, including:

1) Examining Fos responses over time for all experiments
2) Querying Fos responses by brain regions of interest
3) Querying Fos responses by temporal patterns of interest
4) Examining hierarchical clustering of Fos response profiles

We hope this new resource can provide a more comprehensive view of the data, particularly for regions that are not central to this manuscript.

13. “Indeed Fig 1 is not very informative and if Type 1 structures are quite big, the aspect of Type 2 and Type 3 structures is really puzzling. For example you have multiple Type 3 spots inside the caudate nucleus. This is really strange. Indeed, it seems impossible that the analysis made in multiple mice and conditions can lead to such spotty positive spots.”

As discussed above in Points 9-10 and the corresponding references, voxel-based analyses can resolve anatomical differences with greater resolution than cell counts across brain atlas subdivisions. The emergence of novel sub-region patterns across multiple animals and conditions highlights the strengths of our approach, and generates exciting questions for future experiments.

14. “I wonder whether the authors did differentiate NREM and REM sleep in their analysis? It would be important to know whether there is a strong REM or nonREM increase in their recovery period?”

Our sleep/wake analysis indeed distinguished between NREM and REM sleep states, and Extended Data Figure 1 demonstrates increased NREM sleep and delta power following sleep deprivation. In response to the referee comments, we have also now included REM sleep in Extended Data Figure 1.

15. “When you report line 74 that Type 3 structures activation is correlated with wake duration, I see no statistical analysis there. This is a crucial point since it is the basis of the demonstration. The same for Type 2 structures and the link with sleep, I don’t see the statistics I’m also surprised to see the LHAdl and VTA are type 3 structures. They indeed contain wake-system which rather inactive during NREM and orexin and dopamine neurons of the VTA are not known to increase their activity with sleep pressure?”

We apologize for the confusion. Extended Data Figure 3c was not correctly referenced for this statement, and this has now been corrected. Extended Data Figure 3c shows a Pearson correlation analysis for the undisturbed circadian experiment. Voxel intensity correlations with wake duration were calculated for a 2h time window preceding each experimental timepoint. This analysis highlighted significantly wake-correlated Type 3 regions including the MR and aMPO, as well as wake anti-correlated Type 2 regions such as the LPO and ventral medulla.

16. “Concerning the TRAP experiment, the authors state that the control condition is a rested control (line 405) with injection in undisturbed animals in the light phase but in Figure 2 , they state recovery TRAP? Also the number of mice with control TRAP injection is only of three. This is not sufficient for such experiments.”

We apologize for any confusion. We have added an additional line to the methods explaining that “for Recovery-TRAP, 4-OHT was administered 3h after the end of 6h sleep deprivation.” Furthermore, we have repeated these experiments with additional animals, bringing the sample size to n=11 for Deprivation TRAP and n=8 for Recovery TRAP. The conclusions remain unchanged.

17. “I’m also lacking the statistical differences in the hour by hour figure 2e, k and in all the other figures. I don’t get what happens with REM? It is not modified?”

We have now added hour-by-hour statistical comparisons to Figure 2e and k, as well as Figure 5 and Extended Data Figure 11. In figure 2, REM sleep exhibits trends in both MR and aMPO activation, but as they are statistically insignificant, we do not draw strong conclusions about MR/aMPO activation and REM sleep in this paper.

18. I would like to see two hypnograms showing the control and a deprived TRAP stimulation mice to have an idea of the distribution of each state.

As requested, we have created a new Extended Data Figure 4 with hypnograms for the MR and aMPO TRAP experiments.

19. Figure 2i, the box is rather centered on the nucleus accumbens than the MPO. It is not clear what area the photo is showing since there is no landmark.

We have added anatomical landmarks to Figure 2i-j, which clearly show TRAP labeling in aMPO rather than the nucleus accumbens. For additional clarity, we now include injection maps in Extended Data Figure 5.

20. Interestingly, the authors also studied the effect of LHA stimulation. Strangely here that don't specify that it is the dorsolateral portion of it although their square in Extended Figure 5a is dorsal in the HLA. Anyway in B the photo show a quite extensive transfection across the entire HLA. Further, the orexin neurons seem transfected but not the MCH ones. They then stimulated the TRAPed neurons and obtained wakefulness and concluded that some Type 3 structures are not regulating sleep but wake and Type 1 neurons as I indeed discussed above. My question here is whether it is because there are not selective to the dorsolateral portion or that the nomenclature is in fact covering Type 1 neurons?

We have now included anatomical landmarks and injection maps, which more explicitly demonstrate dorsolateral enrichment of LHA TRAP cells (Extended Data Figure 5b-d).

21. For the Kir experiments, I dont see in the Fig3 the significance related to line 127. Overall, the experiments quite convincingly show that MR and MPO might play a role in sleep drive. However I'm missing more detailed analysis with the hM4Di experiments since the effect on sleep should be more pronounced that after the permanent inactivation using Kir. We know that CNO effect last 6h so I wonder what would happen if they inject CNO at the end of the deprivation? This would directly prove the involvement of the median raphe and these experiments are necessary also for the MPO.

We are glad the referee also concludes "the experiments quite convincingly show that MR and MPO play a role in sleep drive."

Our Fos data and TRAP experiments indicate that MR deprivation-responsive cells are wake-active rather than sleep active (Figure 1, Figure 2, Figure 3, Extended Data Figure 3, Extended Data Figure 5). To test whether this wake-related activity is required for increased sleep drive, we acutely inhibited TRAP cells during deprivation (Figure 3o-p, Extended Data Figure 7). Indeed, we found that TRAP cell activity is required during wakefulness for increased sleep drive, as evidenced by strongly reduced sleep attempts.

In contrast, "CNO injection at the end of deprivation" would overlap with the naturally occurring decline in activation during the recovery period, thus making it difficult to interpret the results.

22. The authors then look at the efferents and afferents of the TRAPed neurons of the median raphe. Then, they stimulate the TRAPed neurons and show that some of the structures targeted by the median raphe contained a larger number of cFos and others less suggesting that both excitatory and inhibitory neurons compose the TRAPed neurons. Since they show that some median raphe neurons are GABA and others are serotonin, do they believe that serotonin neurons are excitatory?

Based on the data in Figure 4, we do not draw any conclusions regarding whether MR serotonergic cells are inhibitory or excitatory. A more detailed understanding would require characterization of postsynaptic neuron identity and serotonin receptor type. The functions of serotonin release in target areas is an exciting question, but we believe it is beyond the scope of the present study.

23. They should use LHb for lateral habenula for clarity. It is also a bit surprising that they found a decrease in cFos in the VI since it is a oculomotor nucleus. They should confirm that it is not a nearby structure.

We have changed the lateral habenula label to "LHb" as requested.

As for VI, both Figure 4 and the additional data below demonstrate MR projections to VI, as well as suppression of Fos in VI. We now include this in an extended data figure.

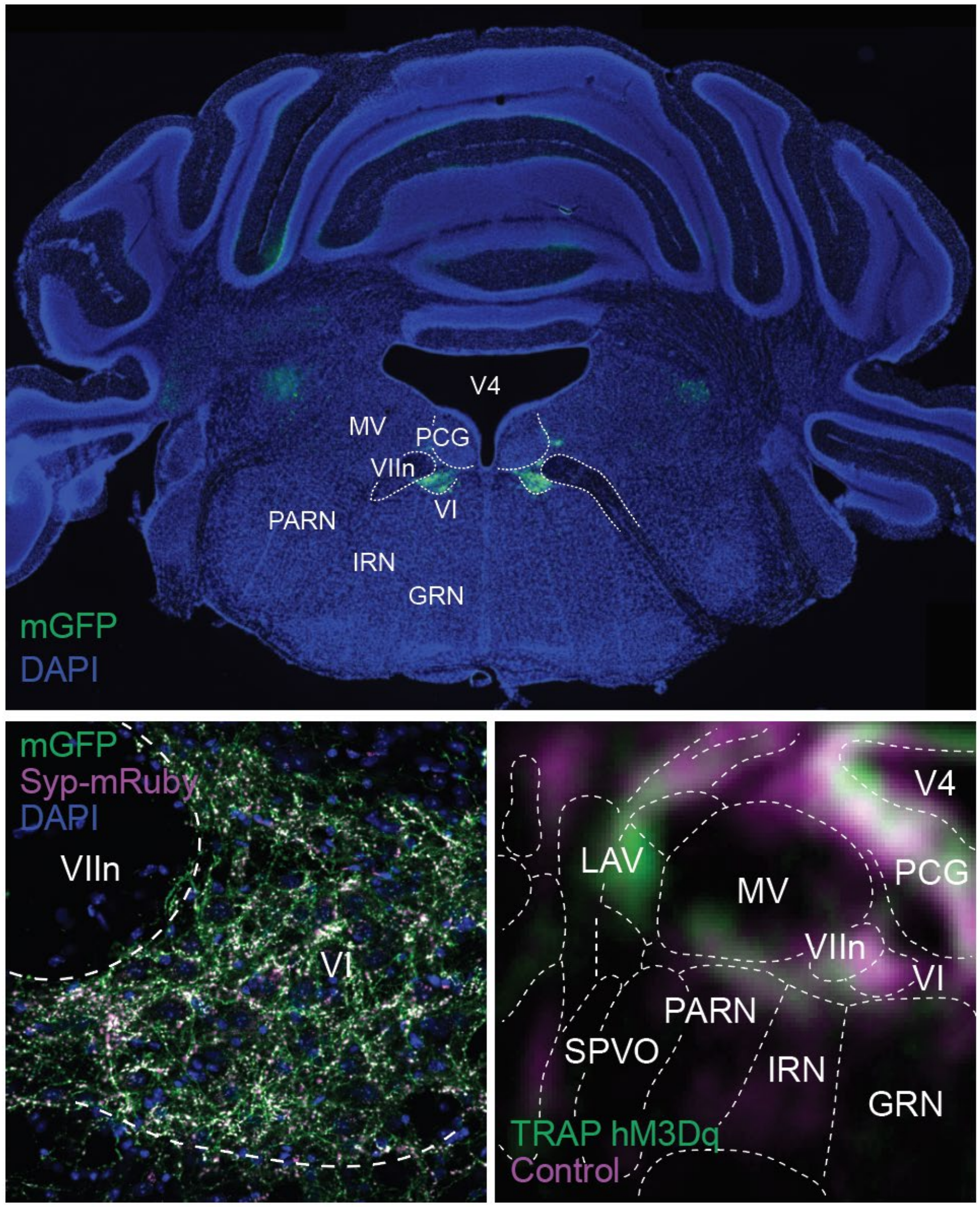

Figure R11 (Extended Data Figure 10): MR Deprivation TRAP cells project to the abducens nucleus
Top, Overview of MR Deprivation TRAP cell axons labeled by AAV-hSyn-DIO-mGFP-2A-Synaptophysin-mRuby. Only the mGFP (Green) channel is shown for clarity. Bottom left, Higher magnification image with colocalization of Syp-mRuby (magenta) and mGFP (green). Bottom right, Chemogenetic activation of MR Deprivation TRAP cells suppresses Fos in VI. Green, Fos heatmap for TRAP activation. Magenta, Fos heatmap for No-TRAP control.

24. “Then the authors made triple staining using vGAT, vGlut2 and serotonin to determine the neurotransmitter content of the median raphe TRAPed cells and demonstrated that the cells are mostly serotonin or vGAT but not vGlut2 positive. They don’t determine whether serotonin neurons are also GABAergic but it seems it has been shown in a previous paper and they should specify it if it is indeed the case.”

Indeed, previous work has demonstrated that “the MR includes three non-overlapping cell types with distinct neurotransmitter identities. GABAergic Vgat+ cells represent 60% of MR cells, while glutamatergic Vglut2+ cells and serotonergic cells respectively comprise ~25% and ~10%.” We concurrently cite the two relevant references: Sos et al 2017 and Szonyi et al 2019. We hope this provides additional clarity.

25. “Then, the authors used vgat-cre, vglut2-cre and Sert-Cre lines to stimulate and inhibit by chemogenetic the three populations of median raphe neurons. They nicely show that serotonergic and GABAergic neurons induce the same effect that the TRAPed neurons indicating that these two populations are responsible for promoting sleep after sleep deprivation. In contrast, glutamatergic neurons are responsible for inducing wakefulness.”

We thank the referee for their positive comments.

26. “…they used Kir to inhibit both the serotonergic and GABAergic neurons of the median raphe chronically. They indeed observed a 70% decrease of NREM sleep compared to control and increased mean wake bout length over 2-fold and this during nearly one month. Further, delta power increased is reduced after wake periods. It would be really interesting to look at the effects on behavior of such long lasting insomnia.”

As the referee suggests, the MR Kir2.1 animals provide a unique opportunity to examine the consequences of chronic sleep loss. We therefore conducted additional behavioral experiments, and found that Kir2.1 animals are more mobile, but do not exhibit significantly elevated anxiety-like behaviors in the open field or elevated plus maze assays (see Figure R3 above). Kir2.1 animals also spent more time interacting with wood blocks placed in the homecage, as evidenced by the increased surface abrasion and reduced volume of blocks from Kir2.1 animals relative to controls (see Figure R4 above). This suggests that Kir2.1 animals can maintain high behavioral engagement despite their chronic sleep loss, a hypothesis additionally supported by their increased mobility in open field and plus maze assays (Figure R3), as well as their sustained interaction with novel objects during sleep deprivation (Figure 6).

Because sleep deprivation can disrupt learning and memory processes, we also trained Kir2.1 animals in a contextual fear conditioning task, in which mice learn to associate a specific environment with an aversive experience (foot shocks, Figure R5a-b). Memory strength and long-term persistence were assessed by reintroducing mice to the same conditioning environment at recent (1 day) or remote (14 days) timepoints, and measuring freezing behavior and locomotion in the absence of shocks. Interestingly, all Kir2.1 animals were able to encode long-lasting memories, and exhibited freezing levels significantly higher than naïve (pre-shock) baseline up to 14 days after conditioning (Figure R5f). Memory strength was modestly weaker than in controls during recent recall, a phenotype that resembles the effects of 5-6h sleep deprivation or chronic partial sleep restriction (Graves et al 2003, Hagewoud 2010, Rothman et al 2013; Kincheski et al 2017). In contrast, 72-96h sleep deprivation severely compromises or even abolishes freezing (McDermott et al 2003, Ruskin et al 2004; Rossi et al. 2014), and thus affects animals more severely than MR inhibition. Furthermore, freezing behavior did not significantly differ from controls when tested 14 days after training. By comparison, the behavioral consequences of sleep loss progressively worsen over the course of weeks in humans, with as much as 15-fold worse performance in vigilance tasks during 1-2 weeks of sleep restriction (Belenky et al 2003, Van Dongen et al 2003). Overall, the encoding and long-term storage of associative memories remains intact in Kir2.1 animals, without the profound deficits of other dramatic interventions.

Reduced freezing behavior of Kir2.1 animals during recent recall was not due to reduced shock sensitivity or lower arousal during training. On the contrary, Kir2.1 animals reacted to shocks with greater vigor, traversing greater horizontal distances and jumping more frequently in response to each shock (Figure R5c-d). Furthermore, Kir2.1 animals were more mobile during both training and recall (Figure R5e). Indeed, reduced freezing during recent recall was primarily due to increased exploration when Kir2.1 mice were first reintroduced to the training context (Figure R5g-i). Kir2.1 animals thus perform as if highly aroused, but successfully encode memories without the severe impairments that accompany high doses of stimulants such as amphetamine and modafinil, which severely degrade recall (Shuman et al 2010, Wood et al 2010).

In summary, our behavioral analyses suggest that Kir2.1 animals can maintain high arousal and behavioral engagement despite their severe and long-term sleep loss. They can also form associative memories, without the severe defects observed in animals subjected to multi-day sleep deprivation protocols or high-dose stimulants. These observations further distinguish MR inhibition from other manipulations that strongly reduce sleep. For example, virally mediated Calcineurin Aα knockout reduces sleep by ~50%, but knockout animals exhibit pronounced anxiety-like behavior and completely abolished

contextual and cued fear memory recall (Yin et al 2025). Lastly, our observations corroborate the idea that MR inhibition compromises the detection or buildup of sleep drive (Fig 6). This is likely fatal when sleep loss exceeds 70%, as evidenced by the ~17% lethality of the Kir2.1 manipulation (see Extended Data Figure 14e-f). However, surviving Kir2.1 animals can maintain highly functional wakefulness for extended time periods without corresponding increases in sleep drive, and apparently without severe sleep deprivation-related behavioral deficits.

Our new behavioral data is now included in Extended Data Figures 14, 15, 16, and 17.

27. By the way, the authors did not discuss the possibility that median raphe neurons dysregulation could play a role in insomnia and they should I believe.

We thank the referee for making this connection. While it remains unclear how the MR contributes to idiopathic insomnia, our manipulations clearly distinguish MR inhibition from other insomnia models. According to the referee's suggestion, we now discuss the possibility that MR manipulations may be used to study sleep loss in the following discussion: "Our results diverge from other manipulations that decrease sleep by targeting either sleep-promoting or arousal-promoting systems. On one hand, perturbations that decrease sleep amount do not necessarily reduce sleep drive. For example, LPO-lesioned animals sleep less but initiate sleep more frequently, suggesting intact sleep propensity[37]. Similarly, nNos knockout mice sleep less but are excessively sleepy, particularly during sleep deprivation.[75] These perturbations thus compromise normal sleep and the dissipation of sleep drive, whereas the MR and aMPO likely contribute to the buildup or detection of sleep drive. On the other hand, manipulations that increase arousal typically increase sleep drive as well. For example, activating specific cell types in the ventral tegmental area, lateral hypothalamus, preoptic area, or basal forebrain strongly increases high arousal wakefulness[66,76-78], as does systemically administering cocaine or methamphetamine[79,80]. All of these manipulations produce compensatory increases in NREM sleep duration and/or delta power. In contrast, animals with inhibited MR/aMPO cells exhibit fewer attempts to sleep, less rebound sleep, and lower delta power accumulation despite increased wake time (Figs. 3 and 6). Much of this time is spent in high arousal wakefulness, but with lower than expected behavioral or EEG markers of sleep debt. Behaviorally, animals with chronic MR inhibition were able to maintain highly engaged wakefulness, and successfully formed new memories despite severe and long-term sleep reductions (Extended Data Figs. 15-17). MR manipulations may thus provide a novel context to study the long-term effects of sleep loss and high arousal.

28. Also missing from the paper is recording of the median raphe neurons to show that their activity is different than that of classical wake systems.

Our data indicate that MR GABAergic and serotonergic neurons are activated by wakefulness, but are sleep-promoting (rather than wake-promoting). In response to the referee's comment, we conducted electrophysiology experiments to examine how sleep deprivation leads to activation of MR GABAergic and serotonergic cells. Specifically, we compared the intrinsic properties of both cell types in rested and sleep deprived mice using whole-cell patch clamp recordings (See Figure R6 and R7 above, or the new Figure 5 and Extended Data Figure 13). Strikingly, sleep deprivation depolarized the membrane potential of GABAergic cells and lowered their firing thresholds. Accordingly, sleep deprived animals exhibited a greater proportion of GABAergic cells that spontaneously fired action potentials, and action potential firing rates were increased relative to rested controls (Figure R6a and Figure R7g). Consistent with these effects, rheobase was also lower in sleep deprived animals (Figure R7f). In contrast, serotonergic neurons did not exhibit significantly altered resting membrane potential or firing threshold (Figure 5q). Sleep deprivation thus shifts neuronal intrinsic properties towards a more excitable state specifically in MR GABAergic cells. Separate mechanisms likely promote activation of serotonergic neurons, and remain to be identified in future studies.

Our new results indicate that sleep deprivation triggers cell type-specific plasticity mechanisms in the MR, and further distinguish MR neuronal activity from that of "classical wake systems" like the LC, LHA, or parabrachial nucleus (PB). LC norepinephrine transmission becomes severely attenuated during sleep deprivation or naturally occurring long wake bouts (Bellisi et al 2016; Sima et al 2024). Likewise, the wake-promoting effects of LHA orexin neuron stimulation or parabrachial neuron activation diminish with

increased sleep pressure (Carter et al 2009, Qiu et al 2016). The activity or efficacy of these wake-promoting cells thus declines during sleep deprivation. In contrast, MR GABAergic cells exhibit increased excitability during sleep deprivation, which likely contributes to their sleep-promoting functions.

29. I have also some concern on the identification of the median preoptic area and even of its definition and this should be corrected.

We assume the referee is referring to the aMPO, and have now added additional anatomical landmarks to Figure 2, Extended Data Figure 5, and Extended Data Figure 18. These revisions further clarify how we define the aMPO.

30. In summary, the results presented in the present paper are quite impressive and identify a full new target for sleep induction of a different kind that previously identified systems. As described above I have some criticisms in particular concerning the neuroanatomical analysis. Although it constitutes a huge work the way it is analyzed and presented is not optimal and even the interpretation might not be accurate since most regions contain mixed populations. The median raphe is even a good example since it contains neurons with opposite role although they seem to be all active during wakefulness. Also missing from the paper is recording of the median raphe neurons to show that their activity is different than that of classical wake systems. I have also some concern on the identification of the median preoptic area and even of its definition and this should be corrected. Overall despite these concerns I believe the report is of great interest for understanding how sleep is regulated.

We thank the referee for their enthusiasm and positive feedback. Thanks to their comments, we believe the revisions, clarifications, and new experiments summarized above have greatly improved the manuscript.

REFEREE #3 (Remarks to the Author):

The manuscript by Joo and colleagues investigates the neural circuitry underlying sleep drive after prolonged wakefulness. Prior work has identified direct sleep state regulation circuits, and the neural drivers of sleep pressure are still not well understood. This manuscript demonstrates that specific cell types within the median raphe have distinct and substantial effects on sleep/wake behavior, and specifically the buildup of sleep pressure during wakefulness. This is an exciting topic and the results are interesting and well-written. There are some questions about the data and analyses. The whole brain analysis in particular is an important part of the results but has weaknesses that prevent interpretation of the results.

We thank the referee for their enthusiasm and their thoughtful questions below.

Note: For clarity, we have added numbers to each of the referee's comments.

1. The Fos data in Fig 1 are interesting and a key part of the paper, but represent a qualitative rather than quantitative assessment of activity, and it is not currently possible to assess which parts of these results are robust. The way the data are analyzed is statistically circular, where clustering is performed and then the difference between the clusters is displayed, which can produce misleading results. It's therefore not clear which specific brain areas are showing real effects vs. noise. To draw a rigorous conclusion in Fig 1., the distinct spatiotemporal patterns would need to be quantitatively compared, and statistically testing data that are already separated by clustering is problematic unless a statistically independent method is used to define the clusters.

Based on the referee's comments, we assessed the robustness of our activity mapping observations using a less biased, unsupervised clustering approach. In our initial submission, our strategy relied on manual selection of brain regions with circadian, wake, or sleep-correlated patterns, followed by clustering and label propagation to the rest of the brain. In our updated approach, we first automatically segment all voxels within each experiment (without any manual selection) according to peak activation timepoints, followed by hierarchical clustering into distinct response types (Figure R1 above). This analysis likewise allowed us to segment the brain into wake or sleep-correlated response types, and reached identical conclusions as our previous approach.

These conclusions are further strengthened by our Pearson correlation analyses for the undisturbed circadian experiment (Extended Data Figure 3c). Voxel intensity correlations with wake duration were calculated for a 2h time window preceding each experimental timepoint. This analysis highlighted significantly wake-correlated regions including the MR and aMPO, as well as wake anti-correlated regions such as the LPO and ventral medulla (both of which promote sleep). Notably, these wake and sleep-correlated regions largely corroborated the Type 3 and Type 2 responses identified by our clustering approach.

2. The methods for the whole-brain analysis need more detail to enable replication. How large was the blurring kernel? Where were the manually selected index voxels and how were they chosen? An analysis to determine whether a region's inclusion/weighting in a given cluster is statistically robust is needed.

We have updated the methods with more detail regarding data acquisition, processing, and analysis. The updated methods now include additional details on how images were pre-processed, and how blur was applied to Fos signal (Gaussian blur with sigma = 2.25). Our new clustering approach no longer relies on manually selected index voxels, instead segmenting the brain based on a peak timepoint heuristic followed by hierarchical clustering. We also now include supplemental data files to show clear separation of voxels in PCA space based on peak activation timepoints. Furthermore, whole brain data can now be browsed at an online resource (https://sleep-wake-atlas.scicore.unibas.ch/), and all analysis-related code is available at https://gitlab.com/ceda-unibas/sleep-brain-atlas.

3. Fig 1e states 'Representative data for 8 animals' - how were these data selected? What do the error shaded bars represent? It would be better to show all the data with full statistics.

Implanting all ~160 animals with EEG electrodes was prohibitively labor intensive. Accordingly, rather than show EEG data for all animals in the circadian/deprivation experiments in Figure 1, we show

data for 8 representative animals to demonstrate the effectiveness of our sleep deprivation methods (Also see Extended Data Figure 1).

The error bars represent standard error of the mean, and this information has now been added to the figure legend. We apologize for this oversight.

4. It is not completely clear in Fig 2, but it seems there is no control with mice receiving CNO without cell-type-specific activation. CNO can directly induce sedative-like effects and could be expected to increase NREM sleep behavior, and a control statistically comparing matched animals receiving CNO and without the Deprivation/Recovery-TRAP is not shown. Extended data Figure 4d suggests this non-specific CNO effect could be present since the rested mice also show a trend towards more NREM sleep with CNO; to use this condition as a control, the difference between different CNO groups would need to be reported, but the statistical comparison between the rested and sleep-deprived conditions is missing (Niewenhuis et al., Nature Neuro, 2011)

We have now added a statistical comparison between the rested control TRAP and deprivation TRAP conditions in Extended Data Figure 5, which demonstrates significantly higher NREM sleep induction in deprivation vs control. In addition, we now include equivalent data for TRAP mice without hM3Dq expression, which likewise show no significant effects of CNO at the dose used throughout our manuscript (1 mg/kg). These results agree with previous studies demonstrating that 1 mg/kg CNO does not affect sleep duration in wild-type animals (Ferrari et al 2022, Grabau et al 2025).

5. n animals missing from some figures; e.g. Figure 3o,p;

We have corrected this oversight, and now report the number of animals in Figure 3o-p (n=8 animals).

6. The control data in Figure 5m vs Figure 5g show very different NREM behavior patterns (top panels); the difference in the control groups is as large as the stated effect size of the CNO. Why are these groups so different, is this reflecting that activation vs. inhibition experiments were done in different phases (light/dark) for different cell types? If so why were the manipulations switched in phase across experiments, given that the effects of each cell type were not known in advance?

The shading in the time plots indeed indicates manipulations during the dark phase as opposed to light phase, hence the contrasting sleep proportions in control groups between Figure 5m and 5g. We apologize for any lack of clarity. In response to the referee's comments, we have added more detail to the figure legend.

For most experiments, we compared the effects of chemogenetic manipulation during both the light and dark phases. For simplicity, we initially presented only dark phase or light phase results, to emphasize how our manipulations can override normal sleep/wake behavior. We now present all data for light/dark phases in the new Extended Data Figure 11.

- what is the n in Figure 6h, how many mice did these bouts come from? Why are there so many fewer wake bouts from the Kir2.1 mice even though Figure 6d shows they are awake more than controls? Were data excluded prior to this calculation?

To evaluate the relationship between wake duration and NREM Delta power, we used a previously published approach (Krone et al 2021), focusing on consolidated ≥ 15 minute wake episodes that were flanked by at least 10-15 minute sleep bouts. While data was pooled across all animals during the 5 day baseline period (Control n=8 animals, Kir2.1 n=6 animals), Kir2.1 animals exhibited very few wake episodes fitting these criteria due to their profound reduction in sleep.

Based on the referee's comments, we performed additional experiments to replicate our initial observations, bringing the total number of samples to 20 control animals and 17 Kir2.1 animals. Figure 6h now includes updated sample sizes as follows: n= 272 wake bouts from control animals, n=42 wake bouts

from Kir2.1 animals (Compared to 116 control bouts and 13 Kir2.1 bouts in our initial submission). Our conclusions remained unchanged.

- The discussion states the MR cell-type-specific effects are the key regulator of sleep drive, but based on Figure 1 it seems unclear that it's justified to state that sleep drive is specific to this one region; many areas are flagged and it seems also plausible that this is a systems-level process of which MR is one important part, but which doesn't necessarily have only one area as a single switch. While inhibiting MR is proposed to specifically reduce sleep drive and is contrasted with areas that cause animals to be 'excessively sleepy' in the discussion, and it's suggested that these kir2.1 animals are not sleep-deprived, whether this manipulation actually removes sleepiness from a cognitive/functional perspective is not part of this paper. This does not require new experiments but could be considered in the discussion.

We agree with the referee that the MR likely acts as part of a complex system that regulates sleep homeostasis, rather than as the sole master regulator. We now explicitly state this in the discussion.

Like the referee, we also wondered whether Kir2.1 animals exhibit behavioral/functional signs of sleep deprivation. We therefore conducted additional behavioral experiments, and found that Kir2.1 animals are more mobile, but do not exhibit significantly elevated anxiety-like behaviors in the open field or elevated plus maze assays (see Figure R3 above). Kir2.1 animals also spent more time interacting with wood blocks placed in the homecage, as evidenced by the increased surface abrasion and reduced volume of blocks from Kir2.1 animals relative to controls (see Figure R4 above). This suggests that Kir2.1 animals can maintain high behavioral engagement despite their chronic sleep loss, a hypothesis additionally supported by their increased mobility in open field and plus maze assays (Figure R3 above), as well as their sustained interaction with novel objects during sleep deprivation (Figure 6I).

Because sleep deprivation can disrupt learning and memory processes, we also trained Kir2.1 animals in a contextual fear conditioning task, in which mice learn to associate a specific environment with an aversive experience (foot shocks, Figure R5a-b). Memory strength and long-term persistence were assessed by reintroducing mice to the same conditioning environment at recent (1 day) or remote (14 days) timepoints, and measuring freezing behavior and locomotion in the absence of shocks. Interestingly, all Kir2.1 animals were able to encode long-lasting memories, and exhibited freezing levels significantly higher than naïve (pre-shock) baseline up to 14 days after conditioning (Figure R5f). Memory strength was modestly weaker than in controls during recent recall, a phenotype that resembles the effects of 5-6h sleep deprivation or chronic partial sleep restriction (Graves et al 2003, Hagewoud 2010, Rothman et al 2013; Kincheski et al 2017). In contrast, 72-96h sleep deprivation severely compromises or even abolishes freezing (McDermott et al 2003, Ruskin et al 2004; Rossi et al. 2014), and thus affects animals more severely than MR inhibition. Furthermore, freezing behavior did not significantly differ from controls when tested 14 days after training. By comparison, the behavioral consequences of sleep loss progressively worsen over the course of weeks in humans, with as much as 15-fold worse performance in vigilance tasks during 1-2 weeks of sleep restriction (Belenky et al 2003, Van Dongen et al 2003). Overall, the encoding and long-term storage of associative memories remains intact in Kir2.1 animals, without the profound deficits of other dramatic interventions.

Reduced freezing behavior of Kir2.1 animals during recent recall was not due to reduced shock sensitivity or lower arousal during training. On the contrary, Kir2.1 animals reacted to shocks with greater vigor, traversing greater horizontal distances and jumping more frequently in response to each shock (Figure R5c-d). Furthermore, Kir2.1 animals were more mobile during both training and recall (Figure R5e). Indeed, reduced freezing during recent recall was primarily due to increased exploration when Kir2.1 mice were first reintroduced to the training context (Figure R5g-i). Kir2.1 animals thus perform as if highly aroused, but successfully encode memories without the severe impairments that accompany high doses of stimulants such as amphetamine and modafinil, which severely degrade recall (Shuman et al 2010, Wood et al 2010).

In summary, our behavioral analyses suggest that Kir2.1 animals can maintain high arousal and behavioral engagement despite their severe and long-term sleep loss. They can also form associative memories, without the severe defects observed in animals subjected to multi-day sleep deprivation protocols or high-dose stimulants. These observations further distinguish MR inhibition from other manipulations that strongly reduce sleep. For example, virally mediated Calcineurin Aα knockout reduces

sleep by ~50%, but knockout animals exhibit pronounced anxiety-like behavior and completely abolished contextual and cued fear memory recall (Yin et al 2025). Lastly, our observations corroborate the idea that MR inhibition compromises the detection or buildup of sleep drive (Fig 6). This is likely fatal when sleep loss exceeds 70%, as evidenced by the ~17% lethality of the Kir2.1 manipulation (see Extended Data Figure 14e-f). However, surviving Kir2.1 animals can maintain highly functional wakefulness for extended time periods without corresponding increases in sleep drive, and apparently without severe sleep deprivation-related behavioral deficits.

Our new behavioral data is summarized in Extended Data Figures 14, 15, 16, and 17.

References

- Affinati, et al. (2021). Cross-species analysis defines the conservation of anatomically segregated VMH neuron populations. *eLife*, *10*, e69065. https://doi.org/10.7554/eLife.69065

- Allen, et al. (2017). Thirst-associated preoptic neurons encode an aversive motivational drive. *Science (New York, N.Y.)*, *357*(6356), 1149–1155. https://doi.org/10.1126/science.aan6747

- Basheer et al. (1997). Effects of sleep on wake-induced c-fos expression. *The Journal of neuroscience : the official journal of the Society for Neuroscience*, *17*(24), 9746–9750. https://doi.org/10.1523/JNEUROSCI.17-24-09746.1997

- Becskei C, et al. (2009). Diet-derived nutrients mediate the inhibition of hypothalamic NPY neurons in the arcuate nucleus of mice during refeeding. Am J Physiol Regul Integr Comp Physiol. Jul;297(1):R100-10. doi: 10.1152/ajpregu.91014.2008.

- Belenky, et al. (2003). Patterns of performance degradation and restoration during sleep restriction and subsequent recovery: a sleep dose-response study. Journal of sleep research, 12(1), 1–12. https://doi.org/10.1046/j.1365-2869.2003.00337.x

- Bellesi, M., Tononi, G., Cirelli, C., & Serra, P. A. (2016). Region-Specific Dissociation between Cortical Noradrenaline Levels and the Sleep/Wake Cycle. *Sleep*, *39*(1), 143–154. https://doi.org/10.5665/sleep.5336

- Bodenmann, S., & Landolt, H. P. (2010). Effects of modafinil on the sleep EEG depend on Val158Met genotype of COMT. *Sleep*, *33*(8), 1027–1035. https://doi.org/10.1093/sleep/33.8.1027

- Buguet, A., Montmayeur, A., Pigeau, R., & Naitoh, P. (1995). Modafinil, d-amphetamine and placebo during 64 hours of sustained mental work. II. Effects on two nights of recovery sleep. *Journal of sleep research*, *4*(4), 229–241. https://doi.org/10.1111/j.1365-2869.1995.tb00173.

- Carter, M. E., Adamantidis, A., Ohtsu, H., Deisseroth, K., & de Lecea, L. (2009). Sleep homeostasis modulates hypocretin-mediated sleep-to-wake transitions. *The Journal of neuroscience : the official journal of the Society for Neuroscience*, *29*(35), 10939–10949. https://doi.org/10.1523/JNEUROSCI.1205-09.2009

- Chowdhury, S., Matsubara, T., Miyazaki, T., Ono, D., Fukatsu, N., Abe, M., Sakimura, K., Sudo, Y., & Yamanaka, A. (2019). GABA neurons in the ventral tegmental area regulate non-rapid eye movement sleep in mice. *eLife*, *8*, e44928. https://doi.org/10.7554/eLife.44928

- Donlea, J. M., Pimentel, D., & Miesenböck, G. (2014). Neuronal machinery of sleep homeostasis in Drosophila. *Neuron*, *81*(4), 860–872. https://doi.org/10.1016/j.neuron.2013.12.013

- Donlea, J. M., Pimentel, D., Talbot, C. B., Kempf, A., Omoto, J. J., Hartenstein, V., & Miesenböck, G. (2018). Recurrent Circuitry for Balancing Sleep Need and Sleep. *Neuron*, *97*(2), 378–389.e4. https://doi.org/10.1016/j.neuron.2017.12.016

- Edgar, D. M., & Seidel, W. F. (1997). Modafinil induces wakefulness without intensifying motor activity or subsequent rebound hypersomnolence in the rat. *The Journal of pharmacology and experimental therapeutics*, *283*(2), 757–769.

- Ferrari, L. L., Ogbeide-Latario, O. E., Gompf, H. S., & Anaclet, C. (2022). Validation of DREADD agonists and administration route in a murine model of sleep enhancement. *Journal of neuroscience methods*, *380*, 109679. https://doi.org/10.1016/j.jneumeth.2022.109679

- González, et al. (2012). Convergent inputs from electrically and topographically distinct orexin cells to locus coeruleus and ventral tegmental area. *The European journal of neuroscience*, *35*(9), 1426–1432. https://doi.org/10.1111/j.1460-9568.2012.08057.x

- Grabau, N. W., LeKites, V. W., Brousseau, E. K., Anzules, D. L., Ortiz-Feder, E. M., Vetrivelan, R., Todd, W. D., & Kroeger, D. (2025). Sleep modulation via Nucleus Accumbens A2AR neurons to probe cognition and behavior in mice. *Neuroscience*, *584*, 334–344. https://doi.org/10.1016/j.neuroscience.2025.08.015

- Graves, et al. (2003). Sleep deprivation selectively impairs memory consolidation for contextual fear conditioning. *Learning & memory (Cold Spring Harbor, N.Y.)*, *10*(3), 168–176. https://doi.org/10.1101/lm.48803

- Grzelka, K. *et al.* A synaptic amplifier of hunger for regaining body weight in the hypothalamus. *Cell Metab* **35**, 770–785 e775 (2023). https://doi.org/10.1016/j.cmet.2023.03.002

- Hagewoud, et al. (2010). A time for learning and a time for sleep: the effect of sleep deprivation on contextual fear conditioning at different times of the day. *Sleep*, *33*(10), 1315–1322. https://doi.org/10.1093/sleep/33.10.1315

- Hansen, et al. (2021). Whole-brain activation signatures of weight-lowering drugs. *Molecular metabolism*, *47*, 101171. https://doi.org/10.1016/j.molmet.2021.101171

- Harris, et al. (2005). A role for lateral hypothalamic orexin neurons in reward seeking. *Nature*, *437*(7058), 556–559. https://doi.org/10.1038/nature04071

- Harris, J. J., Kollo, M., Erskine, A., Schaefer, A., & Burdakov, D. (2022). Natural VTA activity during NREM sleep influences future exploratory behavior. *iScience*, *25*(6), 104396. https://doi.org/10.1016/j.isci.2022.104396

- Ji, et al. (2005). Effects of water deprivation and rehydration on c-Fos and FosB staining in the rat supraoptic nucleus and lamina terminalis region. *American journal of physiology. Regulatory, integrative and comparative physiology*, *288*(1), R311–R321. https://doi.org/10.1152/ajpregu.00399.2004

- Kempf, A., Song, S. M., Talbot, C. B., & Miesenböck, G. (2019). A potassium channel β-subunit couples mitochondrial electron transport to sleep. *Nature*, *568*(7751), 230–234. https://doi.org/10.1038/s41586-019-1034-5

- Kopp, C., Petit, J. M., Magistretti, P., Borbély, A. A., & Tobler, I. (2002). Comparison of the effects of modafinil and sleep deprivation on sleep and cortical EEG spectra in mice. *Neuropharmacology*, *43*(1), 110–118. https://doi.org/10.1016/s0028-3908(02)00070-9

- Krone, L. B., Yamagata, T., Blanco-Duque, C., Guillaumin, M. C. C., Kahn, M. C., van der Vinne, V., McKillop, L. E., Tam, S. K. E., Peirson, S. N., Akerman, C. J., Hoerder-Suabedissen, A., Molnár, Z., & Vyazovskiy, V. V. (2021). A role for the cortex in sleep-wake regulation. *Nature neuroscience*, *24*(9), 1210–1215. https://doi.org/10.1038/s41593-021-00894-6

- Lee, S. S. *et al.* Sleep need-dependent plasticity of a thalamic circuit promotes homeostatic recovery sleep. *Science* **388**, eadm8203 (2025). https://doi.org/10.1126/science.adm8203

- Lindberg, et al. (2013). Conditional viral tracing reveals that steroidogenic factor 1-positive neurons of the dorsomedial subdivision of the ventromedial hypothalamus project to autonomic centers of the hypothalamus and hindbrain. *The Journal of comparative neurology*, *521*(14), 3167–3190. https://doi.org/10.1002/cne.23338

- Liu, S., Liu, Q., Tabuchi, M. & Wu, M. N. Sleep Drive Is Encoded by Neural Plastic Changes in a Dedicated Circuit. *Cell* **165**, 1347–1360 (2016). https://doi.org/10.1016/j.cell.2016.04.013

- Liu, T. *et al.* Fasting activation of AgRP neurons requires NMDA receptors and involves spinogenesis and increased excitatory tone. *Neuron* **73**, 511–522 (2012). https://doi.org/10.1016/j.neuron.2011.11.027

- McDermott, et al. (2003). Sleep deprivation causes behavioral, synaptic, and membrane excitability alterations in hippocampal neurons. *The Journal of neuroscience : the official journal of the Society for Neuroscience*, *23*(29), 9687–9695. https://doi.org/10.1523/JNEUROSCI.23-29-09687.2003

- Meek, et al. (2016). Functional identification of a neurocircuit regulating blood glucose. *Proceedings of the National Academy of Sciences of the United States of America*, *113*(14), E2073–E2082. https://doi.org/10.1073/pnas.1521160113

- Qiu, M. H., Chen, M. C., Fuller, P. M., & Lu, J. (2016). Stimulation of the Pontine Parabrachial Nucleus Promotes Wakefulness via Extra-thalamic Forebrain Circuit Nodes. *Current biology : CB*, *26*(17), 2301–2312. https://doi.org/10.1016/j.cub.2016.07.054

- Qu, W. M., Huang, Z. L., Xu, X. H., Matsumoto, N., & Urade, Y. (2008). Dopaminergic D1 and D2 receptors are essential for the arousal effect of modafinil. *The Journal of neuroscience : the official journal of the Society for Neuroscience*, *28*(34), 8462–8469. https://doi.org/10.1523/JNEUROSCI.1819-08.2008

- Renier, et al. (2016). Mapping of Brain Activity by Automated Volume Analysis of Immediate Early Genes. *Cell*, *165*(7), 1789–1802. https://doi.org/10.1016/j.cell.2016.05.007

- Rorsman, H. O., Müller, M. A., Liu, P. Z., Sanchez, L. G., Kempf, A., Gerbig, S., Spengler, B., & Miesenböck, G. (2025). Sleep pressure accumulates in a voltage-gated lipid peroxidation memory. *Nature*, *641*(8061), 232–239. https://doi.org/10.1038/s41586-025-08734-4

- Rothman, et al. (2013). Chronic mild sleep restriction accentuates contextual memory impairments, and accumulations of cortical Aβ and pTau in a mouse model of Alzheimer's disease. *Brain research*, *1529*, 200–208. https://doi.org/10.1016/j.brainres.2013.07.010

- Ruskin, et al. (2004). Sleep deprivation impairs hippocampus-mediated contextual learning but not amygdala-mediated cued learning in rats. *The European journal of neuroscience*, *19*(11), 3121–3124. https://doi.org/10.1111/j.0953-816X.2004.03426.x

- Sarnataro, R., Velasco, C. D., Monaco, N., Kempf, A., & Miesenböck, G. (2025). Mitochondrial origins of the pressure to sleep. *Nature*, *645*(8081), 722–728. https://doi.org/10.1038/s41586-025-09261-y

- Sabatini, P. V., Wang, J., Rupp, A. C., Affinati, A. H., Flak, J. N., Li, C., Olson, D. P., & Myers, M. G. (2021). tTARGIT AAVs mediate the sensitive and flexible manipulation of intersectional neuronal populations in mice. *eLife*, *10*, e66835. https://doi.org/10.7554/eLife.66835

- Shuman, et al. (2009). Modafinil and memory: effects of modafinil on Morris water maze learning and Pavlovian fear conditioning. *Behavioral neuroscience*, *123*(2), 257–266. https://doi.org/10.1037/a0014366

- Shuman, T., Wood, S. C., & Anagnostaras, S. G. (2009). Modafinil and memory: effects of modafinil on Morris water maze learning and Pavlovian fear conditioning. *Behavioral neuroscience*, *123*(2), 257–266. https://doi.org/10.1037/a0014366

- Sima, J., Zhang, Y., Farriday, D., Ahn, A. Y., Lopez, E. R., Jin, C., Harrell, J., Darmohray, D., Silverman, D., & Dan, Y. (2024). Restoration of locus coeruleus noradrenergic transmission during sleep. *bioRxiv : the preprint server for biology*, 2024.07.03.601820. https://doi.org/10.1101/2024.07.03.601820

- Sos et al. (2017). Cellular architecture and transmitter phenotypes of neurons of the mouse median raphe region. Brain Struct Funct **222**, 287-299 https://doi.org/10.1007/s00429-016-1217-x

- Szonyi et al. (2019) Median raphe controls acquisition of negative experience in the mouse. *Science* **366**.https://doi.org/10.1126/science.aay8746

- Tan, et al. (2014). Ablation of AgRP neurons impairs adaption to restricted feeding. *Molecular metabolism*, *3*(7), 694–704. https://doi.org/10.1016/j.molmet.2014.07.002

- Tasaka, et al. (2018). Genetic tagging of active neurons in auditory cortex reveals maternal plasticity of coding ultrasonic vocalizations. *Nature communications*, *9*(1), 871. https://doi.org/10.1038/s41467-018-03183-2

- Thompson, et al. (2010). Molecular and anatomical signatures of sleep deprivation in the mouse brain. *Frontiers in neuroscience*, *4*, 165. https://doi.org/10.3389/fnins.2010.00165

- Thornquist, S. C., Pitsch, M. J., Auth, C. S. & Crickmore, M. A. Biochemical evidence accumulates across neurons to drive a network-level eruption. *Mol Cell* **81**, 675–690 e678 (2021). https://doi.org/10.1016/j.molcel.2020.12.029

- Van Dongen, et al. (2003). The cumulative cost of additional wakefulness: dose-response effects on neurobehavioral functions and sleep physiology from chronic sleep restriction and total sleep deprivation. *Sleep*, *26*(2), 117–126. https://doi.org/10.1093/sleep/26.2.117

- Vas, S., Casey, J. M., Schneider, W. T., Kalmar, L., & Morton, A. J. (2020). Wake-Promoting and EEG Spectral Effects of Modafinil After Acute or Chronic Administration in the R6/2 Mouse Model of Huntington's Disease. *Neurotherapeutics : the journal of the American Society for Experimental NeuroTherapeutics*, *17*(3), 1075–1086. https://doi.org/10.1007/s13311-020-00849-y

- Wang, et al. (2015). Collateral pathways from the ventromedial hypothalamus mediate defensive behaviors. *Neuron*, *85*(6), 1344–1358. https://doi.org/10.1016/j.neuron.2014.12.025

- Wang, et al. (2021). EASI-FISH for thick tissue defines lateral hypothalamus spatio-molecular organization. *Cell*, *184*(26), 6361–6377.e24. https://doi.org/10.1016/j.cell.2021.11.024

- Wood & Anagnostaras (2009). Memory and psychostimulants: modulation of Pavlovian fear conditioning by amphetamine in C57BL/6 mice. *Psychopharmacology*, *202*(1-3), 197–206. https://doi.org/10.1007/s00213-008-1185-9

- Wotus et al. (2007). Dehydration-induced drinking decreases Fos expression in hypothalamic paraventricular neurons expressing vasopressin but not corticotropin-releasing hormone. *American*

journal of physiology. Regulatory, integrative and comparative physiology, *292*(3), R1349–R1358. https://doi.org/10.1152/ajpregu.00304.2006

- Wu, G., Ma, T., Hancock, C. E., Gonzalez, S., Aryal, B., Vaz, S., Chan, G., Palarca-Wong, M., Allen, N., Chung, C. I., Shu, X., & Liu, Q. (2024). Opposing GPCR signaling programs protein intake setpoint in Drosophila. *Cell*, *187*(19), 5376–5392.e17. https://doi.org/10.1016/j.cell.2024.07.047

- Yamaguchi, et al. (2023). Dorsomedial and preoptic hypothalamic circuits control torpor. *Current biology* *33*(24), 5381–5389.e4. https://doi.org/10.1016/j.cub.2023.10.076

- Yan, R., Wei, D., Varshneya, A., Shan, L., Dai, B., Asencio, H. J., 3rd, Gollamudi, A., & Lin, D. (2024). The multi-stage plasticity in the aggression circuit underlying the winner effect. *Cell*, *187*(24), 6785–6803.e18. https://doi.org/10.1016/j.cell.2024.09.030

- Yang, et al. (2024). Phosphorylation of pyruvate dehydrogenase inversely associates with neuronal activity. *Neuron*, *112*(6), 959–971.e8. https://doi.org/10.1016/j.neuron.2023.12.015

- Yang, Y., Atasoy, D., Su, H. H., & Sternson, S. M. (2011). Hunger states switch a flip-flop memory circuit via a synaptic AMPK-dependent positive feedback loop. *Cell*, *146*(6), 992–1003. https://doi.org/10.1016/j.cell.2011.07.039

- Yatziv, et al (2021). Anesthesia in mice activates discrete populations of neurons throughout the brain. *Journal of neuroscience research*, *99*(12), 3284–3305. https://doi.org/10.1002/jnr.24950

- Yin, et al. (2025). Calcineurin governs baseline and homeostatic regulations of non-rapid eye movement sleep in mice. Proceedings of the National Academy of Sciences of the United States of America, 122(4), e2418317122. https://doi.org/10.1073/pnas.2418317122

- Yoshida, et al. (2006). Afferents to the orexin neurons of the rat brain. *The Journal of comparative neurology*, *494*(5), 845–861. https://doi.org/10.1002/cne.20859

- Yu, X., Li, W., Ma, Y., Tossell, K., Harris, J. J., Harding, E. C., Ba, W., Miracca, G., Wang, D., Li, L., Guo, J., Chen, M., Li, Y., Yustos, R., Vyssotski, A. L., Burdakov, D., Yang, Q., Dong, H., Franks, N. P., & Wisden, W. (2019). GABA and glutamate neurons in the VTA regulate sleep and wakefulness. *Nature neuroscience*, *22*(1), 106–119. https://doi.org/10.1038/s41593-018-0288-9

- Yu, X., Zhao, G., Wang, D., Wang, S., Li, R., Li, A., Wang, H., Nollet, M., Chun, Y. Y., Zhao, T., Yustos, R., Li, H., Zhao, J., Li, J., Cai, M., Vyssotski, A. L., Li, Y., Dong, H., Franks, N. P., & Wisden, W. (2022). A specific circuit in the midbrain detects stress and induces restorative sleep. *Science (New York, N.Y.)*, *377*(6601), 63–72. https://doi.org/10.1126/science.abn0853

- Zangenehpour & Chaudhuri, A. (2002). Differential induction and decay curves of c-fos and zif268 revealed through dual activity maps. *Brain research. Molecular brain research*, *109*(1-2), 221–225. https://doi.org/10.1016/s0169-328x(02)00556-9

- Zhang, S. X. *et al.* Stochastic neuropeptide signals compete to calibrate the rate of satiation. *Nature* (2024). https://doi.org/10.1038/s41586-024-08164-8

Response to Reviewer Comments

We thank each referee for their thoughtful feedback and their enthusiasm for our manuscript. The referees describe our revised paper as “substantially strengthened,” “particularly striking and compelling,” “elegant and comprehensive,” and “of broad interest to researchers investigating the regulation and functional significance of sleep,” (Referee 1); “amazing,” “new and interesting” (Referee 2); and “interesting and well-written,” “elucidat[ing] the neural mechanisms underlying sleep drive” (Referee 3).

We have further revised the manuscript based on the latest referee comments, leading to an even clearer presentation and discussion of our results. We hope these changes further strengthen the manuscript and address any remaining suggestions.

Below, we provide point-by-point responses to all referee comments.

Notes: Referee comments are in blue text, and responses are in black text. Referees 1 and 3 are presented first. Figures are presented in-line (Fig. R1-R4), and additional references are listed at the end of the document.

Referee #1 (Remarks to the Author):

The authors have substantially strengthened the manuscript by adding an extensive whole brain data set in the form of an online sleep–wake atlas, which will constitute a valuable community resource. They further show in new experiments that, although the animals with tonically inhibited GABA and 5HT cells exhibit markedly reduced sleep, this sleep loss does not measurably impair their cognitive performance, while prolonged sleep deprivation is associated with approximately 17% lethality (extended data Fig. 14e), perhaps underscoring the physiological cost of sustained sleep loss.

Fig. 6, documenting the prolonged genetic sleep deprivation paradigm, remains particularly striking and compelling.

The new electrophysiological data (Fig. 5p,q) are also informative: they demonstrate that MR GABAergic neurons become progressively more excitable with ongoing sleep deprivation, providing additional support for the idea that sleep drive is encoded by plasticity-based mechanisms. This is an emerging new idea in the field.

The authors have thoroughly and convincingly addressed all my previous concerns. Overall, this is an elegant and comprehensive study that will be of broad interest to researchers investigating the regulation and functional significance of sleep.

Happy to sign as: William Wisden

We thank Dr. Wisden for his strong support.

Referee #3 (Remarks to the Author):

The authors have addressed my comments and I congratulate them on the interesting and well-written paper, which elucidates the neural mechanisms underlying sleep drive.

One suggestion is that the authors could consider rephrasing the statements in the discussion that co-inhibition does not produce behavioral deficits, or perhaps adding context to this statement somewhere in the discussion. The statement gives the impression that manipulating these cells can fully remove the need to sleep, but since 17% of the animals receiving this manipulation died, it does cause behavioral deficits. It seems plausible that only the more cognitively robust animals survived to be tested on the behavioral tasks, which may bias the behavioral results somewhat. However this is just a discussion point for the authors to consider.

We thank the referee for their enthusiastic support and their thoughtful comments. We agree that our description of the co-inhibition experiments needs additional clarification. Accordingly, we have:

1) Revised the results section to emphasize that co-inhibition was lethal in ~17% of cases, and only surviving animals were used for behavioral experiments.

2) Revised the corresponding discussion section as follows: "Chronic MR inhibition was lethal in ~17% of cases, perhaps due to the physiological costs of sustained sleep loss. Intriguingly, most animals with chronic MR inhibition survived and were able to maintain highly engaged wakefulness. They also successfully encoded persistent memories despite severe and long-term sleep reductions (Extended Data Figs. 14-17). MR manipulations may thus provide a novel context to study the long-term effects of sleep loss, as well as the mechanisms that confer resilience to sleep deprivation."

Referee #2 (Remarks to the Author):

Note: For clarity, we have added numbers to each of the referee's comments.

1. Overall, the number of experiments made is amazing and it indeed seems that the median raphe GABAergic neurons plays a role in sleep induction. In a way, there are two many experiments shown to make such demonstration. It is therefore difficult to assimilate all of them. The will to provide a huge number of experiments to show that the study is going in the right direction is rather deserving it. Of course, the neuroanatomical data is crucial as well as the chemogenetic and Kir experiments but other ones are not that necessary. One key experiment is on the other hand still missing: the recordings of the Median raphe neurons. I'm indeed not convinced by the « in vitro » recordings. Even fiber photometry recordings which are way simpler would be more convincing. Of course, the best would be to provide unit recordings and to show the behavior of the Median raphe GABA and serotonin neurons across sleep-wake states.

We thank the referee for their feedback. We agree that *in vivo* approaches would complement our findings. Our slice electrophysiology experiments revealed sleep deprivation-induced changes in the intrinsic properties of sleep-promoting cells, including depolarized baseline membrane potential and lower firing threshold. Imaging approaches cannot uncover these mechanistic insights into cellular properties. On the other hand, calcium imaging or optrode recordings would clarify neuronal activity dynamics of genetically defined cells across behavioral states. These experiments are of high interest and will be the focus of future studies. Based on the referee's comments, we now state this in the discussion.

2. Below, my other concerns remaining in particular with regard to the cFos experiments.
Concerning the artifactual labeling near the ventricule, the new images are indeed very nice and indeed showing that there are a few cFos labeled cells near the ventricules. However, I'm not convinced at all that it corresponds to their staining in all their voxel Fig.s like Fig. R2 for the recovery condition. Indeed, it seems for example that the magenta above the lateral ventricule is in the white matter. It is well known that cFos immunostaining whatever the methods used give rise to artifactual labeling. To eliminate such artefactual labeling the authors would need to use a more classical method than the one they use. Classically, automatic counting is done for each cFos labeled cells using deep learning based on the form factor and the size of the cells.

We believe the peri-ventricular signals represent actual changes in Fos patterns, rather than artifacts. Extended Data Fig. 4 shows Fos+ cells in the medial, lateral, and dorsal walls of the ventricle. In a complementary approach, Fos TRAP also reveals tdTomato+ cells in the medial, lateral, and dorsal walls (Extended Data Fig. 4d), strongly indicating Fos activation (rather than artifactual signal) around the ventricle.

More generally, we agree with the referee that immunostaining can generate artifacts that must be excluded from automated quantifications. In fact, our strategy does minimize artifacts, with comparable results to machine learning approaches. Specifically, our approach constrains the detection of Fos+ nuclei based on size and shape (as suggested by the referee), by using a Laplacian of Gaussian (LoG) filter. LoG filters are ideal for detecting bright elliptical objects against contrasting backgrounds, and are thus well-suited to detect Fos+ nuclei (Kong et al 2013; Renier et al 2016; L'Esperance et al 2024). Size, intensity, and overlap parameters were adjusted to correctly detect strong Fos+ nuclei while excluding weak or background signals (ex: min_sigma=2, num_sigma=3, max_sigma=11, threshold=.075, overlap=0.97; See https://scikit-image.org/ for details).

Fig. R1 below displays example Fos imaging data and corresponding cell detection results based on a LoG filter. Strong nuclei are robustly detected, while background-level signals are excluded. Prompted by the referee's comments, we also trained a classifier to automatically detect Fos+ nuclei using Ilastik (https://www.ilastik.org/), and observed comparable results (**Fig. R1B**).

In summary, our strategy reliably detects Fos+ nuclei while excluding spurious labeling, and generates similar results to "classical methods" or machine learning-based pipelines. Based on the referee's comments, we have added additional details on our use of LoG filters to the methods section.

Fig. R1: Detection of Fos+ nuclei
(A) Partial hemisphere from a Fos immunostained and light-sheet imaged brain, in horizontal orientation. 50um Z-projection is shown from a novel objects SD3 sample, with Fos signal in grayscale.
(B) Top row: Higher magnification images of boxed areas in A.
Middle row: centroids of automatically detected Fos+ nuclei using a LoG filter.
Bottom row: Predicted Fos+ nuclei from a trained classifier in Ilastik. Where appropriate, dotted lines indicate tissue boundaries or landmarks to provide context. Cell counts for each method are provided at bottom left of each panel.

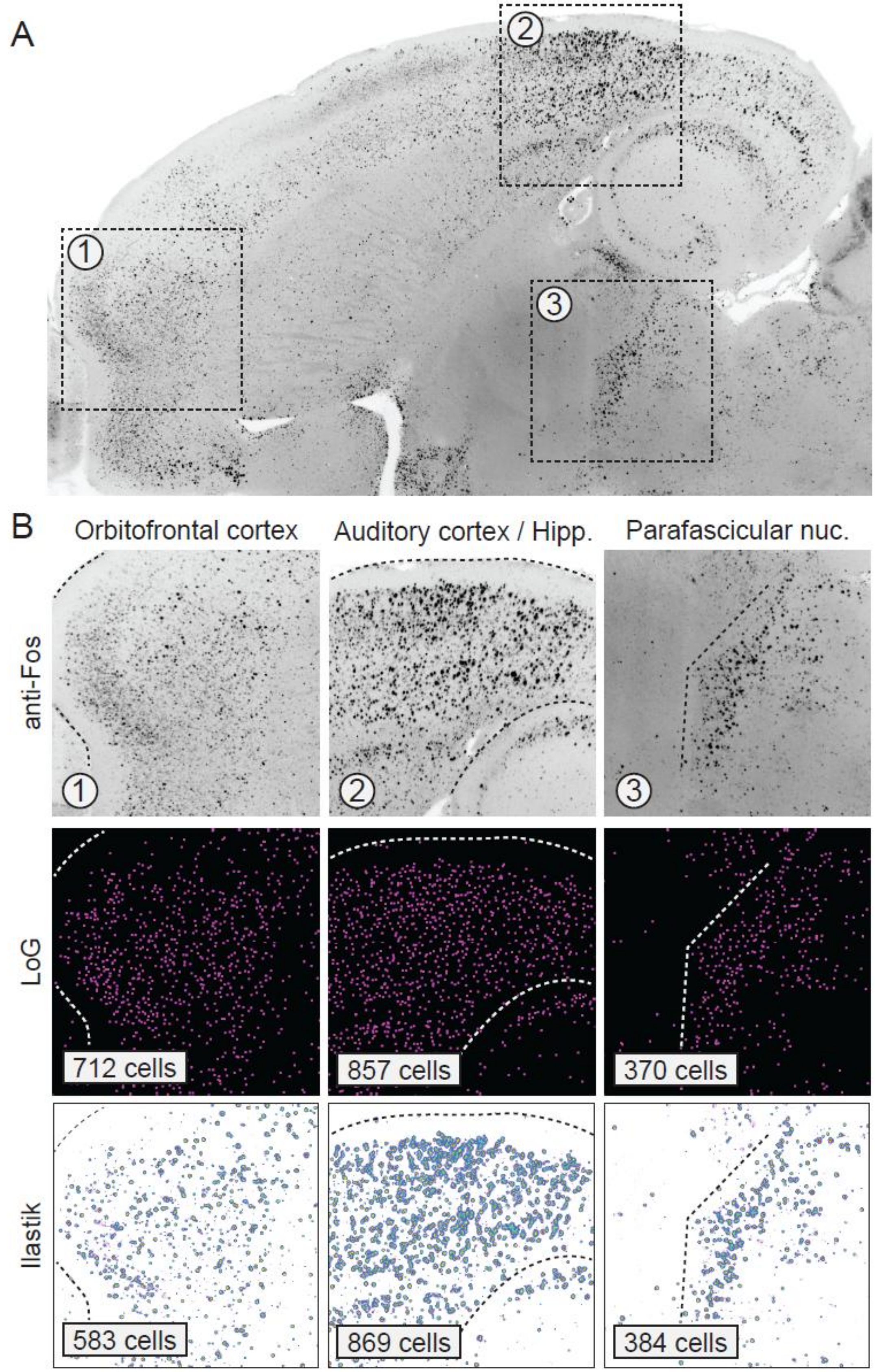

3. What I don’t buy in their analysis is that instead that use voxel analysis which is not strictly corresponding to the cells. By this mean, they loose the power of the method which is giving a cell resolution of the staining. Further, they cannot eliminate artifactual labeling. Then, they state that: « voxel-based quantifications more faithfully capture the data and are more suited to isolate sleep or wake-related Fos signals than quantifications across entire Allen Brain structures. » This sentence is interesting but wrong. The main advantage of the cFos method is that it is showing activated neurons at the cellular level. By this means, it is vastly superior to classical imaging. Therefore, to remove such accuracy with voxeling is a mistake. I agree with the authors that using just the Allen Brain Atlas is not enough since it is a very simplified atlas. But the best way is to look at the cFos labeling and to add accurate real subdivisions based on previous publications like those mentioned by the authors. Such method also allow to make corrections of the alignement between mice which always occur when making such study. The brains of different mice is indeed never fully aligned automatically. I personally dislike Fig. R10 since it looks like functional imaging and gives no ideas of the real density of the cFos cells. Please add to this Fig., a photomicrograph showing the cFos labeled cells.

Voxel-based analyses have been validated in numerous studies using volumetric brain imaging, and are standard in the field when comparing multiple brains or conditions (Kim et al 2015; Renier et al 2016; Kim et al 2017; Schneeberger et al 2019; Kirst et al 2020; Hansen et al 2021; Yamaguchi et al 2023; Gustison et al 2024; Kanatani et al 2024; Attarpour et al 2025; Zimmerman et al 2025; Yamashita et al 2026; and others. See **Fig. R2** below for examples). Voxel-based analyses are powerful because they allow comparison of numerous conditions in 3D space, while preserving potentially interesting sub-region topography for future investigation. In contrast, counting cells per Allen Brain Atlas region results in a major loss of “accuracy” by definition, and introduces inherent biases. The overarching goal of our Fos analyses was to allow rapid and unbiased identification of sleep/wake-correlated regions for further analyses. We believe replacing our current approach with region-based cell counts would provide a less nuanced, lower-resolution view of the results. Given that the other referees have described our results as “beautifully presented,” “interesting,” “extensive,” and “a valuable community resource,” we propose to maintain our current voxel-based analyses and refer to the previous studies cited above.

We are sorry to hear that the referee disliked Figure R10. We have updated it with examples of raw Fos immunostaining images as requested, as well as overlays of detected Fos+ nuclei registered to the Allen Brain atlas CCFv3 (**Fig. R3** below). The updated figure bridges the visual gap between detected Fos+ nuclei and the corresponding voxelized map. We hope this provides additional clarity.

Based on the referee’s comments, we have added additional details to the methods section, including how the pipeline we use combines SimpleElastix-based automated registration with user-guided corrections for high-fidelity alignments.

Fig. R2: Examples of voxel-based analyses in whole-brain datasets.
Eight examples of voxel-based analyses for whole brain immediate early-gene expression, cell type distribution, or structure. Figure is split over multiple pages.

(1) Yamashita...Ueda et al (2026).
A whole-brain single-cell atlas of circadian neural activity in mice. *Science* 391, eaea3381

(From Fig. 4)

[Redaction]
Figure Includes
Third Party Material

(2) Zimmerman...Witten et al (2025).
A neural mechanism for learning from delayed postingestive feedback. *Nature* 642, 700–709.

(From Fig. 1 and Extended Data Fig. 1)

[Redaction]
Figure Includes
Third Party Material

③ Attarpour...Goubran et al (2025).
A deep learning pipeline for three-dimensional brain-wide mapping of local neuronal ensembles in teravoxel light-sheet microscopy. *Nature Methods* 22, 600–611.

(From Fig. 4)

[Redaction]
Figure Includes
Third Party Material

④ Kanatani...Uhlen et al (2024).
Whole-brain spatial transcriptional analysis at cellular resolution. *Science* 386, 907-915.

(From Fig. 5)

[Redaction]
Figure Includes
Third Party Material

5 Yamaguchi...de Lecea et al (2023).
Dorsomedial and preoptic hypothalamic circuits control torpor. *Current Biology* 33(24), 5381–5389.

(From Fig. 1)

[Redaction]
Figure Includes
Third Party Material

6 Kirst...Renier et al (2020).
Mapping the Fine-Scale Organization and Plasticity of the Brain Vasculature. *Cell*, 180(4), 780–795.

(From Fig. 4)

[Redaction]
Figure Includes
Third Party Material

7. Kim...Osten et al (2017).
Brain-wide Maps Reveal Stereotyped Cell-Type-Based Cortical Architecture and Subcortical Sexual Dimorphism. *Cell*, 171(2), 456–469.

(From Fig. 6)

[Redaction]
Figure Includes
Third Party Material

8. Renier...Tessier-Lavigne et al (2016).
Mapping of Brain Activity by Automated Volume Analysis of Immediate Early Genes. *Cell*, 165(7), 1789–1802.

(From Fig. 6)

[Redaction]
Figure Includes
Third Party Material

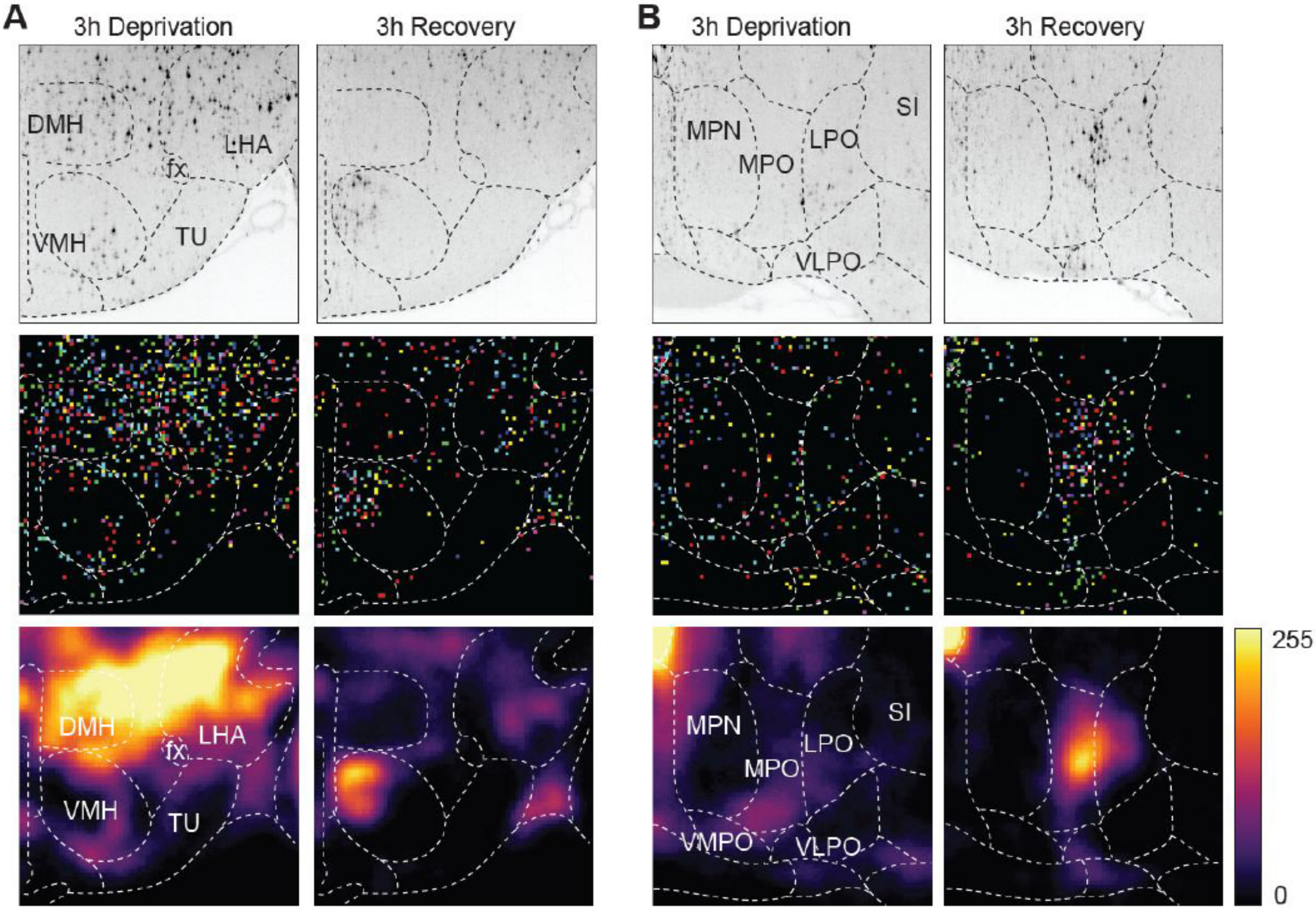

Fig. R3: Voxel vs region-based quantifications of Fos induction

(A) Top row: Example images of Fos distribution in the LHA and VMH 3h after sleep deprivation or 3h into the recovery period, from individual animals. Samples were whole-brains processed using iDisco+ and light-sheet imaged. Coronal images are reslices of horizontally imaged samples.

Middle row: Automatically detected Fos+ nuclei registered to the Allen Brain CCFv3, color-coded by sample. N=6 brains each for the 3h deprivation and 3h recovery timepoints. Bottom row: Average voxelized maps for each timepoint, highlighting sub-region patterns for VMH and LHA (A) and MPO/LPO (B). (C) Bottom panel, Fos+ nuclei detected for the entire LPO (open circles) or MPO (closed circles) across deprivation timepoints. The entire anterior-posterior extent of each region was included for quantification. Note absence of Type 2 response pattern and reversed temporal pattern relative to (B).

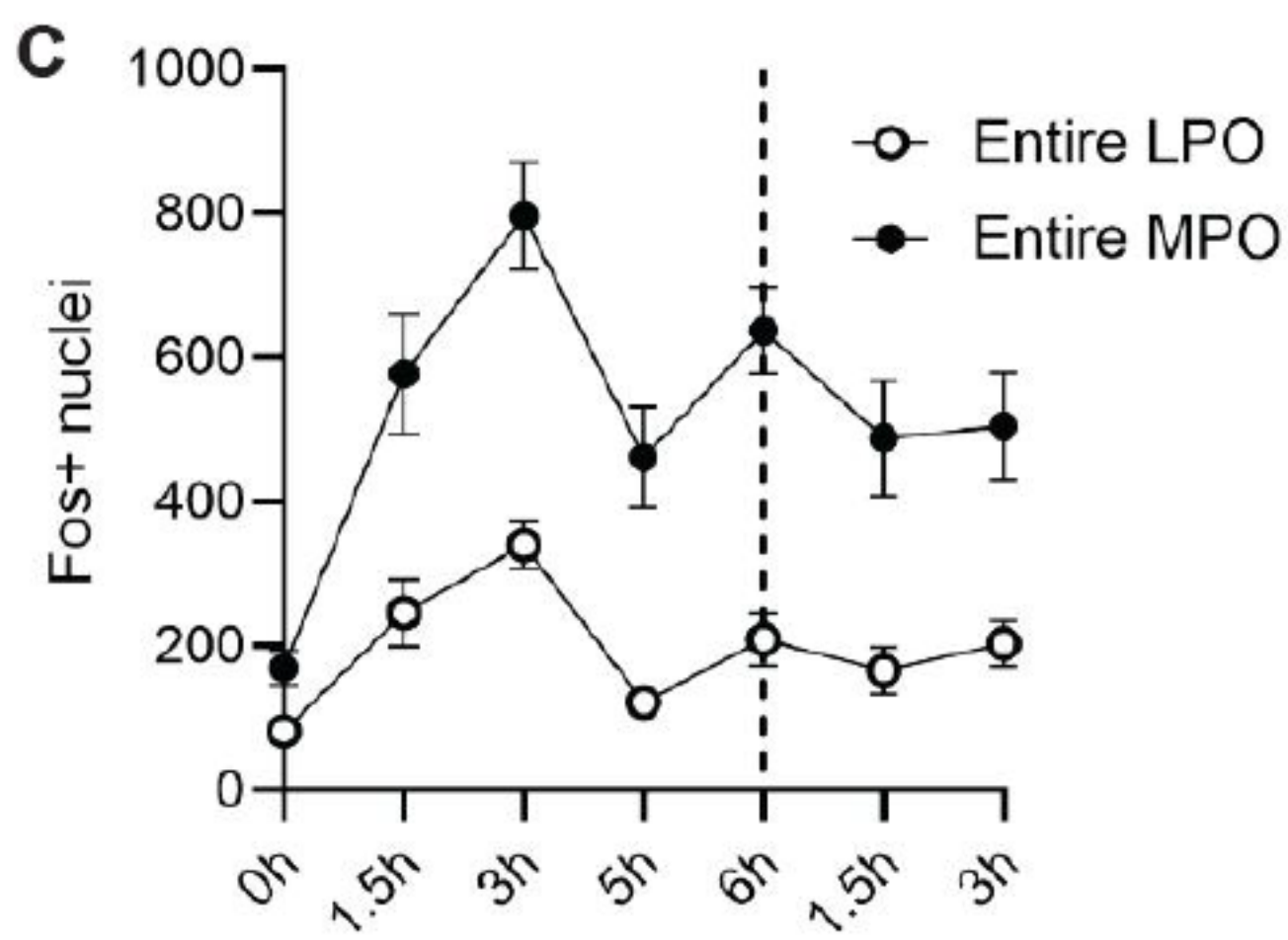

5. I'm also not convinced by their explanation that the decrease of cFos staining after 3h of deprivation is due to the fact that sensory-motor systems are less activated. Indeed, the longer you sleep-deprive the more you need to simulate the animals to keep them awake. In addition, across time different sensory-motor neurons are recruited in the cortex since the experience is always different and they introduce new objects and therefore induce the activation of different neurons over time.

We apologize for any confusion, and hope we can clarify. We did not make any strong claims about how "sensory-motor" information is represented or processed over time. Instead, we stated in the paper that these areas "correlate with sensory stimulation, novelty, or cognitive engagement rather than wake duration," because somatosensory, visual, and auditory cortices exhibited strong responses during sleep deprivation but minimal responses during unperturbed behavior. These Type 1 response patterns are consistent with the well-documented transient nature of immediate early gene activation during sensory, electrical, or pharmacological stimulation (Yap and Greenberg, 2018; Kushinsky et al 2024; Bisler et al 2002; Zangenehpour & Chaudhuri 2002; Bullitt, et al 1992; Tyssowski, et al 2018, and others). Furthermore, while Fos labeling in specific sensory areas peaks and declines after 1.5-3h deprivation, it still remains highly elevated relative to the 0h timepoint or to unperturbed circadian controls (Fig. 1d). In other words, Fos peaks at specific timepoints but does not immediately return to baseline, consistent with prolonged stimulation. Lastly, Type 1 response patterns were observed for both novel objects and reinforced grooming-based sleep deprivation (Fig. 1), suggesting that these patterns emerge independently of stimulus type.

Previous sleep deprivation experiments offer candidate mechanisms that may contribute to Type 1 response patterns. For example, intact locus coeruleus (LC) input is required for Fos, NGF1-A, and phospho-CREB induction in the cortex during sleep deprivation (Cirelli et al 1996). LC norepinephrine transmission becomes severely attenuated during sleep deprivation or naturally occurring long wake bouts (Bellisi et al 2016; Silverman et al 2024), which may contribute to Type 1 response patterns. Sleep deprivation can also increase the occurrence of local OFF periods in cortical neurons during wakefulness (Vyazovskiy et al 2011). These "local sleep" periods are thought to compromise cognitive performance, and may also contribute to Type 1 responses. Moreover, accumulation of extracellular adenosine during wakefulness may further affect the function of cortical circuits or their inputs (Porka-Heiskanen et al 2000; Greene et al 2017; Peng et al 2020; Bayazitov 2024).

Based on the referee's comments, we have revised the results section to make these points clearer.

6. I'm also not convinced by their answer concerning spotty labeling in the caudate nucleus. When you align many brain, it is impossible that it is so perfect you can see such very localized activation across animals. The authors need to verify their cFos staining to understand why they got this spotty labeling. I don't like their answer there since they simply did not control this point.

Parts of Fig. 1 and Extended Data Fig. 3 can appear "spotty" because of the data type. These figures indicate voxels that are classified and color-coded as Type 1-3, according to their response patterns during sleep deprivation experiments. In other words, the colors indicate the anatomical locations of the hierarchically clustered voxels, rather than a binarized map of Fos+ nuclei (as in **Fig. R3A-B** above, middle rows. Also see Example 1 in **Fig. R2**).

7. « In contrast, "CNO injection at the end of deprivation" would overlap with the naturally occurring decline in activation during the recovery period, thus making it difficult to interpret the results. »
This is a purely speculative statement if they did not test it.

We apologize for any confusion, but believe our statement is strongly supported by the data. First, Fos rapidly declines in the MR during the recovery period following sleep deprivation (Fig. 1, Extended Date Fig. 3). Second, Recovery TRAP labels 5-fold fewer cells relative to Deprivation TRAP (Fig. 2).
These observations strongly suggest that the MR is more activated during wakefulness than during sleep. The chemogenetic inhibition experiment in Fig. 3o-p tests the hypothesis that MR activation during wakefulness is required for increased sleep propensity. Consistent with this idea, we find that chemogenetic inhibition during sleep deprivation significantly decreases sleep attempts relative to controls.

8. « As for VI, both Fig. 4 and the additional data below demonstrate MR projections to VI, as well as suppression of Fos in VI. We now include this in an extended data Fig.. » This is a puzzling results, how the authors interpret these results? It cannot be a pathway implicated in wake or sleep control. Is it known that the serotonin neurons of the MnPo project to the VI? Again their voxel image is not convincing to me. They should look at the neurons and confirm that the cFos-labeled neurons are inside the VI only composed of motoneurons which are quite big.

In Fig. 4d-e, we use chemogenetic activation to expose potential targets of the MR. We hypothesized that regions with both MR axon terminal enrichment (Fig. 4a-c) and MR hM3Dq-dependent changes in Fos would be high-confidence targets. However, not all downstream targets necessarily contribute to sleep/wake control, and brain regions can participate in multiple behaviors. Thus, the role of MR projections to VI remains to be tested. Along these lines, we provide evidence that MR projections to the preoptic area promote sleep (Fig. 4f-h). Future experiments can more comprehensively characterize the cell types and functions of additional MR targets.

9. « the MR includes three non-overlapping cell types with distinct neurotransmitter identities. GABAergic Vgat+ cells represent 60% of MR cells, while glutamatergic Vglut2+ cells and serotonergic cells respectively comprise ~25% and ~10%." We concurrently cite the two relevant references: Sos et al 2017 and Szonyi et al 2019. We hope this provides additional clarity. » In fact, it has been reported that 5-HT neurons of the MR express vGlut3 (Ren et al., 2019). This might explain why they show many activated regions when they stimulated these neurons.

Vglut3 expression defines a subset of raphe serotonin neurons, and glutamate co-transmission in Sert+ cells is an interesting possibility. Future experiments can test the respective functions of glutamate or serotonin release from these neurons using conditional knockout of Sert and/or Vglut3, or other approaches. Based on the referee's comments, we now mention both the Ren et al. study and a previous paper by Okaty et al. in the discussion.

10. I realized based on their answer to reviewer 3 that EEG and EMG recordings have not been done in all mice in particular in the cFos experiments. They need to clearly state which mice were recorded in the paper since it is a crucial information. Indeed without sleep recordings how they can be sure that the mice were not sleeping or on the contrary awake?

As mentioned in our response to Referee 3, implanting all ~160 animals used in the Fig.1 Fos mapping experiments with EEG electrodes would be prohibitively labor-intensive. However, EEG data was collected for unperturbed circadian experiments. This allowed us to quantify baseline sleep vs wake-correlated responses in Extended Data Fig. 3c. For sleep deprivation Fos experiments, EEG data was not collected for all animals. However, animals were constantly monitored to ensure continued wakefulness during the 6h sleep deprivation period. Fig. 1e and Extended Data Fig. 1 demonstrate both the effectiveness of our sleep deprivation methods and the reliable increase in sleep during the recovery period. EEG recordings were used to quantify sleep behavior in all other experiments. Based on the referee's comments, we have updated our methods with these details.

11. How the authors explain that in Fig 1.d they don't show high activation in the cortex during the night since Wake is predominant at that time?

Fig. 1d actually shows that cortical areas such as S1, V1, and A1 exhibit high activation in all sleep deprivation experiments, including dark phase sleep deprivation. Indeed, these cortical areas show comparable responses during light phase and dark phase novel objects-based sleep deprivation.

12. Extended data 2 Fig., I'm still not convinced by that Fig.. The morphology of cFos labeled cells is not clear cut and should be. It seems there is a high background. The delimitations of the structures is also not very convincing in particular for aMPO. It is needed to show the bottom of the brain to localize the nucleus.

For more details on the aMPO, we direct the referee to Extended Data Fig. 18, which provides additional anatomical context and cell type characterization.

13. Extended Data Fig. 3a, their LHA seems to include the zona incerta. Did they look whether it is not rather zona incerta since there is a recent paper showing that it also contain W-active sleep inducing neurons?

As the referee states, Extended Data Fig. 3a shows wake-associated activation patterns within both the LHA and ZI. We assume the reviewer refers to the LHA activation experiments. LHA deprivation TRAP cell activation strongly promoted wakefulness (Extended Data Fig. 7e). This effect likely corresponds to the LHA rather than the ZI, as Cre-dependent hM3Dq was virally delivered to the LHA, and TRAP labeling is predominantly within the LHA (Extended Data Fig. 7b-d). Our results are thus consistent with those of Liu et al 2017 and Lee et al 2025, who show that Lhx6+ ZI cells or Lhx6+ ZI-targeting mRE cells both promote NREM sleep rather than wakefulness.

14. I finally wonder how the authors explain that the Kir mice freeze less during the shocks (FigR5)

We speculate that Kir2.1 animals are more highly aroused, as stated in the paper. Consistent with this idea, Kir2.1 animals exhibit increased locomotion between shocks (Extended Data Fig. 17d and **Fig. R4** below). Several other observations from the paper further corroborate this idea:

1) Kir2.1 animals reacted to shocks with greater vigor, traversing greater horizontal distances and jumping more frequently in response to each shock (Extended Data Fig. 17c-d).
2) Kir2.1 animals were more mobile during both training and recall (Extended Data Fig. 17e). Indeed, reduced freezing during recent recall was primarily due to increased exploration when Kir2.1 mice were first reintroduced to the training context (Extended Data Fig. 17g-i).
3) Kir2.1 animals were more mobile in the open field and elevated plus maze assays (Extended Data Fig. 15).
4) Kir2.1 animals exhibited greater engagement with wood blocks placed in the home cage (Extended Data Fig. 16).
5) Kir2.1 animals spent a greater proportion of time in high theta wakefulness than controls (Extended Data Fig. 14).

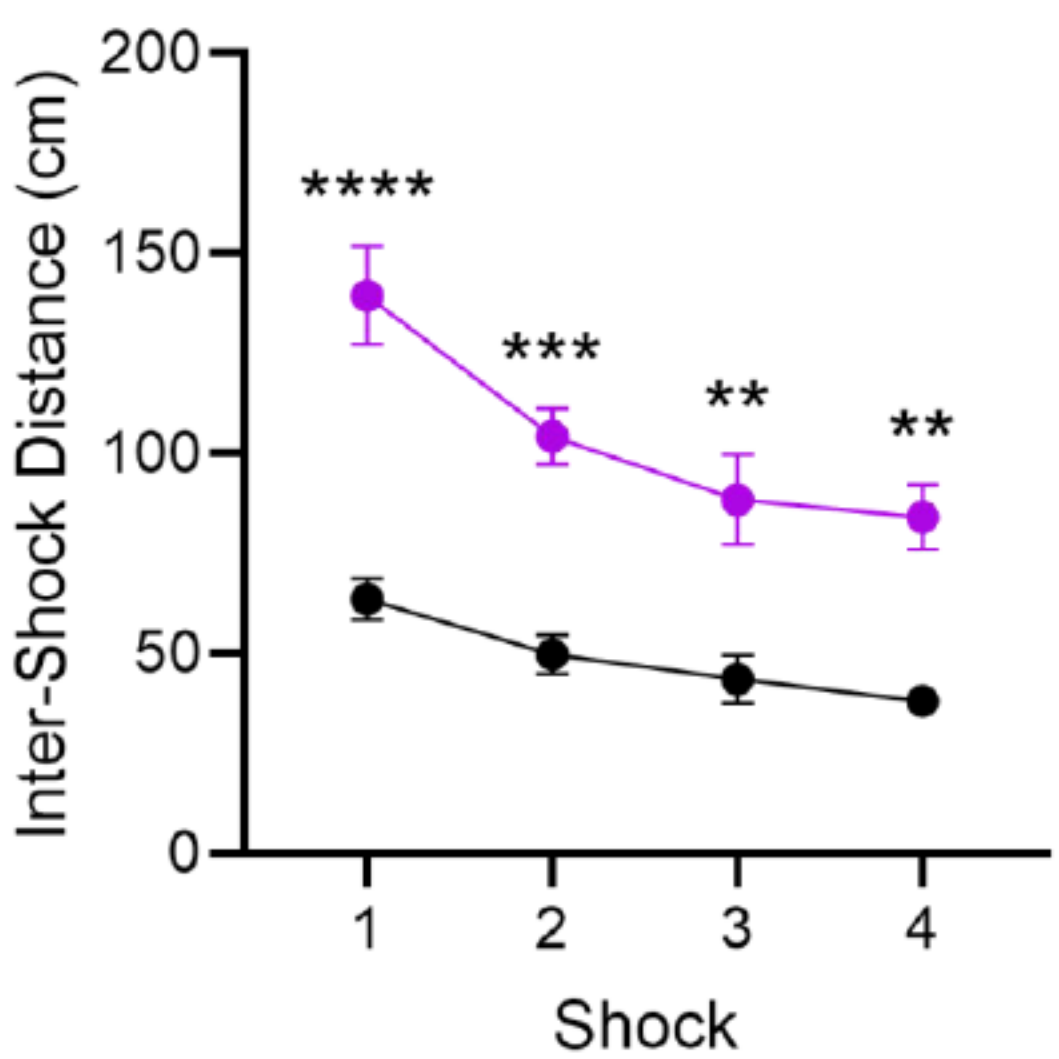

Fig. R4: Kir2.1 animals exhibit greater locomotion in between shocks
Total distance traveled in the 30 seconds following each of the 4 first training shocks. Black, Control, n=8. Magenta, Kir2.1, n=11. **p<0.01, ***p<0.001, ****p<0.0001, Two-Way ANOVA. As video data were only collected for 300 seconds, the full 30 seconds following the 5th shock was not quantified.

In summary, the demonstration that the median raphe contains neurons active during wake involved in sleep induction is new and interesting. However, there are still problems in the data shown and the manuscript could be streamlined. The addition of « in vivo » unit recordings is key to my point of view and is still lacking.

We thank the reviewer for their support and suggestions, and hope we have adequately addressed their remaining concerns in the detailed responses above.

References

- Attarpour, et al. (2025). A deep learning pipeline for three-dimensional brain-wide mapping of local neuronal ensembles in teravoxel light-sheet microscopy. *Nat Methods* 22, 600–611 (2025). https://doi.org/10.1038/s41592-024-02583-1

- Bayazitov, et al. (2024). Sound-evoked adenosine release in cooperation with neuromodulatory circuits permits auditory cortical plasticity and perceptual learning. *Cell reports*, *43*(2), 113758. https://doi.org/10.1016/j.celrep.2024.113758

- Bellesi, et al. (2016). Region-Specific Dissociation between Cortical Noradrenaline Levels and the Sleep/Wake Cycle. *Sleep*, *39*(1), 143–154. https://doi.org/10.5665/sleep.5336

- Bisler, et al. (2002). Expression of c-Fos, ICER, Krox-24 and JunB in the whisker-to-barrel pathway of rats: time course of induction upon whisker stimulation by tactile exploration of an enriched environment. *Journal of chemical neuroanatomy*, *23*(3), 187–198. https://doi.org/10.1016/s0891-0618(01)00155-7

- Bullitt, et al (1992). The effect of stimulus duration on noxious-stimulus induced c-fos expression in the rodent spinal cord. *Brain research*, *580*(1-2), 172–179. https://doi.org/10.1016/0006-8993(92)90941-2

- Cirelli, et al.(1996) Neuronal Gene Expression in the Waking State: A Role for the Locus Coeruleus. Science274,1211-1215(1996).DOI:10.1126/science.274.5290.1211

- Greene, R. W., et al. (2017). The adenosine-mediated, neuronal-glial, homeostatic sleep response. *Current opinion in neurobiology*, *44*, 236–242. https://doi.org/10.1016/j.conb.2017.05.015

- Gustison, et al. (2024). Sexual coordination in a whole-brain map of prairie vole pair bonding. *eLife*, *12*, RP87029. https://doi.org/10.7554/eLife.87029

- Hansen, et al. (2021). Whole-brain activation signatures of weight-lowering drugs. *Molecular metabolism*, *47*, 101171. https://doi.org/10.1016/j.molmet.2021.101171

- Yamashita et al (2025). A whole-brain single-cell atlas of circadian neural activity in mice. Science 391, eaea3381(2026). DOI:10.1126/science.aea3381

- Kanatani et al (2024). Whole-brain spatial transcriptional analysis at cellular resolution. Science 386,907-915(2024).DOI:10.1126/science.adn9947

- Kim, et al. (2015). Mapping social behavior-induced brain activation at cellular resolution in the mouse. Cell reports, 10(2), 292–305. https://doi.org/10.1016/j.celrep.2014.12.014

- Kim, et al. (2017). Brain-wide Maps Reveal Stereotyped Cell-Type-Based Cortical Architecture and Subcortical Sexual Dimorphism. *Cell*, *171*(2), 456–469.e22. https://doi.org/10.1016/j.cell.2017.09.020

- Kirst, et al. (2020). Mapping the Fine-Scale Organization and Plasticity of the Brain Vasculature. *Cell*, *180*(4), 780–795.e25. https://doi.org/10.1016/j.cell.2020.01.028

- Kong, et al (2013). "A Generalized Laplacian of Gaussian Filter for Blob Detection and Its Applications," in IEEE Transactions on Cybernetics, vol. 43, no. 6, pp. 1719-1733, Dec. 2013, doi: 10.1109/TSMCB.2012.2228639

- Kushinsky, et al (2024). Daily light-induced transcription in visual cortex neurons drives downward firing rate homeostasis and stabilizes sensory processing. *Cell reports*, *43*(9), 114701. https://doi.org/10.1016/j.celrep.2024.114701

- Lee et al. (2025) Sleep need–dependent plasticity of a thalamic circuit promotes homeostatic recovery sleep. *Science* 388,eadm8203(2025).DOI:10.1126/science.adm8203

- L'Esperance et al. (2024). Functional Connectivity Favors Aberrant Visual Network c-Fos Expression Accompanied by Cortical Synapse Loss in a Mouse Model of Alzheimer's Disease. J Alzheimers Dis. 2024;101(1):111-131. doi: 10.3233/JAD-240776

- Liu, et al (2017). Lhx6-positive GABA-releasing neurons of the zona incerta promote sleep. *Nature* **548**, 582–587 https://doi.org/10.1038/nature23663

- Okaty, B. W. et al (2015). Multi-Scale Molecular Deconstruction of the Serotonin Neuron System. *Neuron* **88**, 774–791 https://doi.org/10.1016/j.neuron.2015.10.007

- Peng et al.*(2020).* Regulation of sleep homeostasis mediator adenosine by basal forebrain glutamatergic neurons.Science**369**,eabb0556(2020).DOI:10.1126/science.abb0556

- Ren, J. et al (2019). Single-cell transcriptomes and whole-brain projections of serotonin neurons in the mouse dorsal and median raphe nuclei. *Elife* **8**. https://doi.org/10.7554/eLife.49424

- Renier, et al. (2016). Mapping of Brain Activity by Automated Volume Analysis of Immediate Early Genes. *Cell*, *165*(7), 1789–1802. https://doi.org/10.1016/j.cell.2016.05.007

- Porkka-Heiskanen, et al. (2000). Brain site-specificity of extracellular adenosine concentration changes during sleep deprivation and spontaneous sleep: an in vivo microdialysis study. *Neuroscience*, *99*(3), 507–517. https://doi.org/10.1016/s0306-4522(00)00220-7

- Schneeberger, et al. (2019). Regulation of Energy Expenditure by Brainstem GABA Neurons. *Cell*, *178*(3), 672–685.e12. https://doi.org/10.1016/j.cell.2019.05.048

- Silverman et al. (2025) Activation of locus coeruleus noradrenergic neurons rapidly drives homeostatic sleep pressure.Sci. Adv.11,eadq0651(2025).DOI:10.1126/sciadv.adq0651

- Swaney, J. *et al.* Scalable image processing techniques for quantitative analysis of volumetric biological images from light-sheet microscopy. *bioRxiv*, 576595 (2019). https://doi.org/10.1101/576595

- Tyssowski, et al (2018). Different Neuronal Activity Patterns Induce Different Gene Expression Programs. *Neuron*, *98*(3), 530–546.e11. https://doi.org/10.1016/j.neuron.2018.04.001

- Vyazovskiy, et al. (2011). Local sleep in awake rats. *Nature*, *472*(7344), 443–447. https://doi.org/10.1038/nature10009

- Yamaguchi, et al. (2023). Dorsomedial and preoptic hypothalamic circuits control torpor. *Current biology 33*(24), 5381–5389.e4. https://doi.org/10.1016/j.cub.2023.10.076

- Yamashita, et al. (2026). A whole-brain single-cell atlas of circadian neural activity in mice. *Science 391* (6787), eaea3381. https://doi.org/10.1126/science.aea3381

- Yap & Greenberg (2018). Activity-Regulated Transcription: Bridging the Gap between Neural Activity and Behavior. Neuron, 100(2), 330–348. https://doi.org/10.1016/j.neuron.2018.10.013

- Zangenehpour & Chaudhuri, A. (2002). Differential induction and decay curves of c-fos and zif268 revealed through dual activity maps. *Brain research. Molecular brain research*, *109*(1-2), 221–225. https://doi.org/10.1016/s0169-328x(02)00556-9

- Zimmerman, et al. (2025). A neural mechanism for learning from delayed postingestive feedback. *Nature*, *642*(8068), 700–709. https://doi.org/10.1038/s41586-025-08828-z
